# Insights into the evolution of mammalian telomerase: Platypus *TERT* shares similarities with genes of birds and other reptiles and localizes on sex chromosomes

**DOI:** 10.1186/1471-2164-13-216

**Published:** 2012-06-01

**Authors:** Radmila Hrdličková, Jiří Nehyba, Shu Ly Lim, Frank Grützner, Henry R Bose

**Affiliations:** 1 Section of Molecular Genetics and Microbiology, School of Biological Science, and Institute for Cellular and Molecular Biology, University of Texas at Austin, Austin, TX 78712-1095, USA; 2 The Robinson Institute, School of Molecular and Biomedical Science, The University of Adelaide, Adelaide, 5005 SA, Australia

**Keywords:** Platypus, TERT, Telomerase, Alternative splicing, Telomeres, Sex chromosomes

## Abstract

**Background:**

The *TERT* gene encodes the catalytic subunit of the telomerase complex and is responsible for maintaining telomere length. Vertebrate telomerase has been studied in eutherian mammals, fish, and the chicken, but less attention has been paid to other vertebrates. The platypus occupies an important evolutionary position, providing unique insight into the evolution of mammalian genes. We report the cloning of a platypus *TERT* (Oan*TERT*) ortholog, and provide a comparison with genes of other vertebrates.

**Results:**

The Oan*TERT* encodes a protein with a high sequence similarity to marsupial TERT and avian TERT. Like the TERT of sauropsids and marsupials, as well as that of sharks and echinoderms, OanTERT contains extended variable linkers in the N-terminal region suggesting that they were present already in basal vertebrates and lost independently in ray-finned fish and eutherian mammals. Several alternatively spliced Oan*TERT* variants structurally similar to avian *TERT* variants were identified. Telomerase activity is expressed in all platypus tissues like that of cold-blooded animals and murine rodents. Oan*TERT* was localized on pseudoautosomal regions of sex chromosomes X3/Y2, expanding the homology between human chromosome 5 and platypus sex chromosomes. Synteny analysis suggests that *TERT* co-localized with sex-linked genes in the last common mammalian ancestor. Interestingly, female platypuses express higher levels of telomerase in heart and liver tissues than do males.

**Conclusions:**

Oan*TERT* shares many features with *TERT* of the reptilian outgroup, suggesting that Oan*TERT* represents the ancestral mammalian *TERT*. Features specific to *TERT* of eutherian mammals have, therefore, evolved more recently after the divergence of monotremes.

## Background

Telomeres are specialized DNA-protein structures at the end of linear chromosomes [1]. Telomeres are important for protecting chromosomes from recombination, and fusion, and also play a role in cellular signaling following DNA damage. As a result of the inability of DNA polymerases to replicate chromosomal ends, telomeres shorten with each subsequent cell division. This progressive telomere shortening ultimately leads to cell growth arrest and senescence [2]. The reduction in telomere length is compensated for by the enzyme telomerase, which is composed of a catalytic subunit (TERT) with reverse transcriptase activity and an RNA template (TR) [3,4]. In addition to this canonical function, telomerase stimulates cell proliferation, protects against oxidative damage and apoptosis, and modulates gene expression (for review see [5-7]).

Telomerase plays a critical role in aging and cancer in vertebrates [8,9]. In normal somatic cells telomerase is downregulated, and only cells with high proliferation rates such as male germ line cells, stem cells and cells of the immune system retain high levels of telomerase activity [10]. Most human cancer cells express elevated levels of telomerase which is critical for tumor development [11]. The expression and activity of telomerase is tightly regulated principally at the level of *TERT* transcription (for review see [12]). Numerous *TERT* alternatively spliced (AS) variants have been identified in both vertebrates and plants [13]. The expression of human AS variants is regulated during development and carcinogenesis [14-17]. With the exception of the human AS *TERT* variant α, which is proposed to be a dominant-negative inhibitor of telomerase activity, the function of the *TERT* AS variants remains to be determined [18,19].

*TERT* genes were initially cloned from *Euplotes aediculatus* and *Saccharomyces cerevisiae*[20,21]. Other *TERT* genes from yeast – *Schizosaccharomyces pombe* and *Candida albicans*[22,23], and protozoa – *Giardia lambliaOxytrichaTetrahymenaParameciumLeishmania* and *Plasmodium* have been subsequently described [24-29]. Several *TERT* genes have been cloned from plants – *Oryza sativaAsparagales*, and *Arabidopsis*[30-33]. The protostomian *TERT* genes have been identified in a number of insect species including *Apis melliferaBombyx mori* and *Tribolium castaneum*, and the nematode *Caenorhabditis elegans*[34-36]. Recently, the *TERT* gene was cloned from an echinoderm, the purple sea urchin (*Strongylocentrotus purpuratus*), and urochordates, the sea squirt species *Ciona intestinalis* and *C. savignyi*[37,38]. Orthologs of *TERT* have been identified in many vertebrates including human, dog, mouse, rat, hamster, chicken, frog (*Xenopus laevis*), and several fish (*Danio rerioTakifugu rubripesNothobranchius furzeri, Oryzias latipes, O. melastigma,* and *Epinephelus coioides*) [22,39-48]. The *TERT* gene, however, has not been characterized in the basal group of mammals, the egg-laying monotremes (platypuses and echidnas).

The platypus, *Ornithorhynchus anatinus*, is an important species for evolutionary studies which possesses a unique combination of mammalian and reptilian features (for reviews see [49-51]). Platypuses lay eggs, but produce milk from mammary glands. Their average body temperature is lower (about 32°C) than other mammals. Many anatomical features of their reproductive system resemble birds including sperm shape and ovarian structure. The platypus genome project has also revealed many similarities between monotreme and avian genomes including genome size and repeat content [52]. Interestingly, monotreme sex chromosomes share high number of orthologues with chicken sex chromosomes but not with the eutherian X chromosome further supporting a special evolutionary position of this species [53-55]. To investigate the evolution of vertebrate *TERT* we cloned and then characterized the platypus ortholog.

## Results

The platypus occupies an important position in the evolution of the synapsid branch of amniotes providing unique insight into the evolution of mammalian genes. In this work we report the cloning of a platypus *TERT* ortholog, and provide an evolutionary comparison with TERT proteins of other vertebrates. We identified alternatively spliced forms and determined the expression pattern of platypus *TERT* (Oan*TERT*) mRNA and telomerase activity in various platypus tissues. The chromosomal localization of the Oan*TERT* gene and length of telomeres in the platypus were also defined. Telomerase activity in heart and liver in platypus females and males was compared. All these analyses are presented in the context of the evolution of the vertebrate *TERT* gene and its role in the regulation of telomerase activity.

### Cloning and characterization of platypus *TERT* cDNA sequences

Telomere repeats were detected at the termini of platypus chromosomes by fluorescence *in situ* hybridization (FISH) suggesting that the platypus encodes a functional *TERT* gene [53,56]. The platypus genome project identified an Oan*TERT* locus [52]. However, in the current NCBI database (as of August 29, 2011) this locus [GenBank:LOC100074692] is classified as a pseudogene due to a number of frameshifts and insertions of repetitive mobile elements in the regions corresponding to exon 1 and 2. Therefore, we cloned the platypus *TERT* cDNA by RT-PCR using ovarian and brain RNA of an adult platypus and primers based on the selected sequences of the genomic contig containing LOC100074692 [GenBank:NW_001794359.1] (Additional file 1 and Additional file 2: Table S1c and Figure S1a). The 5′ half of the cDNA does not correspond exactly to the released genome sequence due to a misalignment in the 3′ region of the predicted first exon and major gaps in the sequence corresponding to the predicted second exon (Additional file 2: Figure S1b). The positions of the gaps and misalignments correspond to regions of a very GC-rich DNA sequence of low complexity, suggesting that the assembly of the Oan*TERT* locus contains errors due to the complicated secondary structure of the *TERT* gene as well as the high levels of repetitive sequences found in the platypus genome [52]. The alignment of the cDNA sequence with the genome sequence suggest that the platypus *TERT* is encoded by 16 exons similar to other vertebrate *TERT* genes [13].

The assembled cDNA of Oan*TERT* contains the 4522 nucleotide (nt) sequence with 2 nt of the 5′ UTR, a reading frame of 3891 nt including start and stop codons, and 629 nt of the 3′ UTR (Additional file 2: Figures S1c, S1d, S1e). The UTR sequences are not complete. If the closest of two possible polyadenylation signal sequences (AATAGA, AAGAAA) located in genomic DNA are used to terminate transcription, then the size of platypus 3′ UTR would be 700–800 bp, in the size range of the 3′ UTRs found in *TERT* genes of other vertebrates. The open reading frame (ORF) encodes an Arg/Leu-rich protein of 1296 amino acids with a predicted molecular weight of 146 kDa. The successful cloning of Oan*TERT* cDNA indicates that platypus expresses an mRNA with a continuous reading frame that encodes a functional TERT protein.

### The position of platypus *TERT* in the evolution of vertebrate *TERT* genes

To evaluate the evolutionary relationship of OanTERT to other TERT proteins we have aligned its amino acid sequence with available TERT sequences of different metazoan species. Most of the TERT sequences (20 sequences) were derived from annotated GenBank files. Five TERT sequences, those of chimpanzee (Ptr), elephant (Laf), opossum (Mdo), zebra finch (Tgu), and anole (Aga) were molecular models from GenBank or Ensembl and these were subjected to small scale corrections as described in Methods and Additional file 3. Four TERT sequences, those of wallaby (Meu), elephant shark (Cmi), leech (Hro), and hydra (Hma) were assembled *de novo* using the genomic data from GenBank (Methods and Additional file 3).

**Figure 1:**
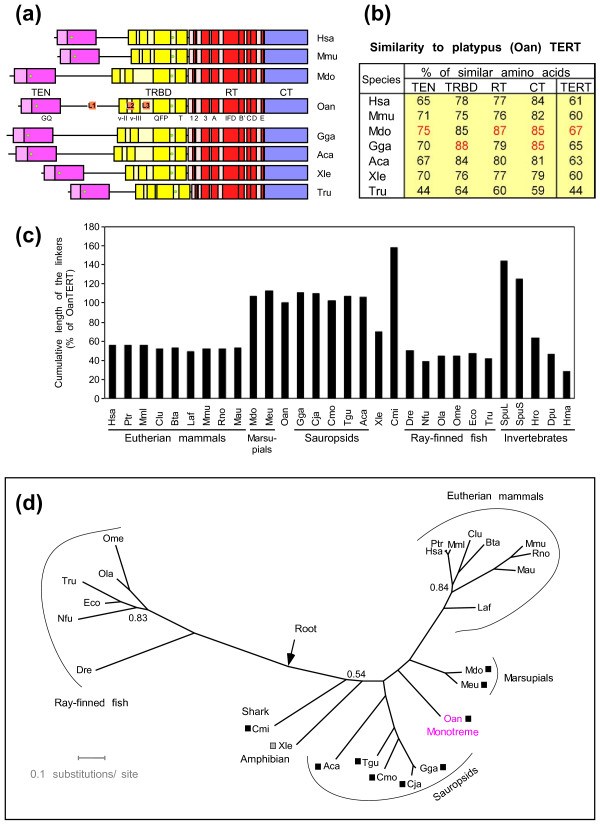
**Evolutionary relationship of platypus TERT.** (**a**) Comparison of the protein domains and motifs in the platypus TERT protein (Oan) with seven TERT proteins of selected vertebrates: Hsa - human (*Homo sapiens*), Mmu - mouse (*Mus musculus*), Mdo - short-tailed opossum (*Monodelphis domestica*), Gga - domestic chicken (*Gallus gallus*), Aca - green anole (*Anolis carolinensis*), Xle *-* African clawed toad (*Xenopus laevis*), Tru - pufferfish (*Takifugu rubripes*). Different colors indicate the four different domains (TEN, TRBD, RT, CT), with the darker tone indicating conserved motifs within the domains. The names of the motifs are depicted under the platypus TERT schema. L1, L2, and L3 designate three linker regions of variable length and sequence. The two green filled circles show the boundaries of the regions encoded by the second *TERT* exon, including the putative second exon of platypus. (**b**) The percentage of similar amino acids at the same positions in the aligned sequences of TERT proteins of seven vertebrate species shown in panel (a) relative the platypus TERT protein. The percentages calculated relative to the length of platypus sequence are shown for separate conserved domains (TEN, TRBD, RT, and CT) and the full-length protein (TERT). TRBD domain sequence excludes sequences of linkers L2, L3. (**c**) Cumulative length of the three linker regions (L1, L2, L3) in the TERT proteins of different species is shown as percentage of the length of these regions in platypus TERT. The lengths were calculated based on the alignment of the TERT proteins of different species shown in Figure S3 and on the definition of the three linker regions shown in Figure 1a and Figure S2. The graph includes TERT proteins shown in panel (a) and following additional proteins: Ptr - chimp (*Pan troglodytes*), Mml - macaque rhesus (*Macaca mulatta*), Clu - dog (*Canis lupus*), Bta - cow (*Bos taurus*), Laf - African elephant (*Loxodonta africana*), Rno - rat (*Rattus norvegicus*), Mau - hamster (*Mesocricetus auratus*), Meu - wallaby (*Macropus eugenii*), Cja - Japanese quail (*Coturnix japonica*), Cmo - Muscovy duck (*Cairina moschata*), Tgu - zebra finch (*Taeniopygia guttata*), Cmi - elephant shark (*Callorhinchus milii*), Dre - zebrafish (*Danio rerio*), Nfu - killifish (*Nothobranchius furzeri*), Ola - medaka (*Oryzias latipes*), Ome - medaka (*Oryzias melastigma*), Eco - grouper (*Epinephelus coioides*), SpuL and SpuS - long and short form TERT of sea urchin (*Strongylocentrotus purpuratus*), Hro - leach (*Helobdella robusta*), Dpu - water flee (*Daphnia pulex*), and Hma - hydra (*Hydra magnipapillata*). Insect, nematode, and urochordate TERTs were not included because these proteins do not contain all four basal structural domains of the vertebrate TERT protein [38,57]. (**d**) Phylogenetic tree of vertebrate TERT proteins constructed by the Bayesian inference method. The construction is based on the alignment shown in the Additional file 5: Figure S3. For the abbreviation of protein species origin see the legend to panels (a) and (c). All these proteins were presumably full-length except the chimp (98% complete), opossum (99.5% complete), wallaby (97% complete), and elephant shark (approximately 80% complete). Sea urchin SpuL TERT was used to determine the root of the tree (indicated by the arrow, actual branch with SpuL not shown). Numbers shown at the corresponding branches are clade credibility values. Because most of the branches had clade credibility equal to 1.00, only those with the value <1.00 are shown. The long and medium linkers are indicated by squares by the abbreviated species names. The black squares indicate TERT proteins with long linkers (the L1, L2 and L3 linkers reaching 100%-160% of the length of platypus linkers). A TERT protein with the linkers of medium length (60%-99% of the platypus linkers) is marked by gray square.

Initial evaluation was focused on the evolutionary conservation of the functional domains in OanTERT and included full-length TERT sequences of platypus and seven species representing different vertebrate systematic groups (Figure 1a). The analysis included platypus (Oan), human (Hsa), mouse (Mmu), gray short-tailed opossum (Mdo), domestic chicken (Gga), green anole lizard (Aca), African clawed toad (Xle), and pufferfish (Tru) proteins. Conserved domains and motifs detected in the aligned sequences are shown in Figure 1a (see also Additional file 4: Figure S2). Platypus TERT contains all four essential domains present in vertebrate TERT proteins: TEN (telomerase essential N-terminal), TRBD (telomerase RNA-binding domain), RT (reverse transcriptase), and CT (C-terminal) [4]. Inside these domains all of the conserved motifs were found, including the GQ motif in the TEN domain, motifs v-II, v-III, QFP, and T in the TRBD, and motifs 1, 2, 3, A, IFD, B′, C, D, and E in the RT domain. In many of these conserved domains as well as in the entire molecule the platypus TERT protein has the highest sequence similarity to the marsupial (opossum) TERT with the next highest similarity with birds and the lizard, followed by eutherian mammals (human, mouse) and the amphibian (toad) (Figure 1b). 

The main differences in TERT primary structure among the vertebrate species are in the length and the sequence of the three linker regions: linker L1 between TEN and TRBD, and linkers L2 and L3 inside the TRBD, between motifs v-II and v-III, and v-III and QFP, respectively (Additional file 4: Figure S2). Analysis of TERT protein sequences of 28 metazoan species revealed that they fall into three different groups based on the size of linkers (Figure 1c). Those with long linker elements have cumulative linker size equal or longer than platypus and include marsupials, sauropsids, a shark (Cmi) and an invertebrate - sea urchin (Spu). Short TERT proteins have cumulative linker size shorter than 60% of the size of these regions in platypus TERT. These proteins are found in all eutherian mammals and ray-finned fish and some invertebrates. Only two species have linker size intermediate between these two extremes - an amphibian (Xle) and an invertebrate (Hro). The shorter TERT proteins also often contain small reductions in the size of the more conserved parts of the protein, especially in the beginning of the RT domain 3 (Additional file 4: Figure S2).

To further refine the evolutionary position of the Oan*TERT* gene we have constructed phylogenetic tree of vertebrate TERT sequences (Figure 1d). The tree is based on the alignment of the longest available sequence for each protein. Setting gap tolerance to 0%, we have removed all aligned columns that contained gaps. These gaps were results either of low conservation or incompleteness of sequences. The tree confirmed that monotreme TERT is most closely related to the marsupial TERT. The monotreme and marsupial TERT proteins assume an intermediate position between TERTs of sauropsids (including birds) and the TERTs of eutherian mammals indicating that they share sequence similarities with the proteins from both of these groups. Interestingly, both monotreme and marsupial TERTs are positioned closer to the TERT of the common ancestor of sauropsids than that of the ancestor of eutherian mammals. The tree also indicates a close relationship of all vertebrate TERT proteins with long linkers as these are concentrated in one part of the tree close to base of vertebrate tree. Because the tree is constructed mostly from the sequence of the conserved regions, this analysis suggests that the relationship of the vertebrate proteins with long linkers is also paralleled by the sequence similarities in conserved domains.

In conclusion, monotreme and marsupial TERT proteins are evolutionary closely related to sauropsid TERT.

### Identification of alternatively spliced *TERT* variants

**Figure 2:**
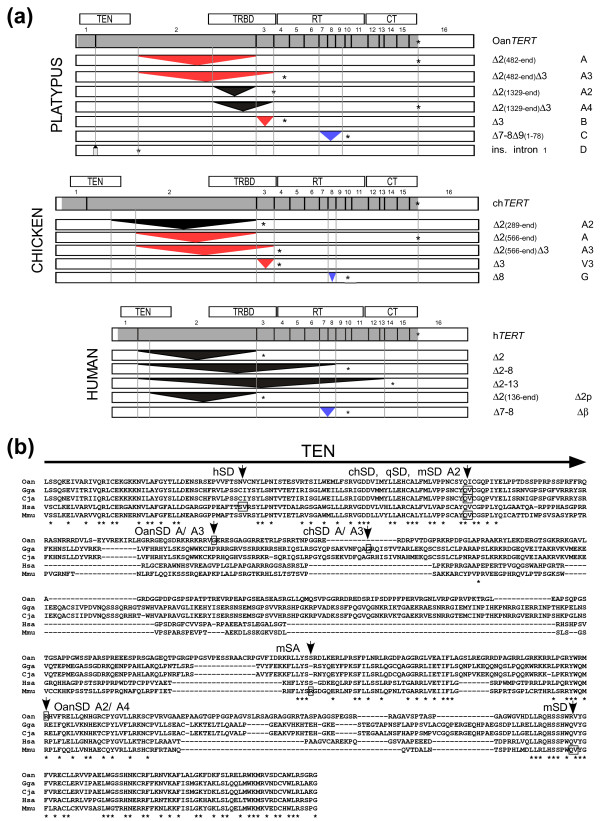
**Platypus alternatively spliced *TERT *variants.** (**a**) The comparison of platypus, chicken, and human AS variants. The figure show all identified platypus variants, and all chicken and human variants that involved the second exon. Additionally, the chicken and human variants most similar to platypus C variant are shown. The structure of wild-type (WT) platypus, chicken, and human TERT is shown at the top of each set of AS variants. The TERT ORFs are shaded. The positions of the exons and functional domains (TEN, TRBD, RT, CT) of TERT are indicated. The model of *TERT* transcripts with single alternative splice events are drawn to their relative sizes. Deletions (triangles) and insertion (gray rectangle) resulting from splicing events are indicated. The red triangles represent AS variants conserved between platypus and chicken. Blue triangle identifies AS *TERT* variant which contains a deletion of a similar RT region in platypus, chicken, and human *TERT*. The black triangles indicate AS variants which are not conserved among platypus, chicken, and human. Asterisks identify the location of the stop codons. The descriptive and trivial names of the *TERT* AS sequences are shown in the right margin. The numbers in the parentheses represent the position of the deletion in the exon which is indicated in front of these parentheses. The figure is based on data from the following GenBank entries of platypus isoforms: [GenBank:JF441069] (A), [GenBank:JF441066] (A3), [GenBank:JF441065] (A2), [GenBank:JF441068] (pA4), [GenBank:JF441070] (B), [GenBank:JF441067] (C), [GenBank:JF441072] (D); chicken isoforms: [GenBank:JF896279] (A2), [GenBank:DQ148473] (A), [GenBank:DQ681311] (A3), [GenBank:DQ256211] (V3), [GenBank:DQ148464] (G), and human isoforms: [GenBank:AB086379] (Δ7-8), and human isoforms [GenBank:JF896280] (Δ2), [GenBank:JF896283] (Δ2-8), [GenBank:JF896284] (Δ2-13). The available sequence of human Δ2p(136-end) variant is too short to be accepted by GenBank and is provided in the supplement (Additional file 3, p.5). (**b**) The ClustalX alignment of amino acid sequences encoded by the second exon of platypus (Oan), domestic chicken (Gga), Japanese quail (Cja), human (Hsa), and mouse (Mmu) TERT. The part of TEN domain present in the second exon is shown at the top. The novel SA and SD sites are indicated by small boxes. For mouse AS TERT variants see [GenBank:CF531121.1, GenBank:BC127068.1]. The prefixes to SD or SA represent species: Oan (platypus), ch (chicken), q (quail), h (human), and m (mouse). The name of the relevant platypus or chicken alternative splicing variant(s) is indicated after the SD and SA used to create this isoform(s). The residues shown by (*) are conserved in all species.

Seven alternatively spliced events were identified in platypus *TERT* that yield seven single AS forms (Figure 2a, see also Additional file 2: Figure S1a). Four of them (A, A2, A3, and A4) delete part of exon 2. The AS forms A and A3 use a novel splice donor (SD) located in the first quarter of exon 2. The AS variant A uses a splice acceptor (SA) site of the second intron and the AS variant A3 employs the SA of the third intron. Similarly, the AS variants A2 and A4 use a novel SD site located in the last third of the second exon in combination with a SA in the second intron (A2) or the third intron (A4). The additional AS variant, variant B, deletes the third exon and the sixth AS variant, variant C, has a deletion of the seventh, eighth and the part of the ninth exon. Finally, variant D was identified in a single clone from brain cDNA and contains an insertion cassette of 25 nucleotides derived from the first intron. The AS variants A and A4 retain ORF and AS variants A3, A2, B, C, and D contain premature termination codons (PTCs). The splicing events A to A4, and B are mutually exclusive, but each of them may be combined with C or D in one AS molecule.

The comparison of platypus AS forms with chicken and human isoforms revealed some similarities. AS forms involving the second exon have been described in human and chicken *TERT* ([58], unpublished data, and Figure 2). The chicken AS variant A is structurally similar to the platypus variant A. This form uses a novel SD site in second exon, located in close proximity to the platypus SD site in combination with a SA of the second intron and retains an ORF. Like the platypus A3, the chicken A3 variant containing a PTC uses this novel SD site in combination with a SA site of the third intron. In contrast, all human AS variants involving the second exon contain PTCs and only one of them uses a novel SD site while the three others employ a SD of the first intron. An additional similarity between chicken and platypus alternative splicing was revealed by the identification of the platypus AS variant B, which is identical to the chicken variant V3. The platypus variant C (Δ7-8, and part of 9 exon) has no precise analog among chicken or human AS variants, but chicken variant G (Δ8) and human AS variant β (Δ7-8) delete a similar region and all these forms contain PTCs. These results suggest that, although only seven AS variants of Oan*TERT* have been identified, they have strong structural similarity to several chicken AS variants including AS form A which retains an ORF.

### The low conservation of the second exon correlates with the evolution of novel splice sites

The second exon is the least conserved of all vertebrate *TERT* exons (Figure 1a). The conserved regions are at the beginning and the end of this exon that encode parts of the TEN and TRBD, respectively. These domains are separated by a linker with high sequence variability. In birds and mammals at least six novel SD and one SA sites evolved within the second exon (Figure 2b). In the sequence encoding the TEN domain there are two SD sites that create AS forms which contain PTCs. The first site (hSD) is involved in splicing of the human AS Δ2p(136-end) variant and the SD site (chSD, qSD, mSD) was identified in chicken and quail variant A2, as well as mouse AS TERT variants. Additional new SD and SA sites were identified in the second exon downstream from the sequence encoding the TEN domain. Interestingly, all novel SD sites involved in the production of the AS *TERT* variants that retain an original ORF are located downstream from the TEN domain. The two SD sites used by platypus and chicken AS *TERT* variants A (OanSD A/A3, chSD A/A3) are located in a sequence encoding the first hundred amino acids downstream from this domain. The SD site used by the platypus in-frame AS variant A4 (OanSD A2/A4) is also downstream from the TEN domain. This analysis suggests that the second exon, which has high sequence variability, was used to create the novel SD and SA sites during evolution. As a rule, all the AS *TERT* variants which retain the original ORF are downstream from the TEN domain, and potentially encode variants of TERT with the DNA-binding domain intact.

### The expression of alternatively spliced *TERT* variants in platypus tissues

**Figure 3:**
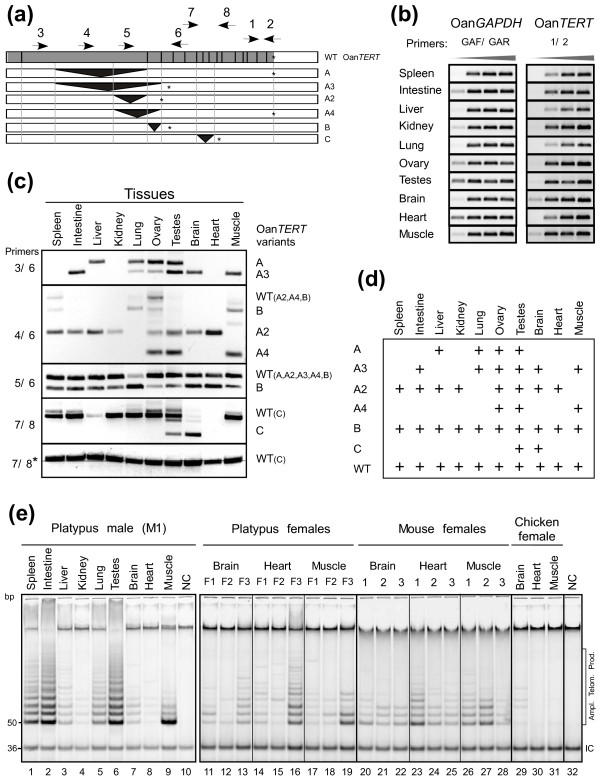
**Expression of *TERT and its AS variants in various tissues.*** (**a**) The schematic representation of the PCR strategy for detection of Oan*TERT* and its variants is shown. Arrows represent primers used for RT-PCR (Additional file 1: Table S1). The transcript spliced using 16 evolutionary conserved exons is designated as wild-type (WT) isoform. (**b**) The steady-state levels of *TERT* mRNA were determined by semiquantitative RT-PCR. The level of *GAPDH* served as a loading control. Aliquots from the PCR were taken every 5^th^ cycle beginning with cycle 35 for the amplification of *TERT* and cycle 30 for *GAPDH*. The PCR products were resolved on agarose gels and visualized with ethidium bromide. Gray triangles indicate an increasing number of PCR cycles. (**c**) The expression of Oan*TERT* AS variants was determined by RT-PCR (Additional file 1: Table S1). The position of PCR primers are shown in Figure 3a. The lower panel (7/8*) shows the PCR reaction with cDNAs obtained with synthesis using oligo-dT primer instead of random-hexamer priming. (**d**) The table summarizing the expression of Oan*TERT* AS variants is shown. (**e**) Telomerase activity in extracts from different tissues of a male platypus (M1; lanes 1–9) and from brain, heart and skeletal muscle of three platypus females (F1, F2 and F3; lanes 11–19) was determined by the TRAP assay. All three animals evaluated were obtained from wild populations and were sexually mature adults, however, their precise age is not known. Telomerase activity in the same tissues was determined in three mouse females and one chicken female (lanes 20–31). Extraction buffer serves as the negative control (NC) (lanes 10 and 32). Molecular weights are indicated in the left margin and the positions of the amplified telomerase products (6 bp ladder starting with 50 bp band) and a 36 bp PCR internal control (IC) are shown in the right margin.

The expression of *TERT* mRNA in a variety of platypus tissues was determined by RT-PCR using a set of eight specific primers (Figure 3a). Initially we determined the level of total *TERT* transcripts using primers that amplified sequences located between exons 14 and 16 which are not known to be involved in alternative splicing (primers 1/2, Figure 3b). Platypus *TERT* was expressed in all tissues tested: spleen, intestine, liver, kidney, lung, ovary, testes, brain, heart, and skeletal muscle. Surprisingly, the highest *TERT* mRNA expression was present in brain and skeletal muscle, the organs in which the expression of *TERT* is very low or absent in most of homeotherms [59]. 

The relative levels of the AS variants (with exception of rarely expressed variant D) in the various tissues was also determined (Figure 3c). First, the forward primers specific to different sequences in the second exon and the reverse primer specific to a sequence located in the fifth exon were employed (3/6, 4/6, 5/6). The expression of variant A with an original ORF was detected in liver, lung, ovary, and testes and the expression of the smaller A3 variant, a PTC-containing variant, in intestine, lung, ovary, testes, brain, and skeletal muscle. The A2 variant, containing a PTC, was detected in all tissues except lung and skeletal muscle. In contrast, the A4 variant with an original ORF was detected only in ovary, testes, and skeletal muscle. If the forward primer located within the sequence that is deleted in all the A variants (primer 5) was used in combination with reverse primer 6, then the Oan*TERT* was detected in all tissues. The products of this reaction represent part of wild-type (WT) *TERT* in combination with AS variant B in different relative ratios. Finally, additional PCR determined that the variant C was expressed in two tissues, in brain and testes (primers 7/8).

In summary, this analysis revealed that all variants were detected only in the testes (Figure 3d). The ovary expressed most variants except the rare variant C. Other tissues expressed different combinations of three to four AS *TERT* variants, suggesting that the alternative splicing of *TERT* is regulated during cell differentiation. In conclusion, the expression of *TERT* mRNA is high in most platypus tissues. Although, a significant number of *TERT* mRNAs are alternatively spliced, WT *TERT* transcripts were detected in all tissues suggesting that telomerase activity is present in these tissues.

### Telomerase activity is ubiquitous in platypus tissues

To investigate whether telomerase is active in most platypus organs as suggested by the TERT mRNA expression analysis, tissue extracts prepared from organs of an adult male platypus were analyzed by a telomerase repeat amplification protocol (TRAP) assay (Figure 3e, lanes 1–9). Telomerase activity was detected in all evaluated tissues, although at different levels. The highest levels of telomerase activity were detected in testes and intestine, following by spleen, lung, liver, skeletal muscle, and brain. The lowest levels of telomerase activity were detected in kidney and heart. In most homeotherms telomerase activity is very low or undetectable in heart, skeletal muscle, and brain [60-62]. Therefore, we determined telomerase activity in these tissues obtained from three different adult female platypuses and directly compared it to telomerase activity in these tissues obtained from female mice and chicken (lanes 11–31). Telomerase activity was detected in heart, skeletal muscle, and brain in all three platypuses, though variations among individuals were observed. Similar levels of telomerase activity were detected in mouse tissues (lanes 20–28). Individual variability was also present despite that two of the three females were from the same litter and all three animals were of the same age. In contrast, chicken heart and muscle did not expressed telomerase activity and very low levels were detected in chicken brain (lanes 29–31). These results show that telomerase activity is present in all nine analyzed platypus tissues. As in laboratory mice, telomerase activity was expressed also in tissues with low cell proliferation.

### Platypus telomere length

**Figure 4:**
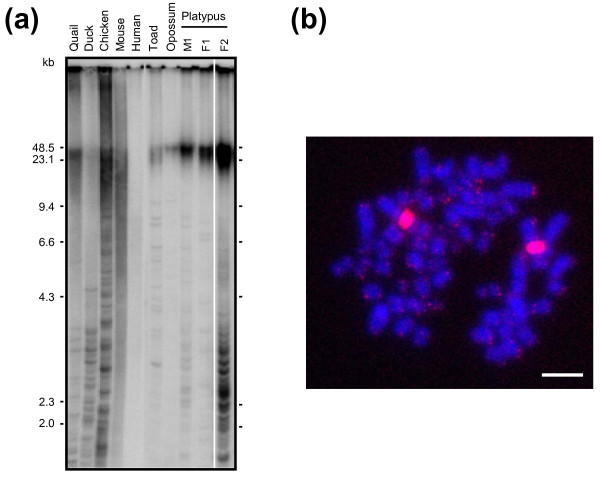
**Telomere length of the platypus. (a)** Telomere length was determined by terminal restriction fragment (TRF) length analysis as described in Material and Methods section. This analysis included DNA isolated from quail, duck and chicken embryos, human (BD Biosciences Clontech, Cat. No. S0950), spleen from 2 month-old male mice (strain C57BL/6), lung from toad *Xenopus tropicalis*, opossum *Monodelphis domestica*, and platypus (M1, F1 and F2). The amount of DNA analyzed for F2 was higher (5 μg) than for all other samples (3 μg). The molecular weights (kb) are indicated on both sides of the picture. **(b)** Fluorescence in situ hybridization with Cy3 primary conjugated telomere repeat (TTAGGG 42 mer) probe (red signal), on female platypus metaphase spreads. Platypus chromosome 1 has large interstitial telomeric repeats. Scale bar = 10 μm.

The average length of telomeres in mammals ranges from very long (up to 50 kb in some rodents and lagomorphs) to very short (below 10 kb in primates). However, species can be divided into two groups according to their telomere length, one group with the average telomere length below and the second above 20 kb [63]. Length of platypus telomeres was determined by telomere restriction fragment (TRF) analysis and their size was compared to the telomere length of other vertebrates (quail, duck, chicken, mouse, human, toad, and opossum) (Figure 4a). Platypus telomeres were above 20 kb (approximately 30–40 kb long) similarly to quail, chicken, mouse, toad and opossum. In contrast, human and duck had much shorter telomeres (4–10 kb). In conclusion, platypus belongs to the group of animals with long telomeres. 

In terms of physical distribution of telomere repeats, frequent interstitial telomere sequences (ITS) have been reported in birds, frogs, and marsupials [64-67]. The ITS (discontinuous bands of molecular weights below 10 kb) were visible in all birds, toad and platypus, but they were undetectable or absent in human and mouse tissues. The FISH analysis confirmed the presence of end-terminal telomeric repeats and revealed an extensive ITS on chromosome 1 in platypus genome (Figure 4b). The strong ITS could possibly interfere with the detection of telomeric sequences at the end of chromosomes. However, the strength of this signal is much greater than signals at the ends of chromosomes on the regular FISH analyses so if this sequence is formed from uninterrupted telomeric repeats then it would not appear on the gel because its high molecular weight. We rather expect that this ITS is interspersed by nontelomeric sequences containing sites for frequently cutting restriction enzymes and appear in the gel as fragments below 4 kb like the ITS of birds [68].

### Chromosomal localization of platypus *TERT*

**Figure 5:**
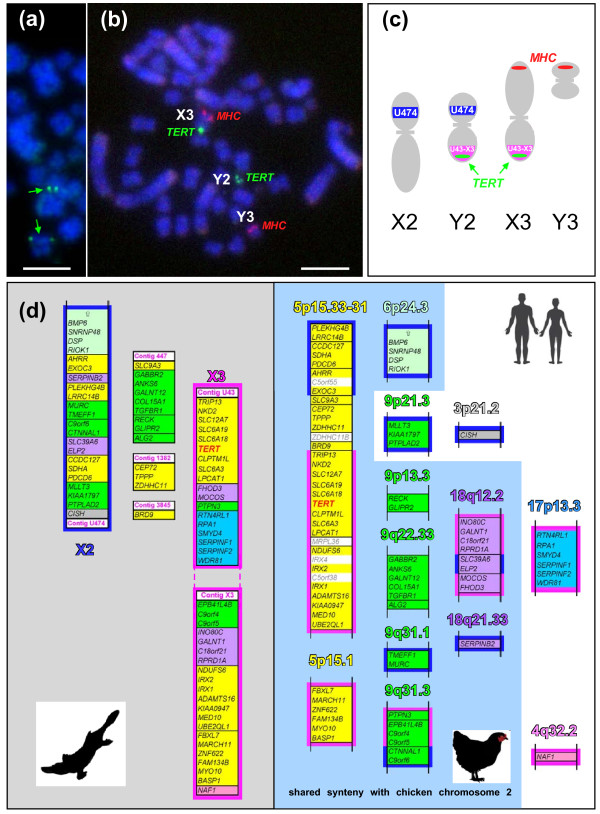
**Chromosomal localization of the Oan*TERT *locus**. (**a**) Fluorescence in situ hybridization (FISH) of *TERT*-containing BAC clone on male platypus metaphase spread. Green fluorescence signals indicate the position of the *TERT* locus on a pair of metacentric chromosomes (green arrows). Scale bar = 10 μm. (**b**) FISH of *TERT* (green) and the major histocompatibility complex (*MHC*) (red) was performed as described in (a). *TERT* signals were identified on the pseudoautosomal regions of Y2qX3p. (**c**) Schematic representation of platypus chromosomes X2, Y2, X3, Y3. Approximate positions of *TERT* and *MHC* signals are indicated. Locations of gene contigs Ultra 474 (U474, blue), Ultra 43 and X3 (U43-X3, purple) are indicated as determined in this (c) and previous works [54,55]. (**d**) The analysis of shared synteny between *TERT*-containing chromosomal regions of platypus and human. The platypus genes located at platypus X2 and X3 chromosomes and their human orthologs (on human chromosomes) are indicated by distinct colored frames. The genes without the colored frame were not yet localized to platypus chromosomes. The numbers of the platypus gene contigs as present in current Ensembl database are indicated. “Ultra” in the designation of some contigs is shortened to “U”. The mutual orientation of platypus X3 and U43 contigs and their relative position on the X3 chromosome were not established so that the orientation shown in the panel C may not reflect their actual positioning and orientation. The genes located at different human chromosomes and their platypus orthologs (on platypus chromosomes) are indicated by distinct colored backgrounds. Light blue field identifies parts of human chromosomes that share synteny with chicken chromosome 2. Human genes located on 5p15 which have no platypus orthologs are indicated by gray lettering. The vertical arrows in the group of 6p24.3 genes and their platypus X2 orthologs represents a group of approximately 25 additional orthologous genes that extend shared synteny between 6p and X2.

The platypus physical map is relatively sparse and only 15% of known genes have been mapped to chromosomes [52]. Additional mapping primarily concentrated on the gene content of the platypus sex chromosomes and platypus orthologs of the human X chromosomal genes [54,55,69]. While the location of the *TERT* locus-containing contig has not been established in these mapping projects, preliminary synteny analysis of *TERT*-containing chromosomes (human chromosome 5 and chicken chromosome 2) with platypus chromosomes suggested the possibility that Oan*TERT* may map to the platypus sex chromosomes. In fact, initial FISH hybridization employing the BAC clone 606P3 localized Oan*TERT* on a pair of small metacentric chromosomes of differing sizes (Figure 5a). Co-hybridization with BAC clone 462c1, which contains the major histocompatibility complex (*MHC*) class I and class II genes that were previously localized to X3q/Y3 [69], established that these chromosomes are X3 and Y2 and that Oan*TERT* is located in the pseudoautosomal region of X3p/Y2q (Figure 5 b,c). 

The physical mapping of Oan*TERT* contig genes extended previously described homology between human chromosome 5, chicken chromosome 2 and platypus X2/Y2/X3 [54,55]. Detailed synteny analysis of the genes surrounding platypus and human *TERT* shows that the entire section of human chromosome 5 that shares synteny with chicken chromosome 2 (the terminal region of 31.5 MB shown in Additional file 6: Table S2) contains 70 protein-coding genes. Orthologs of 61 of these genes were found in the platypus genome and 90% of these are now mapped to chromosomes. Almost all of the mapped genes are located on the short arms of platypus sex chromosomes X2 (7 genes) and X3 (46 genes). Only one gene (*SRD5A1* ortholog) is located elsewhere, on sex chromosome X1.

The short arms of platypus X2 and X3 chromosomes that contain orthologs of human genes located at human chromosome 5 (listed in Table S2, Additional file 6) also contain orthologs of human genes located on human chromosomes 3, 4, 6, 9, 17, and 18 (Figure 5d). A majority of these genes (as well as 5p-orthologs) are part of one ancient linkage group conserved on chicken chromosome 2 (approximately between 55 and 95 MB). Only a few X2p/X3p-genes do not belong to this group, including the orthologs of *MLLT3PTPLAD2* group, *CISH*, and *NAF1*[54]. One additional exception, identified in this work, is a group of six genes with human orthologs located at 17p13 (sharing synteny with chicken genes localized on chromosome 19). Incorporation of these genes into a linkage with genes which orthologs map to chicken chromosome 2 appears to be monotreme-specific.

### Sex differences in telomerase expression

**Figure 6:**
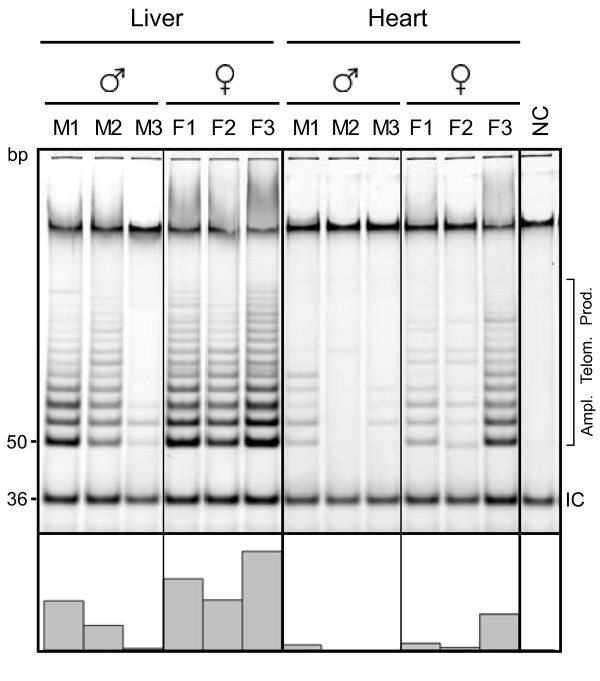
**Telomerase activity in extracts from liver and heart of three platypus males and females.** Telomerase activity was determined by the TRAP assay as described in Figure 3e. The column graphs under the lanes indicate the relative abundance of PCR-amplified telomerase reaction products determined by quantification of fluorescent signal.

The localization of the Oan*TERT* gene on sex chromosomes and difference in telomerase activity between male and female heart tissues (Figure 3e) prompted us to analyze telomerase activity in male and female platypus tissues. Telomerase expression in liver and heart was analyzed since differences in telomerase expression or telomere length in these tissues have been described in the rat [70,71]. Material was obtained from adult platypuses caught from actively breeding wild populations, but their precise age is not known. However, results presented in Figure 3e demonstrated that individual variability in telomerase activity in these platypuses is not greater than individual variability in inbred mice at adulthood. We determined the levels of telomerase activity in tissues from three male and female animals (Figure 6). Despite variations between individuals, the results clearly demonstrated that on average females expressed higher levels of telomerase activity in both tissues than males. 

## Discussion

### Platypus *TERT* and its position in the evolution of vertebrate *TERT* genes

Analysis of the platypus *TERT* gene creates an important framework for the evolutionary comparison of *TERT* genes of eutherian mammals and sauropsid reptiles (including birds). Platypus, one of a few surviving species of monotremes, has a unique evolutionary position. Monotremes, followed by marsupials, first split from all other contemporary mammals after the divergence of the synapsid lineage leading to mammals and the sauropsid lineage leading to contemporary reptiles and birds [52]. Monotremes and marsupials, therefore, might retain some features of the pre-mammalian synapsid *TERT* gene that were lost in eutherian mammals. The higher levels of sequence similarity of four principal TERT domain of OanTERT to the marsupial, and avian TERT than to the eutherian TERT support this hypothesis. Moreover, platypus and marsupials also retain three extended linker regions of a non-conserved sequence in the N-terminal region of the TERT protein which are about twice longer than the linkers in eutherians and their size matches more closely the linkers in sauropsids.

Interestingly, the long and intermediate linker regions present in most of the tetrapods except the eutherian mammals were not found in the well-studied group of ray-finned fish but are present in elephant shark TERT. Elephant shark belongs to the oldest evolutionary group of contemporary jawed vertebrates [72]. It is, therefore, possible that the long TERT of the shark may represent the original jawed-vertebrate TERT structure and that the linkers were shortened independently in ray-finned fish and eutherian mammals. Divergent evolution of fish TERT is supported by the evolutionary tree built mostly on the conserved TERT sequences outside the linker regions that situates the elephant shark TERT closer to the tetrapod TERT and farther from ray-finned fish TERT (Figure 1d). These changes could be, at least partly, a consequence of genome duplication and increase in mutation rate in the teleost branch of ray-finned fish [72-75]. The long TERT structure described in sea urchin suggests an even deeper evolutionary root of the extended linker [37].

The long-term evolutionary stability of the extended linker regions suggests a possible function that constrains evolutionary change. The invertebrate sea urchin has two TERTs with extremely long linker regions and a specific function in embryogenesis were suggested for these structures [37]. In vertebrate the presence of the longer linker region in TERT proteins of different species correlates with evolutionary distribution of interstitial telomeric sequences (ITS). ITS were detected in the platypus genome and while these repeats were described in most vertebrate species they occur at a higher frequency in frogs and birds but not eutherian mammals [64,65,67]. Several mechanisms contribute to the formation of ITS depending on their length and sequence organization [76,77]. For the formation of most of the short ITS the direct involvement of telomerase during the repair of double-stranded breaks has been implicated. Therefore, one of possible functions of the extended linker region of the TERT protein might be to increase the recruitment of telomerase to sites of double-strand breaks. The precise function of the extended linker of TERT however remains to be established.

The shortening of the once extended TERT molecules could be also restricted by the necessity to adapt to interacting molecules. One of such molecule could be the telomerase RNA (TR) because the three TERT linkers are either inside or close to the TR-binding domain. The minimalist size of the TR that developed in ray-finned fish correlates with an extensive reduction of the linkers in the fish TERT protein [78]. Further, the largest vertebrate TR has been described in sharks and the predicted size of the elephant shark TERT is also the largest of all vertebrates [79]. However, the TERT-TR size correlation does not hold in birds. Birds encode a large TERT protein but the TR is of a similar size as that of mammals [79].

### The evolution of AS variants

Alternative splicing plays an important role in creating proteome diversity in multicellular animals [80]. More than 95% of human intron-containing genes express one or several alternatively spliced variants. While the alternative splicing of *TERT* transcripts has not been described in protists, it occurs extensively in multicellular organisms such as plants and Metazoa (for recent review see [13]). All vertebrate *TERT* transcripts are currently believed to be alternatively spliced, however, there are great differences in frequency and structure of AS variants [13]. The largest number of AS *TERT* variants have been identified in chicken and human cells, 37 and 21, respectively ([58,81-85] and unpublished data). In fish, amphibians and rodents only a few variants are known [13]. In contrast to plants, almost all the AS *TERT* variants differ in structure between mammals and birds. There is only one known example of AS variants which is structurally identical, an in-frame deletion of exon 10 in the chicken and rat [58,86]. We identified seven AS variants of Oan*TERT* which likely represent all the major platypus *TERT* AS variants. The variant B is identical to chicken variant V3 and surprisingly, the structure of three other variants (A, A3, C), also resembles the chicken AS isoforms. This unprecedented conservation of AS variants between a basal mammal lineage and a bird suggests an important function for alternative splicing in *TERT* regulation. It also suggests that the repertoire of AS *TERT* variants identified in eutherian mammals evolved recently.

Two AS sites evolved in the second exon of Oan*TERT* which yield four AS variants, two of which retain an ORF. In contrast to variants containing PTCs, which are targeted by nonsense mediated decay (NMD), these AS variants will likely produce TERT proteins [87]. The A variant, which maintains an original ORF, is highly expressed in liver, lung, ovary, and testes, like the corresponding chicken variant A. Interestingly, the chicken and platypus A variants, as well as AS variant A4, have maintained the TEN domain which plays a role in DNA-binding, however, based on their structure, they would not possess telomerase activity [88,89]. Several telomerase activity-independent functions have been attributed to the TERT protein including the enhancement of cell proliferation. The alternatively spliced protein-coding *TERT* isoforms might provide some of these functions [6,7].

### The tissue-specific expression of *TERT*

Telomerase activity is downregulated during development in most somatic cells simultaneously with a reduction in their rate of proliferation and subsequent differentiation [10]. This process is accompanied by the transcriptional downregulation of *TERT*[41]. However, the levels of repression differ between cold-blooded (fish and amphibian) and warm-blooded species (mammals and birds). In adult organs of many mammals and birds, the expression of *TERT* is repressed, with very low or undetectable levels expressed in heart, skeletal muscle and brain [11,41,62,90]. In contrast, the expression of *TERT* in all adult organs of fish remains at high levels [40,42,48]. Likewise, telomerase activity is widespread in the tissues of the toad *Xenopus tropicalis* including heart, brain, and muscle [91]. However, there are significant differences even among mammals [63,92]. Most mammals with telomeres longer than 20 kb, including small rodents, bats, some felines, lagomorphs, elephant shrews, and the Virginia opossum, express significant levels of telomerase in adult somatic cells. The platypus, with telomeres longer than 20 kb and telomerase activity in all adult tissues, belongs to these species. Telomerase activity in the platypus was even detected in skeletal muscle, brain, and heart, though in lower levels than in other organs with higher cell proliferation rates. The pattern of telomerase expression in platypus, as well as the length of its telomeres, correlates well with its relatively small size (1–2 kg), supporting the hypothesis that telomerase activity and telomere length inversely correlate with body mass in mammals [63].

### The location of Oan*TERT* on the X3/Y2 sex chromosomes

The chromosomal position of platypus *TERT* is, to our knowledge, the first instance among amniote vertebrates to localize *TERT* on sex chromosomes. The homology of platypus and echidna Y2/X3 chromosomes suggests that this is common in monotremes [55]. *TERT* genes of the other amniotes analyzed to date are located on autosomes [43,93]. Platypus sex chromosomes have large regions orthologous to avian ZW chromosomes while they generally lack orthologs of genes located at therian XY sex chromosomes [53-55]. Interestingly, the region on human autosome 5 containing the *TERT* gene is located in proximity to a region homologous to a part of the avian sex chromosome Z [54]. Further, the inspection of the Ensembl database shows that similar juxtaposition is also found in other mammals, including macaque, marmoset, mouse, cow, pig, horse, and opossum. These observations lead to an interesting possibility that the last common ancestor of all mammals might have the *TERT* gene chromosomal region associated with an avian-type Z chromosome. The *TERT* position on sex chromosomes may not be an evolutionary innovation of monotremes, but one of the features of mammalian ancestors preserved in the platypus genome and only lost in therian mammals as a result of the evolution of the new XY sex chromosomes [94].

Comparison of the genes surrounding the *TERT* locus in platypus, human, and chicken (Figure 5c and Additional file 6: Table S2) suggests that the formation of a discontinuous sex chromosome chain in platypus lead to the relocation of some genes to different chromosomes. The genes found in one region in human chromosome 5 and chicken chromosome 2 are divided into three X chromosomes (X1, X2, and X3) in platypus. Similarly, chicken Z-homologous genes are located on four platypus X chromosomes (X1, X2, X3, and X5) [55]. This suggests that the evolution of the sex chromosome chain might have created instability and possibly increased the rate of interchromosomal rearrangements of the chromosomes involved [95,96].

The Oan*TERT* locus is located in a pseudoautosomal region of the platypus sex chromosomes. This location, however, does not automatically guarantee identical regulation of the X and Y copies of *TERT*. Different spacing of two BAC clones (165 F5, 151 O20) hybridizing spots at Y2q and X3p might indicate different chromatin folding and epigenetic state of these two chromosome arms or alternatively incomplete homology [54]. These differences may lead to differential expression of the two *TERT* copies.

### Gender-dependent telomerase expression

Gender-specific differences between telomere length in several mammalian species are well established. Female mice and rats have significantly longer telomeres than age-matched males [71,97]. In human, female telomeres are longer than male, with the exception of the Amish people [98-100]. These differences in telomere length positively correlate with life expectancy [101,102]. Telomere length is the result of a complex regulation involving both the synthesis and attrition of telomeric repeats [103,104]. Telomerase activity is responsible for telomere synthesis and generally correlates with telomere length. Therefore, telomerase activity likely contributes to gender-specific differences in telomere length and, in turn, life expectancy. However, very limited information is available concerning gender-specific telomerase activity in mammals. Cardiac myocytes obtained from female rats express significantly higher levels of telomerase than male cells [70]. Our results demonstrating that female platypuses express higher telomerase activity than males in at least two tissues, liver and heart, extend this observation. Telomerase activity in these tissues may also correlate with lifespan. While this issue will require further study, there are indications that the lifespan of the female platypus in the wild is greater than that of the male. Studies performed over a 30 year period in the upper Shoalhaven River demonstrated that the recapture rate is significantly biased toward females [105]. In addition, the oldest female captured in these studies was 21 years old, while the oldest male was 7.

Recently, it has been shown that there are gender differences in telomere length in the kakapo, *Strigops habroptilus*, a parrot endemic to New Zealand [106]. The male kakapos have longer telomeres than females which is in distinct contrast to mammals. Previously, it has been suggested that estrogen in mammals is at least partly responsible for the increased telomere length in females since estrogen is a positive regulator of *TERT* transcription [107]. Female birds, however, also express high levels of estrogen like their mammalian counterparts, therefore, this mechanism cannot apply to birds [108,109]. Moreover, in contrast to human, where paternal inheritance of telomere length to daughters and sons have been described, the kakapo telomere length is inherited maternally to sons [110,111]. To reconciliate these opposing patterns of regulation and inheritance of telomere length it has been proposed that heterogametic sex (either XY in mammals or ZW in birds) chromosomes X and Z have a central role in determination of gender-specific differences in telomere length.

The chromosomal localization of *TERT* on platypus sex chromosomes together with gender-specific expression supports this hypothesis and suggests that gender-specific differences in telomere length are related to the original localization of the *TERT* gene on sex chromosomes. One possible scenario is that the *TERT* activator was located on the sex chromosome of a common ancestor of mammals and birds. This localization might be retained in monotremes with the hypothetical *TERT* activator located on a X specific part of the X chromosome which in platypus may be only partially dosage-compensated [112]. The large gender-specific differences in telomerase expression as well as the lifespan of platypuses may be the result of concomitant localization of *TERT* and its activator on sex chromosomes. A large part of the platypus sex chromosomes is orthologous to sex chromosome Z in birds, which also does not involved a chromosome-wide dosage compensation mechanism [54,113,114]. The localization of a *TERT* activator on the avian Z chromosome could result in higher levels of telomerase activity in male than female birds because the expression of genes localized on bird sex chromosomes is also weakly dosage-compensated. Finally, therian mammals developed a new sex chromosome system and evolved additional levels of regulation beyond the already existing regulatory circuits [115]. In case of *TERT* gender-specific regulation this relationship could remain conserved in therian mammals while subjected to a level of additional control of the new evolved X chromosome.

## Conclusions

The characterization of platypus TERT and its comparison with other vertebrate TERT proteins revealed that it shares many features with birds and other reptiles suggesting that it represents the ancestral mammalian TERT. Structural and regulatory features specific to TERT of eutherian mammals have, therefore, evolved more recently after the divergence of monotreme mammals. Additionally, the results suggest possible relationship between the chromosomal localization of *TERT* and gender-specific expression of telomerase.

## Methods

### GenBank accessions

The sequences of platypus *TERT* cDNA and alternatively spliced *TERT* variants were submitted to GenBank under accession numbers [GenBank:JF441065, GenBank:JF441066, GenBank:JF441067, GenBank:JF441068, GenBank:JF441069, GenBank:JF441070, GenBank:JF441071, GenBank:JF441072]. The platypus *TERT* cDNA sequence is a composite of overlapping cDNA clones (Additional file 2: Figure S1a). Due to the extensive *TERT* alternative splicing which occurs in platypus tissues, secondary structures, and limited tissue resources we were not able to isolate a contiguous *TERT* clone.

### Animals, cell lines, and tissue culture

Material used in this study was obtained from adult wild male and female platypuses (The University of Adelaide Animal Ethics Committee permit no. S-49-2006 to F.G.). Quail (*Coturnix japonica*) embryonated eggs were obtained from University of Texas, Austin. Embryonated duck (*Anas platyrhynchos*) eggs (Khaki Campbell) were obtained from McMurray hatchery (Webster City, IA). Embryonated eggs from pathogen-free White Leghorn chickens (*Gallus gallus*) (the SPF-SC strain) were obtained from Charles River SPAFAS (North Franklin, CT). Mice (*Mus musculus*) (BALB/c, 2 month-old females) were from University of Texas, Austin and opossum tissues (*Monodelphis domestica*) (9 week-old male) from the University of New Mexico, Albuquerque.

### RNA isolation

Total RNA was extracted from frozen tissues using TRIzol reagent (Invitrogen, Carlsbad, CA), according to the manufacturer’s instructions.

### Isolation of platypus *TERT* and its AS variants

The platypus *TERT* clones were RT-PCR amplified from cDNA obtained from platypus brain testes, ovary, heart, and muscle using 15 distinct combinations of primers Table S1c, Additional file 1). The PCR strategy is shown in Figure S1a (Additional file 2). The amplified fragments were cloned into pGEM-T Easy (Promega, Madison, WI) or into pCR2.1-TOPO (Invitrogen) vectors. Forty four clones were isolated and sequenced.

### Sequence analysis

The TERT protein sequences from species other than platypus were obtained from different sources. Most of the TERT sequences were obtained from annotated GenBank files: *Homo sapiens* [GenBank:NP_937983.2], *Macaca mulatta* [GenBank:NP_001177896.1], *Canis lupus* [GenBank:NP_001026800.1], *Bos taurus* [GenBank:NP_001039707.1], *Mus musculus* [GenBank:NP_033380.1], *Rattus norvegicus* [GenBank:NP_445875.1], *Mesocricetus auratus* [GenBank:AAF17334.1], *Gallus gallus* [GenBank:NP_001026178.1], *Coturnix japonica* [GenBank:ABG75863.1], *Cairina moschata* [GenBank:ABO65149.1], *Xenopus laevis* [GenBank:NP_001079102.1], *Danio rerio* [GenBank:NP_001077335.1], *Nothobranchius furzeri* [GenBank:ACN38321.1], *Oryzias latipes* [GenBank:NP_001098286.1], *Oryzias melastigma* [GenBank:ABB92622.1], *Epinephelus coioides* [GenBank:ABC47309.1], *Takifugu rubripes* [GenBank:AAX59693.1], *Strongylocentrotus purpuratus* [GenBank:NP_001165522.1, GenBank:NP_001123288.2], *Daphnia pulex* [GenBank:EFX76361]. Two TERT sequences from GenBank were predictions by automatic computational analysis (*Pan troglodytes* [GenBank:XP_001141571.1], *Monodelphis domestica* [GenBank:XP_001369432.1]). Three model TERT sequences were obtained from the Ensembl database (http://www.ensembl.org), specifically TERT protein sequences of *Loxodonta africana* [Ensembl:ENSLAFP00000010943], *Taeniopygia guttata* [Ensembl:ENSTGUP00000008676], and *Anolis carolinensis* [Ensembl:ENSACAP00000001407]. The two predicted sequences from GenBank and all three protein models from Ensembl were checked for accuracy by alignments to genomic sequences as well as by multiple protein alignment and small scale corrections were made when necessary (for details see the Additional file 3, p. 1–2). Four TERT sequences were assembled *de novo* from the genomic data available in GenBank. Two complete assembled sequences (*Hydra magnipapillata, Helobdella robusta*) were submitted to GenBank under accession numbers [GenBank:BK008019, GenBank:BK008020]. The gapped, incomplete sequences (*Macropus eugeniiCallorhinchus milii*) are provided in the supplement (Additional file 3, p. 3–4). Protein sequence alignments were constructed by the ClustalX program [116]. The alignment of all sequences is shown in Figure S3 (Additional file 5, PDF version) and in Additional file 7 (text version). The boundaries of the protein domains and motifs were determined based on previously described vertebrate, invertebrate and protist TERT proteins [38,44,88,89,117-120]. The boundaries are shown in the Figure S2 (Additional file 4). The number of similar amino acids was determined using GeneDoc computer program (http://www.psc.edu/biomed/genedoc). Amino acid similarity is calculated based on BLOSUM62 matrix (BLOSUM35 matrix gave very similar results - data not shown). The percentage is shown relative to the number of residues in OanTERT or in OanTERT functional domains.

The evolutionary tree was constructed by Bayesian inference phylogenetic method. First, the protein sequences were aligned by ClustalX (Additional file 5: Figure S3). After deletion of sequences from the alignment that were not used for building of the tree, all aligned columns containing either gap or unidentified amino acid (X symbol) were removed using Gapstreeze tool software by setting the gap tolerance to 0% (Los Alamos HIV Sequence Database; http://www.hiv.lanl.gov/content/sequence/GAPSTREEZE/gap.html). The resulting alignment consisted of 618 columns of aligned amino acids. Bayesian analysis was performed by MrBayes 3.2.1 program [121]. Evolutionary models implemented by the program included equal rate variation across sites and the fixed-rate amino acid substitution model (aamodelpr = fixed(jones)). In preliminary tests, this fixed-rate amino acid substitution model was repeatedly preferred by the program for this set of protein sequences when mixed amino acid model was applied. Two Bayesian analyses each consisting of four Metropolis-coupled Markov chain Monte Carlo were run in parallel for 250,000 generations and sampled every 100^th^ generation. Convergence of both analyses was assessed using a plot of the generations versus the log probability of the data. The consensus tree was created with burn-in value set to 625. The tree was plotted by the tree-drawing program Dendroscope [122].

### Synteny analysis

The information about platypus, human, and chicken genes used in synteny analysis (Figure 5d and Additional file 6: Table S2) was retrieved from the latest Ensembl database (Ensembl genes 63). In most cases, the orthology of platypus genes with human and chicken genes corresponded to that assigned by the Ensembl database. For two human genes, *GABBR2* and *PAPD7*, we have assigned orthology to platypus genes based on their position within the same contiguous region in both species and based on high similarity of their protein products established by BlastP program.

### Tissue specific expression of platypus *TERT* by RT-PCR

Total RNA from platypus tissues (4 μg) together with 1 μl of random-hexamer primers were denatured for 10 min at 80°C in 11 μl of water. First strand cDNA synthesis was carried out with 15 U of ThermoScript RT (Invitrogen), 2 μl of 10 mM dNTP, 1 μl of RNaseOUT and 2 μl of 100 mM dithiothreitol at 50°C for 1 hour. The reaction was stopped by 5 min incubation at 85°C. The RNA was destroyed by RNase H treatment and the reaction was diluted with 20 μl of water. For detection of *TERT*, its AS variants, and *GAPDH* by RT-PCR, 2 μl of the first-strand synthesis reaction was amplified by 2.5 U Herculase Hotstart DNA polymerase (Agilent, Santa Clara, CA) with appropriate primers (Additional file 1: Table S1).

### Telomerase Repeat Amplification Protocol (TRAP)

The level of telomerase activity in various tissues was evaluated using the TRAP assay [58]. Whole cell extracts were prepared with CHAPS buffer [11]. Equivalent amounts of protein extracts (20 μg of total protein) were first incubated with 0.1 μg of the unlabeled TS primer and all four dNTPs (50 μM each) in TRAP reaction buffer, 0.8 mM spermidine, and 5 mM β-mercaptoethanol in a total reaction volume of 50 μl for 45 min at 37°C. All oligonucleotides - TS, ACX, NT, TSNT were described previously [123]. The reaction was stopped by incubation at 94°C for 2 min. Aliquots of synthesis (2.5 μl) were then PCR amplified in TRAP buffer with all four dNTPs (50 μM each), 0.1 μg Cy5-labeled-TS primer, 0.1 μg ACX primer, TSNT primer mix, and 1 μl of Advantage cDNA Polymerase mixture (BD Biosciences Clontech, Mountain View, CA) in 50 μl of total reaction volume. TS primer labeled at the 5′ end with Cy5 was obtained from IDT (Coralville, IO). PCR amplification started with 94°C for 2 min followed with 36 cycles (30 sec at 94°C, 30 sec at 55°C, and 1 min at 72°C). All telomere repeat synthesis and PCR amplification reaction mixtures were supplemented with acetylated BSA to a final concentration 0.5 mg/ml (New England Biolabs, Ipswich, MA). The TRAP PCR products (1/3 of the total reaction per well) were separated on 7.5% acrylamide gels (ratio of acrylamide to bis-acrylamide 19:1). Gel images were captured using a Typhoon Trio imager in fluorescence mode (GE Healthcare, Waukesha, WI). Quantification was performed by Quantity One 1-D analysis software (Bio-Rad, Hercules, CA).

### Terminal restriction fragment (TRF) length analysis

TRF analysis was performed as described previously [58]. High-molecular-weight genomic DNA (3 μg) was digested with a cocktail of restriction enzymes (HinfI, AluI, HhaI, HaeIII, MspI, and RsaI) and separated in a 0.6% TBE agarose gel. Undigested and HindIII digested λ phage DNA was used as marker. DNA was Southern transferred to a Hybond-N+ membrane (GE Healthcare, Waukesha, WI) and hybridized at 42°C in Ultrahyb solution (Ambion, Austin, TX) to the telomeric probe (CCCTAA)_6_ end-labeled with [γ-^32^P]-ATP. Blots were washed under stringent conditions. Subsequently, the blots were rehybridized with a λ probe to visualize the position of the markers.

### Fluorescence *in situ* hybridization of BAC clones

Standard fluorescence *in situ* hybridization (FISH) protocol on metaphase chromosomes derived from fibroblast cell lines was followed as described previously [123]. Briefly, slides containing spread metaphase cells were treated with RNase (100 μg/ml/SSC, 30 min) and pepsin (10% pepsin in 0.01 M HCl, 10 min), and fixed in 1% formaldehyde/PBS/50 mM MgCl_2_. Slides where dehydrated in ethanol series followed by denaturation at 70°C with 70% (v/v) formamide/2 × SSC. Slides were dehydrated in an ethanol series and hybridized at 37°C overnight. The slides were washed in 50% formamide/2 × SSC (42°C), 2 × SSC (42°C) and 0. 1 × SSC (60°C) and stained with DAPI solution (10 μl DAPI (1 μg/ml) in 50 ml 2 × SSC) and mounted with vectashield solution (Vector Labs, Burlingame, CA). The platypus BAC clones CH236-606P3 (TERT) and OA_B_462c1 [GenBank:EU030443] (MHC) used for hybridization were obtained from CHORI (Oakland, CA). The clone OA_B_462c1 was previously mapped to X3Y3 [69]. To investigate telomere repeats, a Cy3 primary conjugated telomere repeat (TTAGGG 42-mer) probe was used (Geneworks, SA, Australia).

## Competing interests

The author(s) declare that they have no competing interests.

## Authors’ contributions

RH and JN carried out cloning, sequence characterization, RT-PCR, TRAP, TRF and synteny analysis, SLL prepared platypus RNA and tissue samples and performed FISH hybridization. FG and HRB supervised the work carried out in their laboratories and edited the manuscript. RH and JN conceived the study and wrote manuscript. All authors read and approved the final manuscript.

## Supplementary Material

Additional file 1 **Table S1.** PCR methods (PDF). (a) Primers used for identification of Oan*TERT* and Oan*GAPDH* expression. (b) PCR conditions. (c) Primers used for cloning of Oan. Click here for file

Additional file 2 **Figure S1.** Platypus *TERT* cDNA clone (PDF). (a) Cloning strategy. (b) The sequence comparison of the genomic contig with the platypus *TERT* cDNA. (c) The assembled sequence of Oan*TERT*. (d) The region of genomic sequence flanking at 3′ side the sequence matching Oan*TERT* cDNA. (e) The region of genomic sequence flanking at 5′ side the sequence matching Oan*TERT* cDNA. Click here for file

Additional file 3 **Sequences not available from sequence databases (PDF).** All these sequences, except Δ2p(136-end), can also be retrieved in text format from the Additional file 6. (a) Corrections of TERT protein sequence models. (b) *De novo* generated TERT protein sequence models. (c) Human AS *TERT* variant Δp2(136-end). Click here for file

Additional file 4 **Figure S2.** Sequence alignment of platypus TERT with proteins of seven selected vertebrate species (PDF). The boundaries of the different domains and sequence motifs are indicated. This alignment serves as basis for the schematic representation of TERT proteins in Figure 1a. Click here for file

Additional file 5 **Figure S3.** Sequence alignment of vertebrate TERT proteins (PDF). The ClustalX sequence alignment of TERT proteins used for the construction of the tree shown in the Figure 1d. Click here for file

Additional file 6 **Table S2.** Synteny analysis (PDF). Conserved synteny among human chromosome 5, platypus chromosomes X2 and X3 and chicken chromosome 2. Click here for file

Additional file 7 **Alignment (TXT).** The alignment presented in Figure S3 (Additional file 5) provided in text format. Click here for file
