# Supplementary material for: Insights into the evolution of mammalian telomerase: Platypus TERT shares similarities with genes of birds and other reptiles and localizes on sex chromosomes
Source: BMC Genomics. 2012 Jun 1;13:216. doi: 10.1186/1471-2164-13-216 (PMC3546421; doi:10.1186/1471-2164-13-216)
Supplement: Additional file 1 — Table S1. PCR methods (PDF). (a) Primers used for identification of OanTERT and OanGAPDH expression. (b) PCR conditions. (c) Primers used for cloning of Oan. [file 1471-2164-13-216-S1.pdf]

TABLE S1a. Primers used for identification of Oan*TERT* and Oan*GAPDH* expression

Name[a]	Primer	Gene	Position[b]
1	CTGCGTCCTCCGGCTTCCATTTAATCAGC	Oan*TERT*	14/3536/F
2	GTCGCAGCTTCTAGCATCAGCATCGTAGG	Oan*TERT*	16/3822/R
3	GGACTCCTCCCCTCCCCGCCCCTCCTCCCC	Oan*TERT*	2/554/F
4	AAGCGGCGGCTCCCCGGACGCTACTGGCGG	Oan*TERT*	2/1524/F
5	GCCGCTTCCTCAGGAACGTCAAGGCCTTCTTGGCCC	Oan*TERT*	2/1930/F
6	GGAGGCCCCCAGCAGATGTGGGTGTAGCGTGCGC	Oan*TERT*	5/2489/R
7	TGACCGGGGCTTATGACACCATTCCTCACG	Oan*TERT*	6/2626/F
8	TCCACGTGAAAGTTCACCACGGTCTTCC	Oan*TERT*	11/3196/R
GAF	GTGGGCAAGGTCATCCCTGAGCTGAACG	Oan*GAPDH*	124/F
GAR	GTCGAAGGTAGAGGAGTGGGTGTCACTG	Oan*GAPDH*	336/R

[a] The name of primer as indicated in Figure 3a.
[b] The position of primer target sequence is indicated by the exon number / location of the 5' nucleotide in Oan*TERT* cDNA sequence [GenBank:JF441071] / orientation: F - forward, R - reverse. Only the location in the predicted mRNA sequence [GenBank:XM_001507850.2] and the orientation are shown for Oan*GAPDH* primers.

TABLE S1b. PCR conditions

Name[a]	Primers	T_M[b](°C)	T(°C), DMSO (μl)[c]
Oan*TERT*	1/2	64/64	63,0
OanA/A3	3/6	75/74	70,4
OanA2/A4	4/6	76/74	70,4
OanWT(A-A4, B)/B	5/6	72/74	70,2
OanWT(C)/C	7/8	66/66	66,1
Oan*GAPDH*	GAF/GAR	66/63	63,0

[a] Name of targeted mRNA as indicated in Figure 3.
[b] Melting temperature of primers.
[c] Annealing temperature of PCR reaction and the amount of DMSO used in 50 μl PCR reaction.

TABLE S1c. Primers used for cloning of Oan*TERT*

Primers[a]	Forward primer	Reverse primer
p1	GGGCCCCCTCGGTGGCCCCGGTGGCTTCGG	GGTGAGCGGGTCGGGGAGGGGGCGGGCTCC
p2	GGGCCCCCTCGGTGGCCCCGGTGGCTTCGG	CGACCCTGGGTCCGGGCCCCCGTCCCGGCC
p3	TGGCTTCGGCGATGGCGAGCGCGGCTCC	GGAGTCATCGGCGGGGAGTTCGTAGATG
p4	AGCGCGGCTCCTTTCTTTGCGGTGC	GGAGTCATCGGCGGGGAGTTCGTAGATG
p5	CCCAACTGCTCTTATCAGATCTGCGGGCAG	GTGGGCGTCCCTTCCGTTAATCCAAGATGG
p6	CCATCTACGAACTCCCGCCGATGACTCC	TCTCGGTGACGTAGAAGAAGGCTCGGAGC
p7	CATCTACGAACTCCCCGCCGATGACTCC	GTGGGCGTCCCTTCCGTTAATCCAAGATGG
p8	ACGCCGAGTCGGTGAGCGCGAGAGCGGAGC	GGCCGCACCACGGGAAGAGACAGCCGGCGGGGAGC
p9	GGAACAGACCCCCGGCCGCGGGCCTCCGGG	GGACGCCCCACCCGGCCCCCGGAGACGCCG
p10	GGAAGCGCCTCCTGTATTCCTCCAGG	TCTCGGTGACGTAGAAGAAGGCTCGGAGC
p11	GGAAGCGCCTCCTGTATTCCTCCAGG	GTGGGCGTCCCTTCCGTTAATCCAAGATGG
p12	GCTGCCCAGTCCGGGTCGGGGCCGTCTCCC	GGAGGCCCCCAGCAGATGTGGGTGTAGCGTGCGC
p13	CAGCCACAACCAAATGCCGCCTTCC	GTGGGCGTCCCTTCCGTTAATCCAAGATGG
p14	GCCGCTTCCTCAGGAACGTCAAGGCCTTCTTGGCCC	GAACGACGACGGCGTTCTGGAGAGACGTGGTCTCCTG
p15	GCGGAAGACCGTGGTGAACTTTCACGTGG	CCAGCCAAGGCACGTAGCTTTGGGGAGTCC

[a] The primer combination indicated in Figure S1a.

[b] The forward primers used in combinations p6, p7, p12, p13 and a reverse primer used in combinations p3, p4 were designed based on the available genome sequence and do not 100% match the final cDNA sequence. A forward primer in combinations p1 and p2 and a reverse primer in combination p15 are primers flanking the cDNA composit and their sequences were not included in the final cDNA sequence.
