# Supplementary material for: Insights into the evolution of mammalian telomerase: Platypus TERT shares similarities with genes of birds and other reptiles and localizes on sex chromosomes
Source: BMC Genomics. 2012 Jun 1;13:216. doi: 10.1186/1471-2164-13-216 (PMC3546421; doi:10.1186/1471-2164-13-216)
Supplement: Additional file 2 — Figure S1. Platypus TERT cDNA clone (PDF). (a) Cloning strategy. (b) The sequence comparison of the genomic contig with the platypus TERT cDNA. (c) The assembled sequence of OanTERT. (d) The region of genomic sequence flanking at 3′ side the sequence matching OanTERT cDNA. (e) The region of genomic sequence flanking at 5′ side the sequence matching OanTERT cDNA. [file 1471-2164-13-216-S2.pdf]

(a)

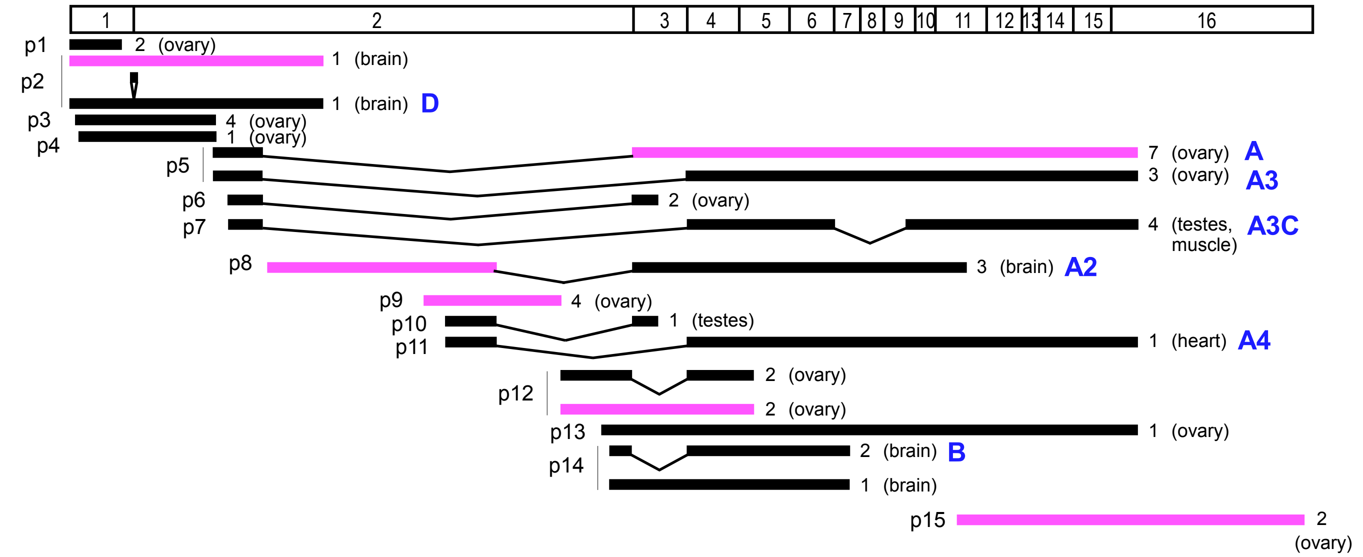

1 2 3 4 5 6 7 8 9 10 11 12 13 14 15 16
p1 2 (ovary)
p2 1 (brain)
1 (brain) D
p3 4 (ovary)
p4 1 (ovary)
p5 7 (ovary) A
3 (ovary) A3
p6 2 (ovary)
p7 4 (testes, muscle) A3C
p8 3 (brain) A2
p9 4 (ovary)
p10 1 (testes)
p11 1 (heart) A4
p12 2 (ovary)
2 (ovary)
p13 1 (ovary)
p14 2 (brain) B
1 (brain)
p15 2 (ovary)

(b)

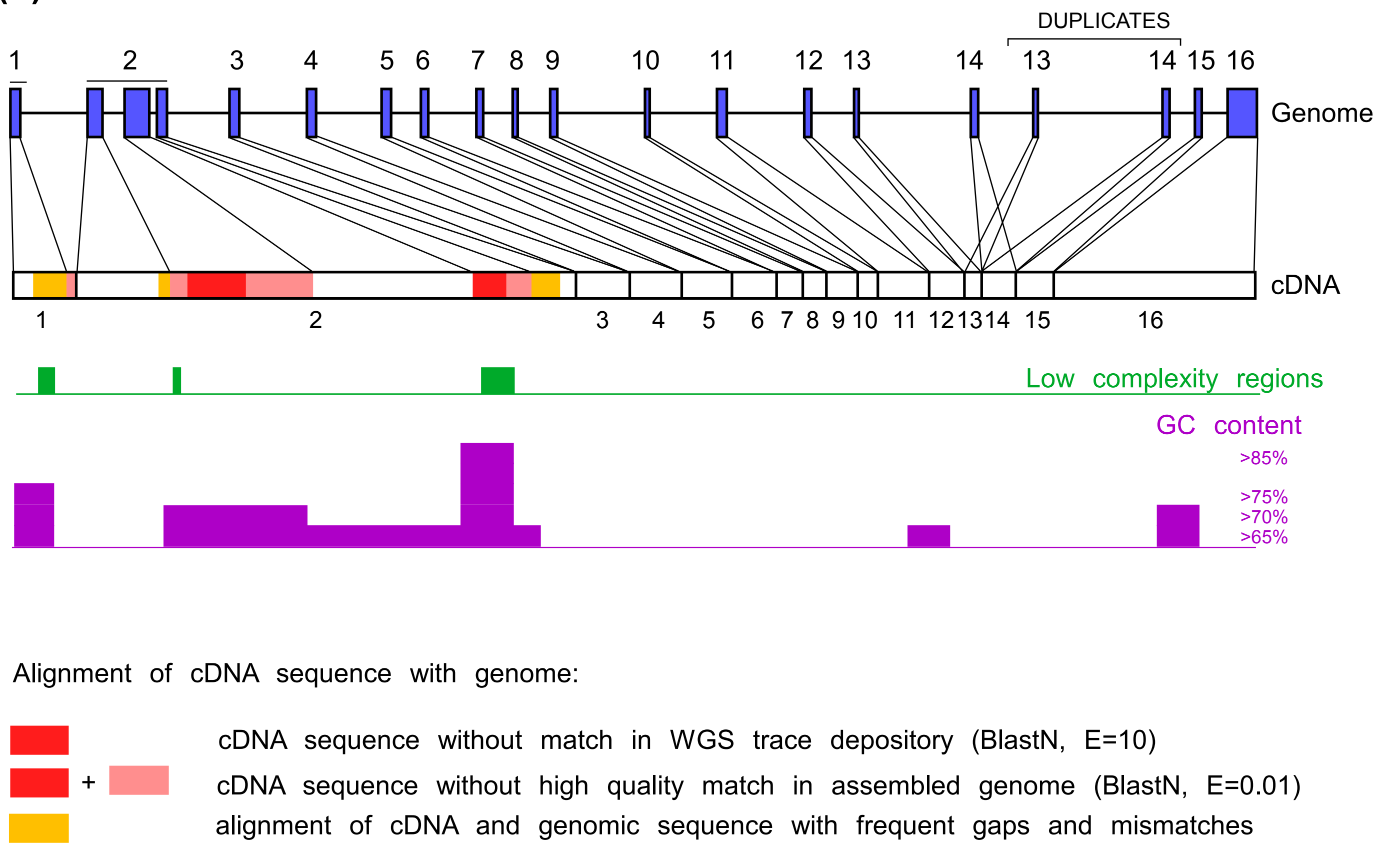

DUPLICATES
1 2 3 4 5 6 7 8 9 10 11 12 13 14 13 14 15 16
Genome
cDNA
1 2 3 4 5 6 7 8 9 10 11 12 13 14 15 16
Low complexity regions
GC content
>85%
>75%
>70%
>65%
Alignment of cDNA sequence with genome:
cDNA sequence without match in WGS trace depository (BlastN, E=10)
+
cDNA sequence without high quality match in assembled genome (BlastN, E=0.01)
alignment of cDNA and genomic sequence with frequent gaps and mismatches

(c)

```
CGATGGCGAGCGCGGCTCCTTTCTTTGCGGTGCACGCGGTCCTGCGGGCGCGCTACGCCGCCGTCCTGCCGCTGCCCGACTTCGTGGGGGGCCTCCCCGGCGCCCCCCGGGGGCTGCTGC
CGCTCGGGGATCCCGGGGACCCCGAGATCTTCCAAACCTTCCTGGCCCAGTGCGTCGTGTGCCTGCCCCGGGGAGCCCGCCCCCTCCCCGACCCGCTCACCTTCCGCCAGCTGTCGAGCC
AGAAGGAGATTGTGGCTCGGATCGTTCAGCGCATCTGCGAGAAGGGGAAGAAGAACGTGCTGGCGTTCGGGTACACCTTGTTGGACGAGAACAGCAGGTCAGAGCCGGTGGTGTTCACGT
CCAACGTCTGCAACTACCTCCCCAACATCTCCACGGAAAGCGTCAGGACAAGCATCCTCTGGGAGATGCTGTTCAGCCGGGTCGGGGACGACGTGATAATGTATTTGCTGGAACACTGTG
CCCTGTTTATGCTGGTGCCACCCAACTGCTCTTATCAGATCTGCGGGCAGCCCATCTACGAACTCCCGCCGACGGACTCCTCCCCTCCCCGCCCCTCCTCCCCTCGGTTCTTCCGACAGA
GAGCCTCCAACCGGAGGCGCGACGTCCTCTCGGAGTACGTCAGGGAGAAGATTCGTCTTCACGGGAGACGCGGGGAGCAGTCGGATCGGAAGCGGAGGAAACGCCGAGTCGGTGAGCGCG
AGAGCGGAGCCGGGGGCCGTAGGGAAACCCGTCTGCCGTCCAGACGCACGAACCCGGGTGGGCGTGAAAGGGACCGCCCCGTCACCGACGGGCCGAGAAAGCGGCCGGACCCCGGGCTCG
CTCCCCGGGCCGCCAAGCGATACCTCGAAAAGGACGAAAGGGGGACCTCCGGCAAGAGGCGGAAAGGGGCGGTTCTCGCCGGCCGGGACGGGGGCCCGGACCCAGGGTCGCCCAGCCCCG
CCACGCCCACGAGGGAGGTCCGGCCGGAAGCTCCGGGGTCTGAGGCGTCGGAGGCCTCCCGGGGTCTGCTCCAAATGCAGTCGGTTCCCGGGGGCCGCCGGGACCGGGAAGATTCCAGAA
TTCCATCAGATCCGCCATTCCCCGAGCGGGTCCGAGGGAACTTAGTCCGGCCGGGGCCTCGGGTTCCAAAAACCCGAGGTCTGGAGGCCCCATCCCAGCCCGGGAGTACGGGATCGGCTC
CCCCAGGATGGTCTTCCCCCGCCCGCTCCCCTCGGGAGGAAGAAAGCCCGAGATCGGGAGCCGGGGAACAGACCCCCGGCCGCGGGCCTCCGGGTTCAGTTCCGGAGAGTTCCCGGGCAG
CCTGCCGCCCTGGAGTCTTCATCGACAGGAAGCGCCTCCTGTATTCCTCCAGGGACCTGAAGGAACGTTTACCACGGTCCTTTCCGCTGAATCGCCTGCGGGGCGACCCGGCCGGGGGAC
GAGGCTTGGTGGAGGCCATATTCTTGGCCGGGAGCCTCCGGGAGGGGAGACGAGACCCGGCCGGTCGGAGGCTCGGCCGGAAGAAGCGGCGGCTCCCCGGACGCTACTGGCGGATGAGGC
ACGTGTTCCGGGAGCTGTTGCAGAATCACGGGAGGTGCCCCTACGGCGTCCTGTTGAGGAAGAGCTGCCCAGTCCGGGTCGGGGCGGCCGAGCCGGCCGCGGGCACCGGGCCTCCCGGCG
GGCCGGCCGGCGTCTCCCTCCGGTCGGCCGGCCGGGCCGGAGGAAGACGGACGGCGTCGCCGGCCGGCGGCTCCCCCGAGGGCTCCCGCCGCGCCGGGGCCGTCTCCCCGACGGCGTCTC
CGGGGGCCGGGTGGGGCGTCCACGACCTCCTCCGGCAGCACAGCAGCTCCTGGCGGGTGTACGGTTTCGTGAGGGAGTGCCTGCTCCGCGTGGTGCCTGCCGAGCTCTGGGGGTCCAGCC
ACAACAAATGCCGCTTCCTCAGGAACGTCAAGGCCTTCTTGGCCCTGGGGAAGTTCGACAAGTTTTCCCTGCGGGAGCTGAGGTGGAAGATGAGAGTCTCGGACTGTGCCTGGCTTCGGT
CCGGCCGAGGGGATCACTCCGTCCCAGCGTCGGAACACCGGTTCCGGGAGGAGATCCTAGCCAAGTTCCTCCACTGGCTAATGAGGGCCTACGTGGTGGAGCTGCTCCGAGCCTTCTTCT
ACGTCACCGAGACCATGTTTCAGAAGAATCTGCTTTTCTTCTACCGAAAATGCGTTTGGAACAGGTTGCAAGCCATTGGAATTAGAAACCACTTGGCCAAGGTGCAGCTTCAAGCAATAT
CTGAAAAGGAAATTAAAGAAAACCTGTCCAAAAAATATGTTCCCGTGGTATCCAAACTCCGGTTCATCCCCAAATCCAACGGACTGCGGCCGATTGTGAATGCAGACACCATCGCCGGAG
CAAAGTTATTCCCAAAAGAAAGCAGAGATAAGAAGGTTCAGTATTTCAACACCCAACTGAAAAACCTATTTAGTGTTCTGAATTACGAGCGCACGCTACACCCACATCTGCTGGGGGCCT
CCATGTTTGGGATCGATGACATTTATAAGGCATGGCGGCAGTTTGTGCTCAGAGTTCTGACGTCTAAGGATAAAGAACGCAGATTTTATTTTGTGAAGGCCGACGTGACCGGGGCTTATG
ACACCATTCCTCACGACAAGCTTGTCGAAGTGATCTCAAGAATCTTGAAGCCCGAAGACGAGACCGTCTATTGCATCCAGCGTTATGCCGTGATCCAGAAAACGGCCAAAGGCTCCGTTC
GGAAAACCTTTAGGAGACATGTTTCCACGTTGAAGGACTTCCCGCCCTCCATGAAGCAGTTTGTGTCCCATTTACAGGAGACCACGTCTCTCCAGAACGCCGTCGTCGTTCAGCAGAGCT
CCTCTCTCAACGAGACCAGCACCAGCCTCTTCGCTTTCTTTCGGCACATGATTCAGCACAACATCCTGAAGATAAGAAACAGGTATTACGTCCAACGCCGAGGGATTCCACAGGGCTCCA
TTCTGTCCACGCTGCTCTGCAGCCTGTGCTACGGAGACATGGAAAACCGTCTGTTCCCCGGGATCCAGCAGGATGGGGTGTTGCTCCGCTTAATCGATGACTTTTTGCTGGTCACGCCCC
ACTTAGCCCAAGCCAAACTCTTCCTACGGACTCTGGCGAAAGGAATTCCCGAGTACGGCTGTTTCATCAACCCGCGGAAGACCGTGGTGAACTTTCACGTGGACGAGGACACCGTGGGGT
GTTCTCATTTTACTCAGCTCCCCGCCGGCTGTCTCTTCCCGTGGTGCGGCCTGCTGCTGGACACCCGGACCCTGGAGGTGTACTGTGACTACTCCACCTACAGCCGGACATCGATCAGGG
CGAGCCTGTCCTTATGTCAGAGCACCTCGGCTGGAAGGAGCCTGAGGCGAAAACTCCTGACCGTCCTGAAGCTGAAGTGTCACGGCTTATTTCTGGATGTACAGGTGAACAGCCTCCGAA
CGGTCTGCATCAACATTTACAAGATATTTTTGCTGCAGGCCTACAGGTTTCACGCCTGCGTCCTCCGGCTTCCATTTAATCAGCGAGTCAGGAAGAATCCGGAGTTCTTCCTCACGGTCA
TTGCCGACACGGCATCTTGCTGCTATTCCATGGTACGAGCCAGGAATGCAGGGGTTACTCTCGGAGCCAGAGAAGCTTCAGGCCCGTTTCCTTCGGAAGCAGCACAATGGCTTTGCTATC
AGGCCTTCCTGACTAAGTTGGCAAATCACAAAGTTGTGTACAAGTGTCTTCTGGGGCCCCTCAAAAGGAGTAAACAGCAGTTGCTTCGAAGACTCCCCGGGCCTACGATGCTGATGCTAG
AAGCTGCGACTGATCCAATGCTCTGTCAAGATTTTAAAACCATCTTGGATTAACGGAAGGGACGCCCACTTCTCTTGGACACCTCTATGGGGATGACTTTCACCCGGCTGCCAGCAAAAG
CCAAATTGCTCACTAGTTGTCCCTTGCGCAGCGAGAGAGTGTCCCAGCTTTTGCCTAAGCCGGATCTATGCTGTCGCTAGCTGTCGGCTCTTGGTCTCGTCCTCTTTCTCCTGCGTCGCC
GATGGCTCCTCTGGGTCTGTGCTAATTCACTTTCAGCAAGTGTCTAACCAACTGTCAGCTTATGGTACACCCAGAGATTCCTTTGTCCGCCCCTGCGCCTGGTCACCTTCGCAATCAAAG
CCGCTCTGGGCTTCGCTCCGAGTGATTTCCAGGGAAAGCGATCCCACTCCAGCATCCCGGGACCCCCTCCCCTCCATCCGCCGGCCCCGCGGGGCCAGGGGAGGGGAACCGGGAAGGAAG
AAGCGGGATGGGCTGTTGTTCTTCGACTGTGGAGGAAGTGAAGGGACGTTCCACATCGACCAGGATGAAGATGACCCCTTCGGGACCACAAAAGTTTCGAGCAGCTATGCGAGAGAATAA
TCACTGTTGCTTCTGCACCGTGACTCTATGACCCCCTATTCGGGCCTCTTCCCATTTTGATTCCTGCGGGTATGGGAATGCG
```

(d)

cDNA ends

```
AATGCGGGACTCCCCAAAGCTACGTGCCTTGGCTGGAATAGAGGAGCACTCCCCCCCCCCCCCCCCCGCCCCGGCTGCTGCAACCTC
AAATTTCTCTCCTACTCCCCTCCAGAAAGTAGGGTCTTCCTTCGTGACTTCAGTCCCCCAGGGAAACCTCCAGGAATGTGAGAGAAGA
AATCCAATTGTGGCT
```

(e)

```
TGACCCCGCCCCCCTCCCGCAGCTCCAGGAGGCCCCGCCCCCTTCGGGGCCCCGCCCCCTTCTGGCCCCGCCCCCCTCGGGGCC
CCGCCCCCCTCGGGTCGGGCCCCCTCGGTGGCCCCGGTGGCTTCGGCGATG
```

cDNA begins

Figure S1 Platypus *TERT* cDNA clone.

(a) (a) Cloning strategy. A schematic representation of the assembled OanTERT cDNA sequence with the 16 regions predicted to be encoded by separate exons is shown at the top. Nineteen different types of RT-PCR clones obtained from platypus mRNA using 15 distinct primer pairs (p1-p15) are depicted below. Cloning primers are listed in Table S1c (Additional file 1). Forty-four clones were sequenced and the number of clones of each type and the source tissue are shown on the right of every clone schema. Continuous consensus sequence was assembled by DNA Baser (Heracle Biosoft S.R.L., Pitesti, Romania). The full-length TERT sequence is based on six overlapping DNA segments (without primer matching sequences) shown in purple ink. Ten types of the clones were alternatively spliced. Insertion of the intronic sequence and deletions resulting from alternative splicing are indicated. The clones which sequence was submitted to GenBank as OanTERT alternatively spliced variants are designated by abbreviation in blue ink (A - D). In some of the clones we noted several single nucleotide deviations from the consensus sequence. These variations were included in the submitted sequences only when confirmed in two independent PCR reactions to exclude possible PCR artifacts. All clones are shown without the primer matching regions - consequently the actual clones have approximately additional 20-30 nucleotides of platypus TERT sequence at each of the ends.

Figure S1 Platypus *TERT* cDNA clone - continued.

(b) The sequence comparison of the genomic contig [GenBank:NW_001794359.1] with the platypus *TERT* cDNA [GenBank:JF441071]. The major areas of misalignments and sequence gaps in genomic sequence are indicated. The low sequence complexity regions in cDNA determined by BlastN build in filter and the areas of cDNA with high GC content determined by GC-Profile program (http://tubic.tju.edu.cn/GC-Profile/) are indicated below the cDNA schematic representation. The genomic assembly also contains a duplication of the exons 13 and 14. Because the corresponding areas of the cDNA do not have any abnormalities, the duplication in the genomic assembly is likely the assembling error.

(c) The assembled composite sequence of Oan*TERT* [GenBank:JF441071]. Start and stop codons are indicated by white letters on the blue background. The boundaries of regions predicted to be encoded by 16 different exons are shown (the boundary nucleotides are on the green background) as well as two alternative splice donors and one alternative splice acceptor (the boundary nucleotide of the alternatively spliced exon are on green background with two neighboring nucleotides creating the beginning or end of the alternatively spliced intron on the yellow background).

(d) The region of genomic sequence [GenBank:NW_001794359.1] flanking at 3' side the sequence matching Oan*TERT* cDNA. The end of the cDNA sequence and two closest possible polyadenylation signals are shown.

(e) The region of genomic sequence [GenBank:NW_001794359.1] flanking at 5' side the sequence matching Oan*TERT* cDNA. The beginning of cDNA, ATG start codon, and the first upstream in-frame stop codon are indicated. The platypus genomic sequence does not contain any ATG codon between the start codon and the closest upstream in-frame stop codon supporting the correct determination of the ORF start codon. The encoded N-terminal amino acid sequence of the platypus TERT (MASAAPFFAVHA sequence preceding the TEN (telomerase essential N-terminal) domain) has the same size as that of human and mouse TERT.
