# Supplementary material for: Insights into the evolution of mammalian telomerase: Platypus TERT shares similarities with genes of birds and other reptiles and localizes on sex chromosomes
Source: BMC Genomics. 2012 Jun 1;13:216. doi: 10.1186/1471-2164-13-216 (PMC3546421; doi:10.1186/1471-2164-13-216)
Supplement: Additional file 3 — Sequences not available from sequence databases (PDF). All these sequences, except Δ2p(136-end), can also be retrieved in text format from the Additional file 6. (a) Corrections of TERT protein sequence models. (b) De novo generated TERT protein sequence models. (c) Human AS TERT variant Δp2(136-end). [file 1471-2164-13-216-S3.pdf]

Sequences - Corrections of TERT protein sequence models

The model sequences of chimpanzee (*Pan troglodytes*), elephant (*Loxodonta africana*), opossum (*Monodelphis domestica*), zebra finch (*Taeniopygia guttata*), and anole (*Anolis carolinensis*) TERT proteins were retrieved from GenBank or Ensembl databases. Because no full-length cDNA clones are available for these genes we have manually checked these GenBank and Ensembl models against the GenBank whole genome sequence databases of corresponding species and we are suggesting following small scale corrections (shown in purple ink below). These corrections use data from following GenBank entries: [GenBank:NW_001235370.1, AAGU03019639.1, NW_001581900.1, NW_002198278.1, AAWZ02016664.1]. The corrections are based on the optimizations of the alignments with other vertebrate TERT proteins and the modification of features that would violate the conserved 16 exon-structure of the vertebrate *TERT* genes. The corrections include extensions or modifications of the N-terminal sequence, translation of the sequence that was considered intronic by the models and replacement of the amino acid sequences with X symbols where the underlying genomic sequence contains sequencing gaps. N-terminal extensions of zebra finch and anole TERT proteins are suggested based on the existence of N-terminal extensions in chicken and quail TERT proteins. All other corrections are strongly supported by the significant alignment (or misalignment for the X-substituted sequences) with corresponding regions in other TERT proteins.

```
>ptrTERT    Modified version of gi|114598857|ref|XP_001141663.1| PREDICTED:
telomerase reverse transcriptase isoform 2 [Pan troglodytes]
MPRAPRCAVRSLLRSHYREVLPLATFVRRLGPQGWRLVQRGDPAAFRALVAQCLVCVPWDARPPPAAPSFRQVSCLK
ELVARVLQRLCERGAKNVLAFGFALLDGARGGPPEAFTTSVRSYLPNTVTDALRGSGAWGLLLRRVGDDVLVHLLARC
ALFVLVAPSCAYQVCGPPLYQLGAATQARPPPHASGPRRRLGCERAWNHSVREAGVPLGLPAPGARRRGGSASRSLPL
PKRPRRGAAPEPERTPVGQGSWAHPGRTRGPSDRGFCVVSPARPAEEATSLEGALSGTRHSHPSVGRQHHAGPPSTSR
PPRPWDTPCPPVYAETKHFLYSSGDKEQLRPSFLLSSLRPSLTGARRLVETIFLGSRPWMPGTPRRLPRLPQRYWQMR
PLFLELLGNHAQCPYGVLLKTHCPLRAAVTPAAGVCAREKPQGSVAAPEEEDTDPRRLVQLLRQHSSPWQVYGFVRAC
LRRLVPPGLWGSRHNERRFLRNTKKFISLGKHAKLSLQELTWKMSVRDCAWLRRSPGVGSVPAAEHRLREEILAKFLH
WLMSVYVVELLRSFFYVTETTFQKNRLFFYRKSVWSKLQSIGIRQHLKRVQLRELSEAEVRQHQEARPALLTSRLRFI
PKPDGLRPIVNMDYVVGARTFRREKRAERLTSRVKALFSVLNYERARRPGLLGASVLGLDDIHRAWRTFVLRVRAQDP
PPELYFVKVDVTGAYDTIPQDRLTEVIASIIKPQNTYXXXXXXXXXXXXXXXXXXXXXXXVSTLTDLQPYMRQFVAHL
QETSPLRDAVIIEQSSSLNEASSGLFDVFLRFVCRHAVRIRGKSYVQCQGIPQGSILSTLLCSLCYGDMENKLFAGIR
RDGLLLRLVDDFLLVTPHLTHAKAFLRTLVRGVPEYGCVVNLRKTVVNFPVEDEALGGTAFVQLPAHGLFPWCGLLLD
TRTLEVQSDYSSYARTSIRASLTFNRGFKAGRNMRRKLFGVLRLKCHSLFLDLQVNSLQTVCTNIYKILLLQAYRFHA
CVLQLPFHQQVWKNPTFFLRIISDTASLCYSILKAKNAGMSLGAKGAAGPLPSEAMQWLCHQAFLLKLTRHRVTYVPL
LGSLRTAQTQLSRKLPGTTLSALEAAANPALPSDFKTILD
```

```
>lafTERT    Modified version of Ensembl gene: ENSLAFP00000010943 Description:
telomerase reverse transcriptase Species: Loxodonta africana
MPRAPRCAVRALLRGRYREVLPLATFAQRLGAEGRRLVRRGDPAAFRSLVAQCLVCVPWGAPPPPTAPSFRQVSCLK
ELVARVVQRLCERGAKNVLAFGFALLDEARGGPPMAFTTSVRSYLPNTVTDTLRGSGAWGLLLQRVGDDVLAHLLARC
SLYLLVAPSCAYQVCGLPLYELCARDTRGPEPGCGAPRRTVGGTRRRWDSAGERHPLAKRSRHSLALEPGQVPQSWAH
LDRACGQSDSDPHAVTPGRRAAAKAVSWEGEIAGRRRSSTLGVKERGAGPSSAQPPLSLLPQAYVETKRFLYCLGAKE
RLPSTFLLSSLQGNLTGARRLVETIFLRTKPAWRKTQLRRLTPRYWRMRPLFQELLGNHARCPYSVLLRTHCPRLGSA
MATPAGTGGAGTGTPEQPPGPAVKPAPEEDAGLQCVVQLLRQHSSPWQVYTFVRACLHRLVPPGLWGSSHNKCRFLRN
VKMFISLGKHAKLSLQELMWKMKVQDCEWLRRSPGDYAVPASEHRLREEILAKFLFWLMDTFVVGLLRAFFYVTETMF
QKNRLFFFRKSVWNKLQSIGIRQHFNKVRLRELSEAEVQRRQQTKPSLLTSKLRFVPKAHGLRPIVNMDYVVGARTFR
REKKRLQHFNSQMKNLFSVLNYERAVRPGLLGASVLGIDDVYKAWRAFVQQVRAGDPRPQLYFVKVDVTGAYDAIPHN
KLVEVIANVIKPKENIYCIRRYAVVQKTAQGYIRKSFKRHQVSTLADLLPYMKQFVEHLQETSSLKNAVVIEQSSSLN
EPASSLFDFFLHLVHNNVIKIRGKYYVQCQGIPQGSILSTLLCSFCYGDMENKLFSGIQEDGLLLRLVDDFLLVTPHL
TQAKAFLSTLVRGVPEYGCMINLRKTVVNFPVDDGPGQATILQLPAHCLFPWCGLLLDTRTLEVFCDYSSYARTSIKA
SLVFNHGFKAGRNMRRKLFAVLRLKCHGLFLDLQVNSLQTVYINVYKIFLLQAYRFHACVLQLPFNQQVGKNPSFFLR
VISDTALRCYSILKAKNAGMSLGAPGAVGPFPSEAARWLCHHAFLLKLSRHRVTYKCLLGTLKTTKMQLGRKIPKATM
ALLEAAAAPSLCGEFKTILD
```

```
>mdoTERT    Modified version of gi|126320879|ref|XP_001369432.1| PREDICTED:
similar to telomerase reverse transcriptase [Monodelphis domestica]
MASNPAVSGSLLASVAFRAVRSVLQARYRDVLGLAEFVQRLGDEAQAEGGSGADVQLLRGGEPEVFQVFVSQCVVCVP
WDARPPPRPLTFQQLSSQKEVVARIVQRICEKKKKNILAFGYTLLEEKRMSLPVMFTTNVYNYHPNTITETISVSALW
EMLLSRIGDDVMMYMLEHCSLFMMVPPSCCYQISGLPIYDLYLKDSTPPSGFVQRTYSKQGANASLDNVRKISLLSKS
LAKSNLRKEMLGSKEKAIKVVQQNQNSTEDPEDKSLRGRPEESCDQGEQVRTHSVHSTMALLSKRQREDEEKSEISAK
RSKTEELLQEKRKELILGQELHENNESNLDNVESLTQRSMEICSSRPLFNEKSIHKEDGGGEGCFIKTKGSLSLLEHK
DGDLSRHNSSVIRSTLKGKAKARSSEGCLNRGAKEADATFSHPLCPGNEPPKGLKSTSSSIIYINRKRFLYSAWNFRE
CLPTSFLLNRLHDSLSGGQKLVETIFLTSHLFGQKGDPQQQRTPWKKRRLPKRYWQMRPLFKELIQNHRKCPYFILLK
KNCPLRFSAAKADPSSQLTKPQAMASSHLTQAEGIQGETQDQGHQGEHTSRASNSMEEEPSSSLKDQISSKESSLSDA
VSKKALPASHEVGKTDKGGGDILQLLRWHSSPWQVYVFLRECLHRLVPAELWGSTHNKCRFFKNVRKFLTLGKHDKFS
LRELMWKMRVKDCAWLALVRGNHFIPASEHRLREEILAKFVYWLMNTYVVELLRSFFYITETMFQKNRLFFYRKCVWS
KLQNIGIRKHFEKVHLKLLSEEEIKQNPEARFICLASRLRFVPKPTGLRPIVNVDSIIGARVSNKGTKDKKIRCFNGQ
LKNLFSVLNYERTLHPEVLGSSLFGIDGIYKEWRQFVLRVKPSKDKISNFYFVKADVTGAYDTIPHEKLIEVISSIIK
PKEKKVYCIRRYAVVQKNAHGNIQKSFKRHVSTYKDALPYMSQFVSHLQETTSLQNAIIVEQXXXXXXTSINLFAFFL
HLIRNNILKIKNKYYVQCQGIPQGSILSTLLCSLCYGNMENKLLSGIQQDGLLIRLIDDFLLVTPHLTQAKVFLRTLA
KGIPEYGCLINPKKTVVNFPVEEDILSDSDFTQLPAHCLFPWCGLLLDTRTLNVFCDYSNYSRTSIRTSLSFDHSIKA
GRNMRNKLIAVLRMKCHGLFLDLQVNSLQTIYINVYKIFLLQAYRFHACALKLPFNQQVRKNPGFFLSIISDIASCCY
SILKAKNPGVILGARGGSGPFPFEAARWLCYHAFLIKLVNHRIVYKCLLGTLKMSKLQLFRKIPKATMQMLRVASDPS
LSQDFKMILD

>tguTERT    Modified version of Ensembl gene: ENSTGUP00000008676 Description:
telomerase reverse transcriptase Species: Taeniopygia guttata
MPGGRAAWSQGRRGCARWTRAQFGRNMAGKEPFAAVLSALRRCYAEAVPLETFVRRLGDGGAGDAEVLRADDAPGYRN
FVGQCLVCVPRGARAIPRPFTFQQLSSQSEVTARVVQRLCEKKKKNILAYGYSLPDENSSQFPVMPLSKIHSYLPNTA
TETLCISGFWETLLSRIGDDVMMYLLEHCAIFMLVPPNNCYQVCGQPVYELISHSVDSSLVFVRQRFSKYKRTSLLKY
MQKRLMFHRNYLLKSSRQRYEENVSRMRNERKNKRRSLVPTDQSSAKTVSKGSNQIRMVTEDQEKQSSSSSCVSATAL
SLKRKIHREQFEIPAKKAKMGEKVREEKACSLVPNVNQSSSERSETGYVAEHSESVIKTPCISERSNSAVSGPSLVHT
SCGRRKSVAVSLLQRFQSNKPLESNTEMQAESHRKRVEIRMYESQLASGQTKPVKGSKCRQQESPQPHLSKKLPNRLL
SSATYIERKSLLYSCRSFQECFPKSFVLNRLQGSQAGGRQLVEAIFLSQNQVQQRHNQSLPKHKRRKKTLPKRYWQMR
HTFQQLLMNHGKCAYLALLKKNCPVWISEISMTKTKLSCQATLSEEAEVQKQAEQFGKEPAKCLTSSRCGSDHTDLPD
NLGAPLAESVRGELPPSEEQNPREARDSALTELLKQHSSHWQVYMFVRDCLEKVIPAELWGSNHNKCRFLKNVKVFIS
RGRFAKVSLQELMWRMRVNDCMWLRLGKGDHFVPADEHCFREELLAKFLYWLMGTYVVELLRSFFYITETMFQKNMLF
YYRKFIWGKLQNIGIRNHFVKVQLRPLSSEEIETIRQKKIVPVALKLRFIPKPNGLRPIVKVSGVVEPQALSKESREK
KMNHYNTQLKNLFSVLNYERTINTSFIGSSVFGKDDIYKTWKQFVIKILESGGEIPHFYCVKADVSRAYDTIPHNKLV
EVISRVLKPEKRTVYCIRRYAVIMITPSGRAKRLYRRHVSTFKDFMPDMKQFVSQLQENASLQNAIVVEQSLTFYETS
SSLFNFFLQMIHNIILEIRNRYYLQCCGIPQGSILSTLLCSLFYGDMENKWLPGIKQDGVLIRLIDDFLLFTPHLMKA
RTFLRTLTTGIPEYGLLINPNKTVVNFPVDDIPGCSKFKQLPDCRLIPWCGLLLDIKTLEVYCDYSSYTCTSIRSSLS
FNSSVTAGKNMKYKLSAVLKLKCHSLFLDLQINSLRTVLINIYKIFLLQAYRFHACVLQLPFNQQVRKNPHFFLRIIS
QTASCCYAILKTINAGIAEGNKGLSGIFPIQVAEWLCYHAFTVKLLNHKAVYKCLLTSLKVCKRKLIRKIPEDTVALL
QAVTEPSLCQDFKAILD

>acaTERT    Modified version of Ensembl gene: ENSACAP00000001407 Description:
telomerase reverse transcriptase Species: Anolis carolinensis
MQKIEGAAFPALLPPRPRRGGPGQAMRRSQVCRLLRGCFEEVLPLEAFVKRLQEKEAKAGGLPAEPLIQDGDPKCFRV
LVERCLVGRPRGGKAPPPRLVFQQIFSQHDIIARVIRRICEKKKKNVLAFGYDLLDENHFPLPHMPNLYSYFPNNTTE
TICQSILWEKILNRVGDDFLMYILEHCSLFMLVPPSCCYQICGQPVYEIAFKDSTSFPKFLRQRYPGPKHSTLSGYLR
RRRFSSYKQHTARGNRKKWHPRRKLGSKANNILEGSYQQSLLIQTVKQNFTVSASECPESKQRTSECKSLTTRSLKRK
WKGHYEMSAKRMKIMKIEDGLQKETGNLVHTQSKHQLSLDGDNAASKSSTSFCLADQLTPVTSVLHSNECGEQISGVH
VAHLDHRKSFLSSKTMTLVSGSKAHCEPSTKIDSVDRSTKQEGGIRSTQMVSAKGATARRDFRVHNSTSSEKSSDNTS
FKRYSLLYCHRQLHECLPNSFVLNKLKGSPSGGQSLVEIVFFTSQIPKQLDSSNQSHSKRKKRLPKRYWQMRGLFQEL
LQKHAKCPYLGILKRNCPIWISDSIRYGTEEQACEEKVNQERRHSSQESQEANTIEYCPSSVTVGLGHFPRTSGASSR
SCGETELGQKVPEEQPILDSSTSNFRGLLKQHSSHWQVYTFVRECLQRVVPAELWGSSYNKCRFYKNVKKFISLGKLA
TFSMQELMWKMRVNDCTWLRLSKGPGHFVPASEHHFRQDLMSKFFYWLMDSYVTELLRSFFYITETMFQKNLLFFFRK
TVWSKLETIGLRNHLAKVHLHALSEEKIKNLQQEKYVPLASKLRFIPKTNGLRPVVRLDSVVGAKTFCEKIRERKVQL
FKTQLKNLFSVLNYERIKNPALLGSSVFGKDDIFAAWKQFVLKILELNEEMPKFYFVKADVMGAYDSIPHDKLEEVVL
QALSPNKKTITYSIRRYAVIIRTRNGLLRKYYRRHASTYKEFKPEMNHFVSHLQESTSLRNAVVVEQSISLKETSSHL
SEFFSRLIRNSILKIKDSYYVQNCGIPQGSILSTLLCNMCYGDMENKLLRGIQKDGILMRLTDDFLLVTPHLTQAKTF
LRTLAMGIPEYGFVINASKTVVNFSVDEDIPGLSGFKQLPSHCMFPWCGLLIDTQTLEVYCDYSSYSCTSIRSSLSFN
SSVKAGVSMRNKLLDVLKLKCHSLFVDLQINSLRTVCINVYKILLLQAYRFHACVLQLPFDQKIKSNPSFFLGIISQT
ASCCFCILKTKHSDIPFSAAGISSPLTYKAVQWLCYHAFSVKLAIHRVIYKCLLVPLAQSKKRLLQQMPEATVQLLKE
VTEPSIYNDFKTILD
```

Sequences - *De novo* generated models of elephant shark (*Callorhinchus milii*) and tammar wallaby (*Macropus eugenii*) TERT proteins

The models were derived by computational analysis based on the alignments of vertebrate TERT protein sequences with the whole genome sequences (WGS) of the respected species and on the conservation of 16 exon-structure of vertebrate *TERT* genes. Some exon sequences were not found in the current WGS databases. The sizes of these missing regions were estimated based on the expected similarity to respective regions of other vertebrate TERT proteins.

```
>cmiTERT    Predicted Callorhinchus milii telomerase reverse transcriptase
XXXXXXXXXXXXXXXXXXXXXXXXXXXXXXXXXXXXXXXXXXXXXXXXXXXXXXXXXXXXXXXXXXXXXXXXXXXXLS
AQSDVVLRVIQRICEKGKKNVLAFGYANVTETSSMSVRFAPNLCSYQPNPTTVTIKNSILWKTLLSRIGDDVMMYLLE
HCSLFMLVKPSCCYQLCGVPIYSLITAGTRLPALWLRRRPIRARFNILLKIVQKRIRFHKGFLLKRKRRTLVDRDSCL
DARGTTECNKQKWTQIVSVHTRKPAKRAKLDTDIAKDTPRDVSSTGTKVTEVRPLKRLLHDEDKAESPAKKVKRDRSP
EGAIPHSTPPEILSAGNKMNESPENVEVSDSTSIHSTRGGLVEQKVIDAKTGVGCQRKYPQGSTRERKRTWQEGTIAG
YKRQAPGTDAKATHPTGAGGVEVKRSWHFMGVQEAVSFGKSVNSESNEEKVLLEGRGSESKGKQAKVAKRKDESGKRC
AKFAAGKDKRGTKRAASNEGDDNDHFNPGRSAKHGQSTKCIAKNDYSWDGLNPDGQCRAAVGVSGQAKPSPIPADASD
AATVKNVRTWGSVYVERGHIIYCNDNRECLPKSFLLNCLQGCSSGGQRLVEAIFLSSDAFGNNGKKQPNNYWRKRRLP
KRYWKMKNVFFRLLRNYKRCPYRLLLNRNCAVIIRKDNGVSCSEGNPTPSVNESNSFSTVKMPCKPDSDHLEVNRVTG
GATNLIATQGPVLRRKNLEDHTSVNKSVPGLSCPNDLHLEAETGSLQPSEIPNLRCQHKRPTGIPAGKQKVNKELKVF
ADFDQTGMASGSDGDLLQLLKHYSSPLQVYRFVRECLLRVISDELWGSNHNKYRFLKNVKKLISLGKYDRFSLSELMW
KMRVNDCTWLQLNNGQCSVSPSEHRLREEILSKFLLWLMGTYVVHLLKSFFYITETMFMKNMLFYYRKCVWNKIEKIG
IRNHLAKAQLQPLSKEEWARKQRQKTAIPFYALRFIPKRNGLRPIVKMRNITGSKKCVKGSNARKXXXXXXXXXXXXX
XXXXXXXXXXXXXXXXXXXXXXXXXXXXXXXXXXXXXXXXXXXXXXXXADVTGAYDTIPHAKLVDVISRVLDPKVQEN
YCIRRYASIWSNSAGQIRKSFKRQXXXXXXXXXXXXXXXXXXXXXXXXXXXXXXXXXXXXXXXXXXXXXXXXXXXXXX
XXXXXXXXYVQRRGIPQGSILSTLLCSLCYGDMENQXXXXXXXXXXXXXXXXXXXXXXXXXXXXXXXXXMLTAGIPEY
GCFIHPNKTMVNFPLDNDLLGCINVKTLQEHCLFPWCGLLLNTQTLEVYCDYSSYANTSITSSLTFNCSSKAGQNLRQ
KLLAVLKLKCHQIFLDLEVSTLRTVSINVYKIFLLQAYRFHACVMRFPFGQRVRDNPSFFLSVISDMASCCYSILKTK
NEXXXXXXXXXXXXXXXXXXXXXXXXXXXXXXXXXXXXXXXXXXXXXXXXXXXXXXXXXXXXXXXXXXXXXXXXXXXX
XXXX
```

This model (cmiTERT) uses data from the following GenBank WGS entries: [GenBank:AAVX01020497.1, AAVX01037780.1, AAVX01011403.1, AAVX01240754.1, AAVX01301389.1, AAVX01019362.1, AAVX01016929.1, AAVX01315420.1]. The model adds one nucleotide (N) to the sequence derived from AAVX01019362.1 to correct for the break in reading frame. Sizes of the missing regions are based on the corresponding regions of *Xenopus* TERT (the TERT protein with determined sequence phylogenetically most closely related - see Figure 2 of the article).

```
>meuTERT    Predicted Macropus eugenii telomerase reverse transcriptase
MASRPPAPRSLLASAAFRPVREVLRDSYRDVLGLAEFVQRLEDEARTARAPGDRPDQQVAVRLLRSGDPEVFQVFVSG
CVVCVPWGARPLPRPLTFRQLSSQKEVVARIVQRICEKKKKNILAFGYTLLEENRMSLPVKFTTNVYNYHPNTVTETI
SVSALWEILLSRIGDDVMMYMLEHCSIFMMVPPTCSYQISGLPIYELNLKDSVSPSGFLRKKYPKEEPNVSLDNVRKK
ISFQKKSSVKLDVRKAILRSKDKTLKVAGQNQNSAEDMVEEPLEDRPENSCGIEQGSNADQLEEPAHSAHSMMALLSK
RQRKDEEESEISAKRPKKEKCLLEKRKELILEQGFELHENESNSDSAENITQTSMEICSSKHFCNEKSALEEDGGRED
CFIETKATKGGLSLLEHNEGDLSCHDTSIIKSTSEDIIEAKSSEEGLDGGTEQKDAIFTQPLCPGNEQPKRLRSASGS
IVYINRKRFLYSARNFRECLPTSFLLNHLHDSLSGGQRLVETIFLTSHLFEQKGDPQQQRTPWKKRRLPKRYWQMRHL
FKELIKNHRKCPYFVLLKKNCPLRFSAAKADPSSQLKKPQSVASSQLTKAEEVQHQSQDQGQQREQSSETPLSCGSNN
TEKPSCSLADQMPDKESVLASRISQNTQPVSCASGKSVFRGKGGSDVHQLLSWHSSPWQVYMFLRECLHRLVPAELWG
STYNKCRFFKNVRKFLTLGKHDKFSLRELMWKMRVKDCTWLALVKGNHFVPASEHRLREEILAKFVYWLMDTYVVELI
RSFFYVTETMFQKNRLFFYRKCVWSKLQNIGIRKHFDKVQLRALSEEEIMQNLETRFICLASRLRFIPKPNGLRPIVN
VDSIVGAKVSNKGTKNKKIRCFNGQLKNLFSVLNYERTLNPDILGSSLFGIDGIYKEWRQFVLRVRQSKDQVSNFYFV
KVADLRGAYDTIPHDKLVEVISSIIKPEENKVYCIRRYAVVQKNAHGNIQKAFKRHXXXXXXXXXXXXXXXXXXXXXX
XXXXXXXXXXSSSLNETSMNLFTFFLHLIHNSILKIKNKLYVQCRGIPQGSVLSPLLCSLCYGNMENQLLPGIQQDGL
LIRLIDDFLLVTPHLTQAKVFLRTLAKGIPEYGCLINPKKTVVNFPVEEDILSDSDFTQLPAHCLFPWCGLLLDTRTL
NVFCDYSNYSRTSIRASLSFDHSVKAGRNMRNKLIAVLRLKCHGLFLDLQXXXXXXXXXXXYKIFLLQAYRFHACVLK
```

LPFNQQVRKNPGFFLSVISDIASCCYSILKAKNPGFTLGARGASGPFPFEAVLWLCYHAFLIKLVNHRLVYKCLLGTL
KMNKLRLFRKIPRSTMQMLKTAMDPLLSQDFKMILD

This model (meuTERT) uses data from the following GenBank WGS entries: [GenBank:ABQO010931898.1, ABQO010102539.1, ABQO010292497.1, ABQO010291975.1, ABQO010152530.1, ABQO010711080.1, ABQO010883553.1, ABQO011205628.1, ABQO010179239.1, ABQO010292459.1]. The sequence of the ABQO010711080.1 entry was extended by 12 nucleotides derived from the trace file [GenBank:gnl|ti|1658238143]. The model adds one nucleotide (N) to the sequence derived from ABQO010102539.1 entry and removes one nucleotide (T) from the sequence derived from ABQO010292497.1 to correct for the breaks in reading frame. The nucleotide sequence encoding the ten amino acid region YKIFLLQAYR is derived from EST entry [GenBank:EX196206.1]. Sizes of the missing regions are based on length of the corresponding regions of opossum TERT.

Sequences – Human AS TERT variant pΔ2(136-end)

This sequence is too short to be accepted by GenBank database.

```
LOCUS                                    55 bp    mRNA    linear   PRI 10-MAR-2011
DEFINITION  Homo sapiens telomerase reverse transcriptase isoform
            Delta2(136-end)(TERT) mRNA, partial sequence, alternatively
            spliced.
SOURCE      Homo sapiens (human)
  ORGANISM  Homo sapiens
            Eukaryota; Metazoa; Chordata; Craniata; Vertebrata; Euteleostomi;
            Mammalia; Eutheria; Euarchontoglires; Primates; Haplorrhini;
            Catarrhini; Hominidae; Homo.
REFERENCE   1  (bases 1 to 55)
  AUTHORS   Hrdlickova,R., Nehyba,J., Lim,S., Grutzner,F. and Bose,H.R. Jr.
  TITLE     Insights into the evolution of mammalian telomerase: Platypus TERT shares
            similarities with genes of birds and other reptiles and localizes on sex
            chromosomes
FEATURES             Location/Qualifiers
     source          1..55
                     /organism="Homo sapiens"
                     /mol_type="mRNA"
                     /cell_line="SAOS-2"
     gene            <1..>55
                     /gene="TERT"
     misc_feature    <1..>54
                     /gene="TERT"
                     /note="This variant is spliced using an alternative splice
                     donor site in exon 2. The variant lacks an internal
                     segment encoded by the 1219 nucleotides of the 3' portion
                     of exon 2. The open reading frame (<1..>55) that starts
                     with the ATG codon of the full-length TERT isoform
                     (cross-reference: NM_198253.2) ends in a premature
                     termination codon that is predicted to lead upon
                     translation to nonsense-mediated decay (NMD). This variant
                     is, therefore, represented as non-coding."
BASE COUNT        7 a     23 c     17 g      8 t
ORIGIN
        1 cccccсgagg ccttcaccac cagcgggttg gctgtgttcc ggccgcagag caccg
//
```
