# Supplementary material for: Insights into the evolution of mammalian telomerase: Platypus TERT shares similarities with genes of birds and other reptiles and localizes on sex chromosomes
Source: BMC Genomics. 2012 Jun 1;13:216. doi: 10.1186/1471-2164-13-216 (PMC3546421; doi:10.1186/1471-2164-13-216)
Supplement: Additional file 4 — Figure S2. Sequence alignment of platypus TERT with proteins of seven selected vertebrate species (PDF). The boundaries of the different domains and sequence motifs are indicated. This alignment serves as basis for the schematic representation of TERT proteins in Figure 1a. [file 1471-2164-13-216-S4.pdf]

```
CLUSTAL W 2.0 multiple sequence alignment

                                            TEN
                                                                                                     |------------- GQ ------------
HsaTERT    --------------------MPR-APRCRAVRSLLRSHYREVLPLATFVRRLGPQGW------------RLVQRGDPAAFRALVAQCLVCVPWDARPPPAAPSFRQVSCLKELVARVLQR
MmuTERT    --------------------MTR-APRCPAVRSLLRSRYREVWPLATFVRRLGPEGR------------RLVQPGDPKIYRTLVAQCLVCMHWGSQPPPADLSFHQVSSLKELVARVVQR
MdoTERT    ----------MASNPAVSGSLLA-SVAFRAVRSVLQARYRDVLGLAEFVQRLGDEAQAEGGSG---ADVQLLRGGEPEVFQVFVSQCVVCVPWDARPPPRPLTFQQLSSQKEVVARIVQR
OanTERT    --------------------MAS-AAPFFAVHAVLRARYAAVLPLPDFVGGLPGAPRGL---------LPLGDPGDPEIFQTFLAQCVVCLPRGARPLPDPLTFRQLSSQKEIVARIVQR
GgaTERT    ---MERGAQPGVGVRR-LRNVAR-EEPFAAVLGALRGCYAEATPLEAFVRRLQE---------GGTGEVEVLRGDDAQCYRTFVSQCVVCVPRGARAIPRPICFQQLSSQSEVITRIVQR
AcaTERT    MQKIEGAAFPALLPPRPRRGGPGQAMRRSQVCRLLRGCFEEVLPLEAFVKRLQEKEAKA----GGLPAEPLIQDGDPKCFRVLVERCLVGRPRGGKAPPPRLVFQQIFSQHDIIARVIRR
XleTERT    --------------------MPL-RTGGATLLSILQRLYGQVLGIVEYTDTLQVPGG---------IKVPVLLEGDSEKFRSFVAELVVCIPRGTKPLPSPVSFLQLSTQREVVARVIQR
TruTERT    ----------------------MSITDLSPTLGILRSLYPHVQVLVDFADDIVFREGHKAT---------LIEESDTSHFKSFVRGIFVCF---HKELQQVPSCNQICTLPELLAFVLNS
                                             *:  :  .  :  :.  :                  :   .:.  :: ::   .*      :         *:    :::: ::.

           ------------------------------------------------ GQ -------------------------------------------------------|
HsaTERT    LCERGAKNVLAFGFALLDGARGGPPEAFTTSVRSYLPNTVTDALRGSGAWGLLLRRVGDDVLVHLLARCALFVLVAPSCAYQVCGPPLYQLGAATQA-RPPPHASGPRR-----------
MmuTERT    LCERNERNVLAFGFELLNEARGGPPMAFTSSVRSYLPNTVIETLRVSGAWMLLLSRVGDDLLVYLLAHCALYLLVPPSCAYQVCGSPLYQICATTDIWPSVSASYRPTRPVGRNFT----
MdoTERT    ICEKKKKNILAFGYTLLEEKRMSLPVMFTTNVYNYHPNTITETISVSALWEMLLSRIGDDVMMYMLEHCSLFMMVPPSCCYQISGLPIYDLYLKDSTPPSGFVQRTYSKQGANASLDNVR
OanTERT    ICEKGKKNVLAFGYTLLDENSRSEPVVFTSNVCNYLPNISTESVRTSILWEMLFSRVGDDVIMYLLEHCALFMLVPPNCSYQICGQPIYELPPTDSSPPRPSSPRFFRQRASNRRRDVLS
GgaTERT    LCEKKKKNILAYGYSLLDENSCHFRVLPSSCIYSYLSNTVTETIRISGLWEILLSRIGDDVMMYLLEHCALFMLVPPSNCYQVCGQPIYELISRNVGPSPGFVRRRYSRFKHNSLLDYVR
AcaTERT    ICEKKKKNVLAFGYDLLDEN--HFPLPHMPNLYSYFPNNTTETICQSILWEKILNRVGDDFLMYILEHCSLFMLVPPSCCYQICGQPVYEIAFKDSTSFPKFLRQRYPGPKHSTLSGYLR
XleTERT    ICEKKRKNVLAFGYGLVDEKN-SLNIRLTPNICNYFPNPTTTTISTSILWETLLTRVGDDVMMYWLEQCSIFVFVPPRCCYQITGQPIYTLPSDDVFLFQSQSFTQSNVLLRYIKRNVFH
TruTERT    VKRKRKRNVLAHGYNFQSLAQEERDADQFKLQGDVTQS--AAYVHGSDLWRKVSMRLGTDITRYLFESCSVFVAVPPSCLFQVCGIPIYDCFSLATASLGFSLQSRG-------------
           : .:  :*:**.*: : .               .   .     :  *  *  :  *:* *.  : :  *:::: *.*   :*: * *:*


HsaTERT    -----------RLGCERAWNHSVREAGVPLGLPAPGARRRGGSASRSLP----------------------------LPKRPRRGAAPEPERTPVGQGSWAHPGRTR-------------
MmuTERT    ----------NLRFLQQIKSSSRQEAPKPLALPSRGTKRHLSLTSTSVP----------------------------SAKKARCYPVPRVEEGPHRQVLPTPSGKSW-------------
MdoTERT    -KISLLSKSLAKSNLRKEMLGSKEKAIKVVQQNQNSTEDPEDKSLRGRPEESC--DQGE-----QVRTHSVHSTMALLSKRQREDEEKSEISAKRSKTEELLQEKRKELILGQ-------
OanTERT    -EYVREKIRLHGRRGEQSDRKRRKRRVGERESGAGGRRETRLPSRRTNPGGRE----------------RDRPVTDGPRKRPDPGLAPRAAKRYLEKDERGTSGKRRKGAVLA-------
GgaTERT    KR--------LVFHRHYLSKSQWWKCRPRRRGRVSSRRKRRSHRIQSLRSGYQPSAKVNFQAGRQISTVTARLEKQSCSSLCLPARAPSLKRKRDGEQVEITAKRVKVMEKEI--EEQAC
AcaTERT    RRR-------FSSYKQHTARGNRKKWHPRRKLGSKANNILEGSYQQSLLIQTVKQNFTVS--------ASECPESKQRTSECKSLTTRSLKRKWKG-HYEMSAKRMKIMKIEDGLQKETG
XleTERT    LR--------KKYLKPKHSMTSRMLTWRRNKSPSGLLIRSKTSMAVTTEIHS-------------------------KRKLCSKDICVIPDKKRRDNLDKDDTVDHFDLPMCR-------
TruTERT    -------------CRERCLGVNSMKRRAFNVKR--YLRKRKTE----------------------------------TDQKD--EARVCSGKRRRV-----MEEDKVS------------
                                                                                          .


HsaTERT    -------------------GPSDRGFCVVSPA-RPAEEATSLEGALSGT-------------------------------------------------------------RHSHPSVGRQ
MmuTERT    -------------------VPSPARSPEVPT----AEKDLSSKGKVSDL-------------------------------------------------------------SLS--GSVCC
MdoTERT    -----------ELHENNESNLDNVESLTQRSMEICSSRPLFNEKSIHKEDGGGEGCFIKT---KGSLSLLEHKDGDLSRHNSSVIRSTLKGKAKAR-------------SSEGCLNRGAK
OanTERT    ---------------GRDGGPDPGSPSPATPTREVRPEAPGSEASEASRGLLQMQSVPGGRRDREDSRIPSDPPFPERVRGNLVRPGPRVPKTRGL-------------EAPSQPGSTGS
GgaTERT    SIVPDVNQSSSQRHGTSWHVAPRAVGLIKEHYISERSNSEMSGPSVVRRSHPGKRPVADKSSFPQGVQGNKRIKTGAEKRAESNRRGIEMYINPIHKPNRRGIERRINPTHKPELNSVQT
AcaTERT    NLVHTQSKHQLSLDGD-NAASKSSTSFCLADQLTPVTSVLHSNECGEQISGVHVAHLDHRKSFLS--SKTMTLVSGSKAHCEPSTKIDSVDR------------------STKQEGGIRS
XleTERT    --------------------SVSYLSNMYPKTNVQVTGLITSGYKKTKTFQCQKP------------------------------------------------------VSCEQKKTTAF
TruTERT    -------------------CETMQDGE--SGKTTLVQKQPG-----SKKRS----------------------------------------------------------------EMEAT
```

Figure S2

```
                                                      TRBD:     v-II                     |--- VSR --|                      v-III
HsaTERT    HHAGPPSTSRPPRPWDTPCPPVYAET-------------------------KHFLYSS-GDKEQLRPSFLLSSLRPSLTGARRLVETIFLG-------SRPWMPGTPRRLPRLPQRYWQM
MmuTERT    KHKPSSTSLLSPPRQNAFQLRPFIET-------------------------RHFLYSRGDGQERLNPSFLLSNLQPNLTGARRLVEIIFLG-------SRPRTSGPLCRTHRLSRRYWQM
MdoTERT    EADATFSHPLCPGNEPPKGLKSTSSSIIYIN-------------------RKRFLYSAWNFRECLPTSFLLNRLHDSLSGGQKLVETIFLTSHLFGQKGDPQQQRTPWKKRRLPKRYWQM
OanTERT    APPGWSSPARSPREEESPRSGAGEQTPGRGPPGSVPESSRAACRPGVFIDRKRLLYSSRDLKERLPRSFPLNRLRGDPAGGRGLVEAIFLAGSLREGRRDPAGRRLGRKKRRLPGRYWRM
GgaTERT    EPMEGASSGDRKQENPPAHLAKQLPNTLSRS--------------TVYFEKKFLLYS-RSYQEYFPKSFILSRLQGCQAGGRRLIETIFLSQNPLKEQQNQSLPQQKWRKKRLPKRYWQM
AcaTERT    TQMVSAKGATARRDFR---VHNSTSSEKSSD--------------NTSFKRYSLLYCHRQLHECLPNSFVLNKLKGSPSGGQSLVEIVFFTS-QIPKQLDSSNQSHSKRKKRLPKRYWQM
XleTERT    YSVAGDCNLSLKDNVNKLITNASVPTAQSRLSFSN-----------IFIDFGRTLYLSISYKKGFSESFILNSLDSTPSGSQKLVETIFLSN-FLAEQNFDQPKRDENCRYKLPKRYWKM
TruTERT    LLPLEGGPSWRSGTFPPLPPSQSFMR------------------------TLGFLYGGRGMRSFLLNRKKKTAEGFRKIQGRDLIRIVFFEG-------VLYLNGLERKPKKLPRRFFNM
                                                                 **     :. :      .        .: *:. :*:                     :*. *::.*

                  |------- CP -------|
HsaTERT    RPLFLELLGNHAQCPYGVLLKTHCPLRAAVT----------------------------PAAGVCAREKPQ-------------GSVAAPEEED--------------------------
MmuTERT    RPLFQQLLVNHAECQYVRLLRSHCRFRTANQ--------------------------------------------------------QVTDALN--------------------------
MdoTERT    RPLFKELIQNHRKCPYFILLKKNCPLRFSAAKADPSSQLTKPQAMASSHLTQAEGIQGETQDQGHQGEHTSRASNSME----EEPSSSLKDQISSKESSLSDAVSKKALPASHEVGK---
OanTERT    RHVFRELLQNHGRCPYGVLLRKSCPVRVGAAEPAAGTGPPGGPAGVSLRSAGRAGGRRTASPAGGSPEGSR-------------RAGAVSPTASP-------------------------
GgaTERT    REIFQKLVKNHEKCPYLVFLRKNCPVLLSEAC-LKKTELTLQAALP-----GEAKVHKHTEH-GKE-----------STEGTAPNSFLAPPSVLACGQ--------------PERGEQHP
AcaTERT    RGLFQELLQKHAKCPYLGILKRNCPIWISDSIRYGTEEQACEEKVN-----QERRHSSQESQEANTIEYCPSSVTVGLGHFPRTSGASSRSCGETELGQ-------------KVPEEQPI
XleTERT    KPHFQELIQNHKKFPYLVYLNKHCPVRSSMACSEKRS----------------------------------------------LQKNRIENDGKQ-----------------------LK
TruTERT    VPLFSQLLRQHRRCPYSRLLQKTCPLVGIKDAG---------------------------------------------------------------------------------------
              * :*: :* .  *   *.  * .

                     QFP                                                                                                 T
HsaTERT    --TDPRRLVQLLRQHSSPWQVYGFVRACLRRLVPPGLWGSRHNERRFLRNTKKFISL-GKHAKLSLQELTWKMSVRDCAWLRRSPG-VGCVPAAEHRLREEILAKFLHWLMSVYVVELLR
MmuTERT    --TSPPHLMDLLRLHSSPWQVYGFLRACLCKVVSASLWGTRHNERRFFKNLKKFISL-GKYGKLSLQELMWKMKVEDCHWLRSSPG-KDRVPAAEHRLRERILATFLFWLMDTYVVQLLR
MdoTERT    TDKGGGDILQLLRWHSSPWQVYVFLRECLHRLVPAELWGSTHNKCRFFKNVRKFLTL-GKHDKFSLRELMWKMRVKDCAWLALVRG-NHFIPASEHRLREEILAKFVYWLMNTYVVELLR
OanTERT    --GAGWGVHDLLRQHSSSWRVYGFVRECLLRVVPAELWGSSHNKCRFLRNVKAFLAL-GKFDKFSLRELRWKMRVSDCAWLRSGRG-DHSVPASEHRFREEILAKFLHWLMRAYVVELLR
GgaTERT    AEGSDPLLRELLRQHSSHWQVYGFVRECLERVIPAELWGSSHNKCRFFKNVKAFISM-GKYAKLSLQQLMWKMRVNDCVWLRLAKG-NHSVPAYEHCYREEILAKFLYWLMDSYVIELLK
AcaTERT    LDSSTSNFRGLLKQHSSHWQVYTFVRECLQRVVPAELWGSSYNKCRFYKNVKKFISL-GKLATFSMQELMWKMRVNDCTWLRLSKGPGHFVPASEHHFRQDLMSKFFYWLMDSYVTELLR
XleTERT    HFTTKANLLSLLKQHSSIWQVYMFVRECLNNVVPDIMWGSSHNKCRFFRNVKSFLFFSGKFGKISLSELMWSMRVEDCSWIRLQKS-DHFVPASEHLLREKILAKFVFWLMDTYVIQLLK
TruTERT    ----QAELSSFLPQHCGSHRVYLFVRECLLAVIPQELWGSEHNRLLYFARVRFFLRS-GKFERLSVAELMWKIKVNNCDWLK-ISKTG-RVPPSELSYRTQILGQFLAWLLDGFVVGLVR
                  .  :*  *..  :** *:* **  ::.  :**: :*.  :  . : *:   **   :*: :* *.: * :* *:         :*. *   *  ::. *. **:  :*  *::

                                                         RT             1         2                         3
HsaTERT    SFFYVTETTFQKNRLFFYRKSVWSKLQSIGIRQHLKRVQLRELSEAEVRQHREARPALLTSRLRFIPKPDGLRPIVNMDYVVGARTFRREKR---AERLTSRVKALFSVLNYERARRPGL
MmuTERT    SFFYITESTFQKNRLFFYRKSVWSKLQSIGVRQHLERVRLRELSQEEVRHHQDTWLAMPICRLRFIPKPNGLRPIVNMSYSMGTRALGRRKQ---AQHFTQRLKTLFSMLNYERTKHPHL
MdoTERT    SFFYITETMFQKNRLFFYRKCVWSKLQNIGIRKHFEKVHLKLLSEEEIKQNPEARFICLASRLRFVPKPTGLRPIVNVDSIIGARVSNKGTKDKKIRCFNGQLKNLFSVLNYERTLHPEV
OanTERT    AFFYVTETMFQKNLLFFYRKCVWNRLQAIGIRNHLAKVQLQAISEKEIKENLSKKYVPVVSKLRFIPKSNGLRPIVNADTIAGAKLFPKESRDKKVQYFNTQLKNLFSVLNYERTLHPHL
GgaTERT    SFFYITETMFQKNMLFYYRKFIWGKLQNIGIRDHFAKVHLRALSSEEMEVIRQKKYFPIASRLRFIPKMNGLRPVVRLSRVVEGQKLSKESREKKIQRYNTQLKNLFSVLNYERTVNTSI
AcaTERT    SFFYITETMFQKNLLFFFRKTVWSKLETIGLRNHLAKVHLHALSEEKIKNLQQEKYVPLASKLRFIPKTNGLRPVVRLDSVVGAKTFCEKIRERKVQLFKTQLKNLFSVLNYERIKNPAL
XleTERT    SFFYVTETMFQKHRLLFYRKSVWKKLQNIGLRKHLEKVKLRSLSSDELENMQQWKNVPLVSRLRFIPKTNGLRPISKISSTLSSQQ-SKENQEKKIHHFSSQIRNLFSVLNYEWNRNCSL
TruTERT    ACFYATESMGQKNAIRFYRQEVWAKLQDLAFRSHISKGQMVELTPDQVAALPKS---TIISRLRFIPKTDGMRPITRVIG-----------ADAKTRLYQSHVRDLLDMLRACVCSTPSL
           : ** **:  **: : ::*: :* :*: :..*.*: : ::  ::  ::    .       .:***:**  *:**: .                   .    ::: *:.:*.        :
```

Figure S2

```
                                         A                                                       IFD
HsaTERT       LGASVLGLDDIHRAWRTFVLRVRAQ-DPPPELYFVKVDVTGAYDTIPQDRLTEVIASIIKPQ--NT-YCVRRYAVVQKAAHGHVRKAFKSH--VSTLTDLQPYMRQFVAHLQET--SPLR
MmuTERT       MGSSVLGMNDIYRTWRAFVLRVRAL-DQTPRMYFVKADVTGAYDAIPQGKLVEVVANMIRHSE-ST-YCIRQYAVVRRDSQGQVHKSFRRQ--VTTLSDLQPYMGQFLKHLQDSDASALR
MdoTERT       LGSSLFGIDGIYKEWRQFVLRVKPSKDKISNFYFVKADVTGAYDTIPHEKLIEVISSIIKPKEKKV-YCIRRYAVVQKNAHGNIQKSFKRH--VSTYKDALPYMSQFVSHLQET--TSLQ
OanTERT       LGASMFGIDDIYKAWRQFVLRVLTSKDKERRFYFVKADVTGAYDTIPHDKLVEVISRILKPEDETV-YCIQRYAVIQKTAKGSVRKTFRRH--VSTLKDFPPSMKQFVSHLQET--TSLQ
GgaTERT       IGSSVFGRDDIYRKWKEFVTKVFESGGEMPHFYFVKGDVSRAFDTIPHKKLVEVISQVLKPESQTV-YGIRWYAVIMITPTGKARKLYKRH--VSTFEDFIPDMKQFVSKLQER--TSLR
AcaTERT       LGSSVFGKDDIFAAWKQFVLKILELNEEMPKFYFVKADVMGAYDSIPHDKLEEVVLQALSPNKKTITYSIRRYAVIIRTRNGLLRKYYRRH--ASTYKEFKPEMNHFVSHLQES--TSLR
XleTERT       IGSSVFGMDDIYKKWKKFVLDFEKPQVEKLQFYFVKTDVKGAYDTIPHSKLDEVISKVINPNANEV-YCIRRYATVSVDPTGRIIKSFKRH--VSELADVLPNMKQFVSNQQEK--NLLR
TruTERT       LGSTVWGMTDIHKVLSSIAPAQKEK---PQPLYFVKMDVSGAYESLPHNKLIEVINQVLTPVLNEV-FTIRRFAKIWADSHEGLKKAFIRQADFLEANMGSINMKQFLTSLQKK--GKLH
              :*::: *  .*.     :.            :**** **  *::::*: :* **:   :        : :: :* :         * :  :            * :*:   *.     *:

                                                 B'                                      C                 D
HsaTERT       DAVVIEQSSSLNEASSGLFDVFLRFMCHHAVRIRGKSYVQCQGIPQGSILSTLLCSLCYGDMENKLFAGIR-RDGLLLRLVDDFLLVTPHLTHAKTFLRTLVRGVPEYGCVVNLRKTVVN
MmuTERT       NSVVIEQSISMNESSSSLFDFFLHFLRHSVVKIGDRCYTQCQGIPQGSSLSTLLCSLCFGDMENKLFAEVQ-RDGLLLRFVDDFLLVTPHLDQAKTFLSTLVHGVPEYGCMINLQKTVVN
MdoTERT       NAIIVEQXXXXXXTSINLFAFFLHLIRNNILKIKNKYYVQCQGIPQGSILSTLLCSLCYGNMENKLLSGIQ-QDGLLIRLIDDFLLVTPHLTQAKVFLRTLAKGIPEYGCLINPKKTVVN
OanTERT       NAVVVQQSSSLNETSTSLFAFFRHMIQHNILKIRNRYYVQRRGIPQGSILSTLLCSLCYGDMENRLFPGIQ-QDGVLLRLIDDFLLVTPHLAQAKLFLRTLAKGIPEYGCFINPRKTVVN
GgaTERT       NAIVVEQCLTFNENSSTLFTFFLQMLHNNILEIGHRYYIQCSGIPQGSILSTLLCSLCYGDMENKLLCGIQ-KDGVLIRLIDDFLLVTPHLMQARTFLRTIAAGIPEYGFLINAKKTVVN
AcaTERT       NAVVVEQSISLKETSSHLSEFFSRLIRNSILKIKDSYYVQNCGIPQGSILSTLLCNMCYGDMENKLLRGIQ-KDGILMRLTDDFLLVTPHLTQAKTFLRTLAMGIPEYGFVINASKTVVN
XleTERT       NTILVEQNLLLNESSVKLLAVFQQIIRSHILRIKDRYYMQCCGIPQGSMLSTILCSLCYGDMENAMLGGIQ-KNGVLMRLIDDFLLVTPHLDQAKTFLRTLAEGIPQYGCSISPQKTVVN
TruTERT       HSVLVEQIFSSDLEGKDALQFFTQILKGSVIQFGKKTYRQCQGVPQGSAVSSVLCCLCYGHMENVLFKDIINKKSCLMRLVDDFLLITPNLHDAQTFLKILLAGVPQYGLVVNPQKVVVN
              .:::::*       .     .* :::    :.:    * *  *:**** :*::** :*:*.*** ::  :  :.. *:*: *****:**:* .*: **  :  *:*:**  :.  *.***

                                E          CT
HsaTERT       FPVEDEALGGTAFVQMPAHGLFPWCGLLLDTRTLEVQSDYSSYARTSIRASLTFNRGFKAGRNMRRKLFGVLRLKCHSLFLDLQVNSLQTVCTNIYKILLLQAYRFHACVLQLPFHQQVW
MmuTERT       FPVEPGTLGGAAPYQLPAHCLFPWCGLLLDTQTLEVFCDYSGYAQTSIKTSLTFQSVFKAGKTMRNKLLSVLRLKCHGLFLDLQVNSLQTVCINIYKIFLLQAYRFHACVIQLPFDQRVR
MdoTERT       FPVEEDILSDSDFTQLPAHCLFPWCGLLLDTRTLNVFCDYSNYSRTSIRTSLSFDHSIKAGRNMRNKLIAVLRMKCHGLFLDLQVNSLQTIYINVYKIFLLQAYRFHACALKLPFNQQVR
OanTERT       FHVDEDTVGCSHFTQLPAGCLFPWCGLLLDTRTLEVYCDYSTYSRTSIRASLSLCQSTSAGRSLRRKLLTVLKLKCHGLFLDVQVNSLRTVCINIYKIFLLQAYRFHACVLRLPFNQRVR
GgaTERT       FPVDD-IPGCSKFKHLPDCRLISWCGLLLDVQTLEVYCDYSSYAFTSIRSSLSFNSSRIAGKNMKCKLTAVLKLKCHPLLLDLKINSLQTVLINIYKIFLLQAYRFHACVLQLPFNQKVR
AcaTERT       FSVDEDIPGLSGFKQLPSHCMFPWCGLLIDTQTLEVYCDYSSYSCTSIRSSLSFNSSVKAGVSMRNKLLDVLKLKCHSLFVDLQINSLRTVCINVYKILLLQAYRFHACVLQLPFDQKIK
XleTERT       FPVDD-IPECSEVEQLPSHCLFRWCGLLLDTQTLDVYYDYSSYACTSIRSSMTFCHSSAAGKYMKQKLIRVLRLKCHSLFLDLKVNSLRTVCINTYKIFLLQAYRFHACVVQLPFGQRVM
TruTERT       FEDYGSTDSCPGLRVLPLRCLFPWCGLLLDTHTLDIYKDYSSYADLSLRYSLTLGSCHSAGHQMKRKLMGILRLKCHALFLDLKTNSLEAIYKNIYKLLLLHALRFHVCAQSLPFGQSVA
              *         .    :*   :: *****:*.:**::  *** *:  *:: *:::     **  :: **  :*::*** *::*:: ***.::  * **::**:* ***.*.  *** * :

HsaTERT       KNPTFFLRVISDTASLCYSILKAKNAGMSLGAKGAAGPLPSEAVQWLCHQAFLLKLTRHRVTYVPLLGSLRTAQTQLSRKLPGTTLTALEAAANPALPSDFKTILD
MmuTERT       KNLTFFLGIISSQASCCYAILKVKNPGMTLKAS---GSFPPEAAHWLCYQAFLLKLAAHSVIYKCLLGPLRTAQKLLCRKLPEATMTILKAAADPALSTDFQTILD
MdoTERT       KNPGFFLSIISDIASCCYSILKAKNPGVILGARGGSGPFPFEAARWLCYHAFLIKLVNHRIVYKCLLGTLKMSKLQLFRKIPKATMQMLRVASDPSLSQDFKMILD
OanTERT       KNPEFFLTVIADTASCCYSMVRARNAGVTLGAREASGPFPSEAAQWLCYQAFLTKLANHKVVYKCLLGPLKRSKQQLLRRLPGPTMLMLEAATDPMLCQDFKTILD
GgaTERT       NNPDFFLRIISDTASCCYFILKAKNPGVSLGSKDASGMFPFEAAEWLCYHAFIVKLSNHKVIYKCLLKPLKVYKMHLFGKIPRDTMELLKTVTEPSLCQDFKTILD
AcaTERT       SNPSFFLGIISQTASCCFCILKTKHSDIPFSAAGISSPLTYKAVQWLCYHAFSVKLAIHRVIYKCLLVPLAQSKKRLLQQMPEATVQLLKEVTEPSIYNDFKTILD
XleTERT       NNPPFFLTVISDMAPCFYTTFKAKNKDLTRGYKDVSCQFNFEAVQWLSYQAFLTKLHNHKVLYKCLIGPLQNCKMQLSRRLSQDTIELLKSVTDSSLHKDFSCIMD
TruTERT       KNPAYFLLMIWDMVEYTNYLIRLSNNGLISGSTSQTGSVQYEAVELLFCLSFLLVLSKHRRLYKDLLLHLHKRKRRLEQCLGDLRLARVRQAANPRNPLDFLAIKT
              .*  :** :* . .      .:  : .:          .  :*.. *   :*   *  *   *  *:  *   :  *   :    :  :. .::.    **  *
```

Figure S2

Figure S2 The ClustalX sequence alignment of platypus TERT with the TERT proteins of selected vertebrates. This alignment serves as basis for the schematic representation of TERT proteins in Figure 1. To maximize the information available for the alignment, all 25 vertebrate TERT proteins specified in Materials and Methods section were aligned, but only sequences of those proteins presented in Figure 1 are shown here. For the abbreviations of species names see the legend to the Figure 1. The boundaries of the different domains and sequence motifs are indicated. The color code for the domains is the same as in the Figure 1. The TEN domain is shown as defined by [1] except that we did not include the variable region corresponding to the first 12 amino acids of human TERT that is shortened and modified in some TERT proteins. Other regions are shown as defined in the following references [2](GQ, CP, QFP), [3](VSR), [4](v-II, v-III, A, B', C, D, and E), [5](motifs 1, 2), [6](motif 3), [7](IFD), and [8](CT).

1. Jacobs SA, Podell ER, Cech TR: **Crystal structure of the essential N-terminal domain of telomerase reverse transcriptase.** *Nat Struct Mol Biol* 2006, **13:**218-225.
2. Bosoy D, Peng Y, Mian IS, Lue NF: **Conserved N-terminal motifs of telomerase reverse transcriptase required for ribonucleoprotein assembly *in vivo*.** *J Biol Chem* 2003, **278:**3882-3890.
3. Moriarty TJ, Huard S, Dupuis S, Autexier C: **Functional multimerization of human telomerase requires an RNA interaction domain in the N terminus of the catalytic subunit.** *Mol Cell Biol* 2002, **22:**1253-1265.
4. Kuramoto M, Ohsumi K, Kishimoto T, Ishikawa F: **Identification and analyses of the *Xenopus* TERT gene that encodes the catalytic subunit of telomerase.** *Gene* 2001, **277:**101-110.
5. Li Y, Yates JA, Chen JJ-L: **Identification and characterization of sea squirt telomerase reverse transcriptase.** *Gene* 2007, **400:**16-24.
6. Xie M, Podlevsky JD, Qi X, Bley CJ, Chen JJ: **A novel motif in telomerase reverse transcriptase regulates telomere repeat addition rate and processivity.** *Nucleic Acids Res* 2010, **38:**1982-1996.
7. Lue NF, Lin YC, Mian IS: **A conserved telomerase motif within the catalytic domain of telomerase reverse transcriptase is specifically required for repeat addition processivity.** *Mol Cell Biol* 2003, **23:**8440-8449.
8. Huard S, Moriarty TJ, Autexier C: **The C terminus of the human telomerase reverse transcriptase is a determinant of enzyme processivity.** *Nucleic Acids Res* 2003, **31:**4059-4070.
