# Supplementary material for: Insights into the evolution of mammalian telomerase: Platypus TERT shares similarities with genes of birds and other reptiles and localizes on sex chromosomes
Source: BMC Genomics. 2012 Jun 1;13:216. doi: 10.1186/1471-2164-13-216 (PMC3546421; doi:10.1186/1471-2164-13-216)
Supplement: Additional file 5 — Figure S3. Sequence alignment of vertebrate TERT proteins (PDF). The ClustalX sequence alignment of TERT proteins used for the construction of the tree shown in the Figure 1d. [file 1471-2164-13-216-S5.pdf]

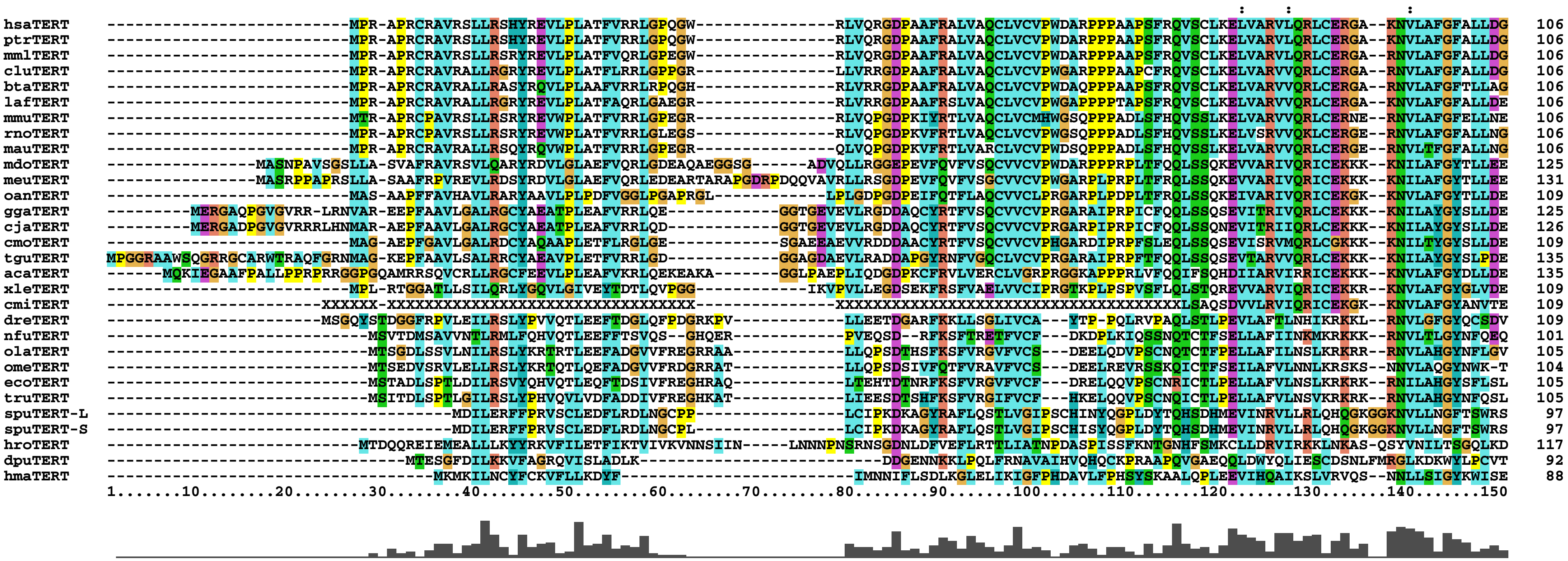
hsaTERT 106
ptrTERT 106
mmlTERT 106
cluTERT 106
btaTERT 106
lafTERT 106
mmuTERT 106
rnoTERT 106
mauTERT 106
mdoTERT 125
meuTERT 131
oanTERT 109
ggaTERT 125
cjaTERT 126
cmoTERT 109
tguTERT 135
acaTERT 135
xleTERT 109
cmiTERT 109
dreTERT 109
nfuTERT 101
olaTERT 105
omeTERT 104
ecoTERT 105
truTERT 105
spuTERT-L 97
spuTERT-S 97
hroTERT 117
dpuTERT 92
hmaTERT 88

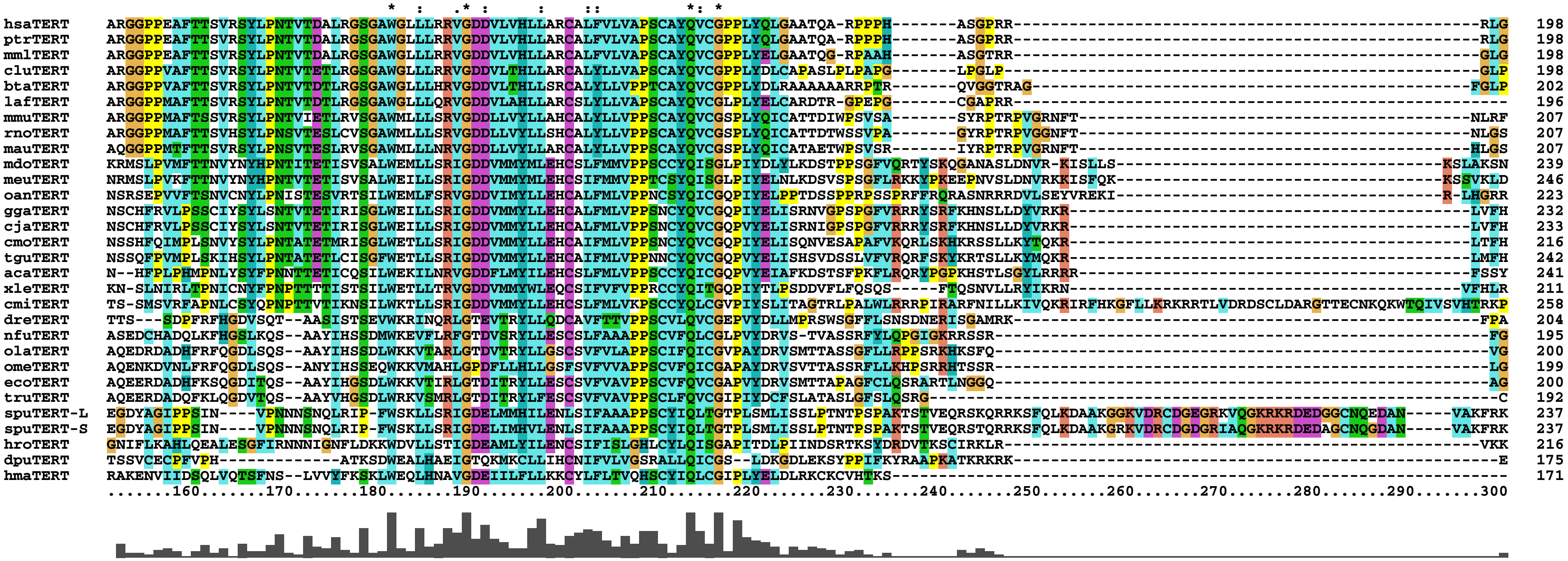
hsaTERT 198
ptrTERT 198
mmlTERT 198
cluTERT 198
btaTERT 202
lafTERT 196
mmuTERT 207
rnoTERT 207
mauTERT 207
mdoTERT 239
meuTERT 246
oanTERT 223
ggaTERT 232
cjaTERT 233
cmoTERT 216
tguTERT 242
acaTERT 241
xleTERT 211
cmiTERT 258
dreTERT 204
nfuTERT 195
olaTERT 200
omeTERT 199
ecoTERT 200
truTERT 192
spuTERT-L 237
spuTERT-S 237
hroTERT 216
dpuTERT 175
hmaTERT 171

Figure S3

```
hsaTERT    CERAWNHSVREAGVPLGLPAPGARRRGGSASRSLP----------------------------LPKRPRRGAAPEPERTPVGQGSWAHPGRTR---------------------------------GPSDRGFCVVSPA-RPAEEATSLE   286
ptrTERT    CERAWNHSVREAGVPLGLPAPGARRRGGSASRSLP----------------------------LPKRPRRGAAPEPERTPVGQGSWAHPGRTR---------------------------------GPSDRGFCVVSPA-RPAEEATSLE   286
mmlTERT    CELAWNRSVREAGVPMGLPAPGARRRRGSANRSLP----------------------------LPKRPRRGAAPEPERTPVGQGSWAHPDRTR---------------------------------GPSDRGFCVVSPA-RPAEEATSLE   286
cluTERT    GLGAGAGASADLRPTRQAQNSGARRRRGSPGSGVP----------------------------LAKRPRRSVAS----EPERGAHRSFPR--------------------------------------------AQQPP-VSEAPAVTP-   271
btaTERT    RPASSNGGHGEAEGLLEARAQGARRRRSSARGRLP----------------------------PAKRPRRGLEPG--RDLEGQVARSPPR--------------------------------------------VVTPT-RDAAEAKSRK   277
lafTERT    ------------------TVGGTRRRWDSAGERHP----------------------------LAKRSRHSLALEPGQVPQS---WAHLDRAC---------------------------------GQSDSDPHAVTPGRRAAAKAVSWE   263
mmuTERT    LQQIKSSSRQEAPKPLALPSRGTKRHLSLTSTSVP----------------------------SAKKARCYPVPRVEEGPHRQVLPTPSGKSW---------------------------------VPSPARSPEVPT----AEKDLSSK   292
rnoTERT    AHQIKNSGHQEAPKPQALPSRGTKRLLSLTSTNVP----------------------------SAKKARFEPALRVDKGPHRQVVPTPSGKTW---------------------------------APSPAASPKVPP----AAKNLSLK   292
mauTERT    THRVRNSSHQEAWKPPPLPSREAKRSLSITNRSVP----------------------------PSKKARCDLAPRLEKGPYRQAVPTPSDKTW---------------------------------VPNPAKSHAVPIS-RTTKEDLSSG   295
mdoTERT    LRKEMLGSKEKAIKVVQQNQNSTEDPEDKSLRGRPEESC--DQGE-----QVRTHSVHSTMALLSKRQREDEEKSEISAKRSKTEELLQEKRKELILGQ-------------------ELHENNESNLDNVESLTQRSMEICSSRPLFNE   363
meuTERT    VRKAILRSKDKTLKVAGQNQNSAEDMVEEPLEDRPENSCGIEQGSNADQLEEPAHSAHSMMALLSKRQRKDEEESEISAKRPKKEKCLLEKRKELILEQG-----------------FELHEN-ESNSDSAENITQTSMEICSSKHFCNE   378
oanTERT    GEQSDRKRRKRRVGERESGAGGRRETRLPSRRTNPGGRE----------------RDRPVTDGPRKRPDPGLAPRAAKRYLEKDERGTSGKRRKGAVLA-----------------------GRDGGPDPGSPSPATPTREVRPEAPGSE   334
ggaTERT    RHYLSKSQWWKCRPRRRGRVSSRRKRRSHRIQSLRSGYQPSAKVNFQAGRQISTVTARLEKQSCSSLCLPARAPSLKRKRDGEQVEITAKRVKVMEKEI--EEQACSIVPDVNQSSSQRHGTSWHVAPRAVGLIKEHYISERSNSEMSGP   380
cjaTERT    RHYLSKSQWWKCKPRHQGHVSSRRKR-SQRIQSLRPGYQSSEKVDFQAGRHISIVNAHMEKQSCSSLCLPNRAPSLKRKRDGEQVEIAAKRVKVMEKEI--EKQACSIIPDVNQSSSQRHRT-WHVAPRAVGLIKERYISERSNSEMSGP   379
cmoTERT    RQYLSKSRQSKRRQRLEANVSSMRNKTSNNIQSLG---------------------SAALEKQSSSNAGLSATAPSLKRKLAREQLEVTAKRARLEEKER--EEQACNTAPNVNQSIPKRYGT-SCVASRSVSLIKEKYISQRSNSDMSRP   343
tguTERT    RNYLLKSS----RQRYEENVSRMRNERKNKRRSLVPTDQSSAKTVSKGSNQIRMVTEDQEKQSSSSSCVSATALSLKRKIHREQFEIPAKKAKMGEKVR--EEKACSLVPNVNQSSSERSET-GYVAEHSESVIKTPCISERSNSAVSGP   385
acaTERT    KQHTARGNRKKWHPRRKLGSKANNILEGSYQQSLLIQTVKQNFTVS--------ASECPESKQRTSECKSLTTRSLKRKWKG-HYEMSAKRMKIMKIEDGLQKETGNLVHTQSKHQLSLDGD-NAASKSSTSFCLADQLTPVTSVLHSNE   381
xleTERT    KKYLKPKHSMTSRMLTWRRNKSPSGLLIRSKTSMAVT---------------------TEIHSKRKLCSKDICVIPDKKRRDNLDKDDTVDHFDLPMCR---------------------------SVSYLSNMYPKTNVQVTGLITSGY   313
cmiTERT    AKRAKLDTDIAKDTPRDVSSTGTKVTEVRPLKRLLHDEDKAESPAKKVKRDRSPEGAIPHSTPPEILSAGNKMNESPENVEVSDSTSIHSTRGGLVEQKVIDAKTGVGCQRKYPQGSTRERKRTWQEGTIAGYKRQAPGTDAKATHPTGA   408
dreTERT    VQKTVAISKKRTRDNEKYISVKRRRVKETVNNNNGNYRS-------------------LCFAISKKRAIDNEENISLKRRRMEETDQVAKIRNE--------------------------------NHESQSFAISKKRARDNEENISLK   303
nfuTERT    RNRTCHQLKRKPEIEDADLSKIRKQTVNK-----------------------------------RKRKREETVSCESKRRRVTHQEEGQTMSG---------------------------------------------------------   254
olaTERT    KKRRSANLTKRGSVGDVEESRKRRRVESGV---------------------------------SARKRKREPEEEESRERRRGVHHEERRQH-----------------------------------EAVLDES------TLSGKSGEND   276
omeTERT    KNRCSVKFKMRDSAEDEEKRRKKRRVESTN---------------------------------SSRKRKREPEEEESSKRRCDTHHEESSQHAC--------------------------------SEAVLVES------TVS----END   274
ecoTERT    RNRGAVTVRRKHKVENDKRNRRVKKRQRQ----------------------------------ADEKDEEEVMTCPGKRRRVGQHEHTQEVQVC--------------------------------SESVGEGQPTSVEPSLTLKTLENG   284
truTERT    RERCLGVNSMKRRAFNVKR--YLRKRKTE----------------------------------TDQKD--EARVCSGKRRRV-----MEEDKVS--------------------------------CETMQDGE--SGKTTLVQKQPG--   264
spuTERT-L  GEQGMRQPTQRKEAENREESKEDPKVLESMITGLERSYYILNACYSRTVGRTLLKHDLISKLPASNSGAQMLTQHIFNSPGKYDKTISHDGKGKGEIGMQ------GDDKGRKKKKLQRTCRRLIKVQALLKTFLTQHRKLKLNSILNHQ   381
spuTERT-S  GEQGMRQPTKKKEAENHDRTKKDPKVLENRITGLERSYYILNACYSRTVGRTLLKHNFISKLPASNSGAQMLTQHIFNSPGKSDKTASHDGKGKGEIGIQ------GDDKGRKRKKLQRTSRRLIKVQALLKTFLTQHRKLKLNSILNHQ   381
hroTERT    KHKPTKKSDNGFTKIVNAKSTSTRYGALKRSVDVTDAVEP-----------------------AKKKARCEERSEVNDGVVNVDDSLFSSSGDNNFNASN--------------------------TKNGSGMIDDEVNAPADDSSLFAS   317
dpuTERT    IENVVAILPKTSTLSVLDRPSVKRPLKKIRLASSD-----------------------------GQSQNVEFLVDTSKSIPPIVDKIPDKPPTS-------------------------------LVPVLKKIRFQQSDSLSLPIPLSIE   265
hmaTERT    VPNPVIRHQLIQANVADKNIRKRKMTFAS-----------------------------------SDFKRKKLEKTEFCEPLIFNKKVSKKACG---------------------------------------------------------   229
           .......310.......320.......330.......340.......350.......360.......370.......380.......390.......400.......410.......420.......430.......440.......450
```

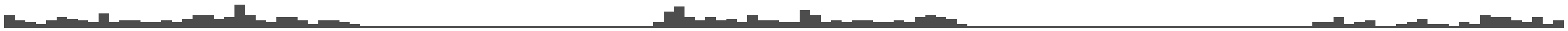

```
hsaTERT    GALSGT------------------------------------------------------------RHSHPSVGRQHHAGPPSTSRPPRPWDTPCPPV----------------------------------------------------   325
ptrTERT    GALSGT------------------------------------------------------------RHSHPSVGRQHHAGPPSTSRPPRPWDTPCPPV----------------------------------------------------   325
mmlTERT    GALSGT------------------------------------------------------------RHSHPSVGRQHHAGPPSTSRPPSPWDTRCPLV----------------------------------------------------   325
cluTERT    AVAASP------------------------------------------------------------AASWEGGPPGTRPTTPAWHPYPGPQGVPHDPA----------------------------------------------------   309
btaTERT    GDVPGP------------------------------------------------------------CRLFPGGERGVG--SASWRLSP-SEGEPGAGA----------------------------------------------------   313
lafTERT    GEIAGR------------------------------------------------------------RRSSTLGVKERGAGPSSAQPPL----SLLPQA----------------------------------------------------   298
mmuTERT    GKVSDL------------------------------------------------------------SLS--GSVCCKHKPSSTSLLSPPRQNAFQLRP----------------------------------------------------   329
rnoTERT    GKASDP------------------------------------------------------------SLS--GSVCCKHKPSSSSLLSSPPQDAEKLRP----------------------------------------------------   329
mauTERT    VKAPGL------------------------------------------------------------SRS--GSVCYKHKPSSTSLQSPLCQNAFQLRP----------------------------------------------------   332
mdoTERT    KSIHKEDGGGEGCFIKT---KGSLSLLEHKDGDLSRHNSSVIRSTLKGKAKAR------------SSEGCLNRGAKEADATFSHPLCPGNEPPKGLKSTSS-------------------------------------------------   450
meuTERT    KSALEEDGGREDCFIETKATKGGLSLLEHNEGDLSCHDTSIIKSTSEDIIEAK------------SSEEGLDGGTEQKDAIFTQPLCPGNEQPKRLRSASG-------------------------------------------------   468
oanTERT    ASEASRGLLQMQSVPGGRRDREDSRIPSDPPFPERVRGNLVRPGPRVPKTRGL------------EAPSQPGSTGSAPPGWSSPARSPREEESPRSGAGEQTPGRGPPG-----------------------------------------   432
ggaTERT    SVVRRSHPGKRPVADKSSFPQGVQGNKRIKTGAEKRAESNRRGIEMYINPIHKPNRRGIERRINPTHKPELNSVQTEPMEGASSGDRKQENPPAHLAKQLPN------------------------------------------------   483
cjaTERT    SVVHRSHPGKRPVADKSSFPQGVQGNKRVKTGAEKRVESNRRGTEMCINPTHKPNRRGVERRINPSHKPGLNSVQTEPMEGASSGDRKQENPPAHLAKQLPN------------------------------------------------   482
cmoTERT    SLVHNSHHGKKSVADKSSFLQGAESNRHLKPSIEMQAGSSRKRVEIHR------------------PIPRLDWIPIEPAESSSSGHKKQESPLAHLAEELPN------------------------------------------------   428
tguTERT    SLVHTSCGRRKSVA--VSLLQRFQSNKPLESNTEMQAESHRKRVEIRM------------------YESQLASGQTKPVKGSK--CRQQESPQPHLSKKLPN------------------------------------------------   466
acaTERT    CGEQISGVHVAHLDHRKSFLS--SKTMTLVSGSKAHCEPSTKIDSVDR------------------STKQEGGIRSTQMVSAKGATARRDFR---VHNSTSS------------------------------------------------   461
xleTERT    KKTKTFQCQKPVSCEQKK---------------------------------------------TTAFYSVAGDCNLSLKDNVNKLITNASVPTAQSRLSFSN------------------------------------------------   371
cmiTERT    GGVEVKRSWHFMGVQEAVSFGKSVNSESNEEKVLLEGRGSESKGKQAKVAKRKDESGKRCAKFAAGKDKRGTKRAASNEGDDNDHFNPGRSAKHGQSTKCIAKNDYSWDGLNPDGQCRAAVGVSGQAK----------------------   537
dreTERT    RQRMEEID---------------------------------------------------------------QVAKIRNENHGSQSWKPADQRPPRPSQ----------------------------------------------------   339
nfuTERT    -TAFKWPS---------------------------------------------------------------ATPAAIPSSEGSPSWRSGTFPPLPPSQ----------------------------------------------------   288
olaTERT    AAAVKPPP---------------------------------------------------------------ETSAAPPPLEGGPSWRSGAFPPLPSSQ----------------------------------------------------   312
omeTERT    KSSLKMPP---------------------------------------------------------------ETSAASLPLEGEPSWRSGTFPPLPSSQ----------------------------------------------------   310
ecoTERT    ATVSKQPA---------------------------------------------------------------ETQTTTLPLEGGPSWRSGMFPPLPPSQ----------------------------------------------------   320
truTERT    ---SKKRS---------------------------------------------------------------EMEATLLPLEGGPSWRSGTFPPLPPSQ----------------------------------------------------   296
spuTERT-L  CPLNLPQDGPPSDHTNNNHQEGSTGKQGQRSHTKKTSLSPPCAGEGASREKSLAELREESMLEGYSPKVFQRKRPKQSVLKNTQVSSVEQFDGTRMEDESVIQVDDGRGGVEQRRKDDGRKGSTKKVGEGIRDGLRDRVGLGGTEERVGN   531
spuTERT-S  CPLNHPQDSPPSDHTNNSHQECSTGTLGQRSHTKKTSLPPPSSGECASREKSLTELREESTLEGYSPKVFQRKRPKQSVINISQVSSVEQFDGTRMEER--VKIDEKNG------DSKSHPGSTKDS-----------------------   501
hroTERT    DNANKSQIDNNLNAASD-------------------------------------------------QNVKKRRRKRRKKNSSTVEESQDFRAYDAN------------------------------------------------------   366
dpuTERT    AKVPTN-------------------------------------------------------------ETEHPSSWKEMGKKKTRRWRNKLIAKQNVDNRR--------------------------------------------------   305
hmaTERT    ------------------------------------------------------------------------------------------------------------------------------------------------------   229
           .......460.......470.......480.......490.......500.......510.......520.......530.......540.......550.......560.......570.......580.......590.......600
```

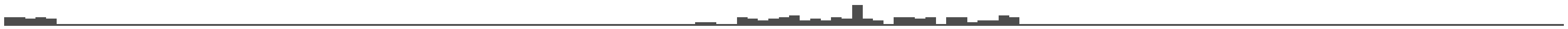

Figure S3

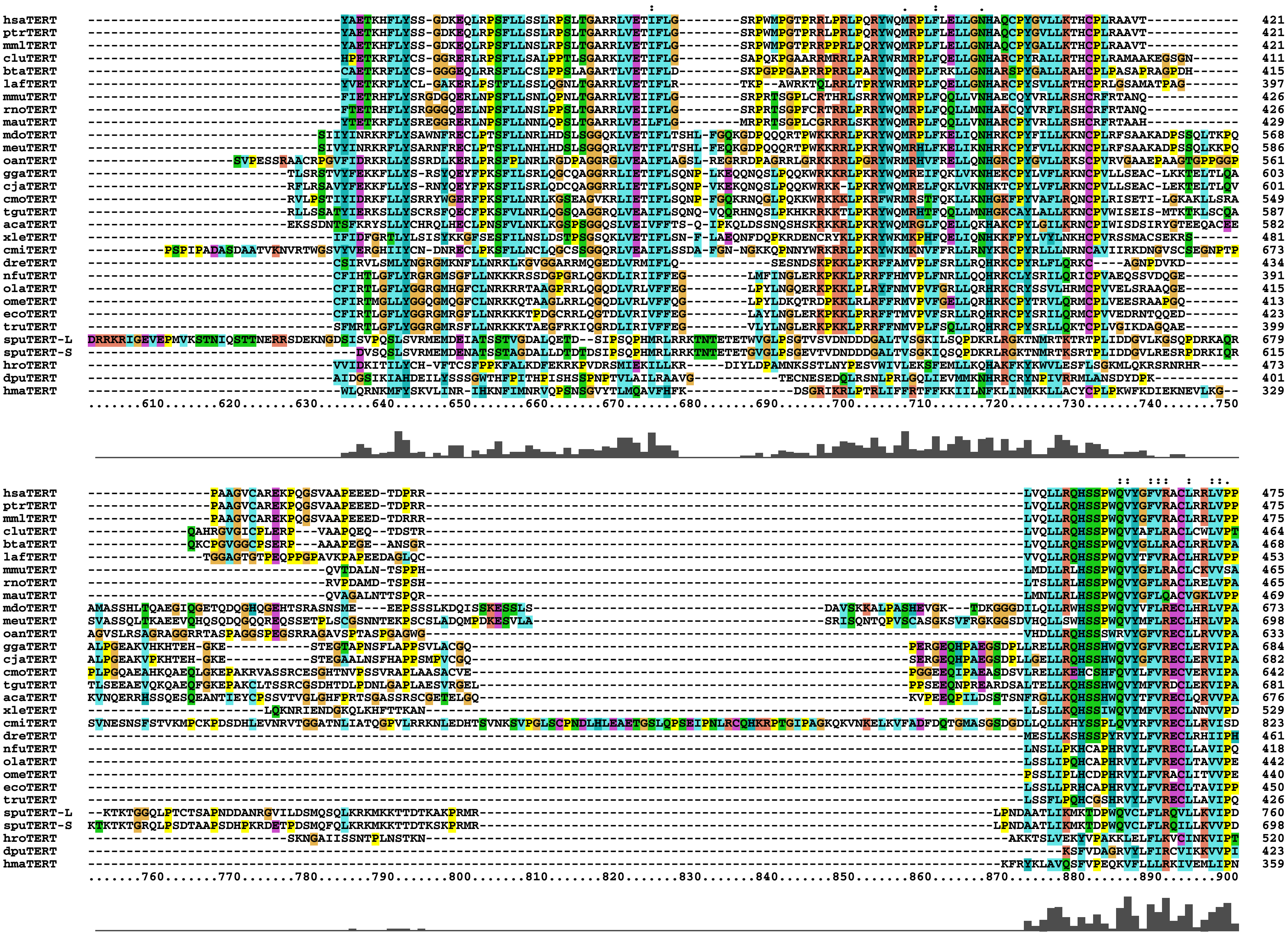
hsaTERT
ptrTERT
mmlTERT
cluTERT
btaTERT
lafTERT
mmuTERT
rnoTERT
mauTERT
mdoTERT
meuTERT
oanTERT
ggaTERT
cjaTERT
cmoTERT
tguTERT
acaTERT
xleTERT
cmiTERT
dreTERT
nfuTERT
olaTERT
omeTERT
ecoTERT
truTERT
spuTERT-L
spuTERT-S
hroTERT
dpuTERT
hmaTERT

Figure S3

```
           : *   *.  :        ::   *:   :   :   :    *                      :  ::      :    :   ::   *: *:     :  : ::    *  :     .               :
hsaTERT    GLWGSRHNERRFLRNTKKFISL-GKHAKLSLQELTWKMSVRDCAWLRRSPG-VGCVPAAEHRLREEILAKFLHWLMS-VYVVELLRSFFYVTETTFQKNRLFFYRKSVWSKLQSIGIRQHLKRVQLRELSEAEVRQH----REARPALLT   618
ptrTERT    GLWGSRHNERRFLRNTKKFISL-GKHAKLSLQELTWKMSVRDCAWLRRSPG-VGSVPAAEHRLREEILAKFLHWLMS-VYVVELLRSFFYVTETTFQKNRLFFYRKSVWSKLQSIGIRQHLKRVQLRELSEAEVRQH----QEARPALLT   618
mmlTERT    GLWGSRHNQRRFLRNTKKFISL-GKHAKLSLRELTWKMSVRDCAWLRKSPG-VGCVPAAEHRLREEILAKFLHWLMS-VYVVELLRSFFYVTETTFQKNRLFFYRKSVWSKLQSIGIRQHLKRVQLRELSEAEARQH----REARPTLLA   618
cluTERT    GLWGSRHNQRRFLRNVKKFISL-GKHAKLSLQELTWKMKVRDCTWLHGNPG-ACCVPAAEHRRREEILARFLVLVDGHIYVVKLLRSFFYVTETTFQKNRLFFYRKSVWSQLQSIGIRQLFNSVHLRELSEAEVRRH----REARPALLT   608
btaTERT    GLWGSRHNERRFLRNVKKLLSL-GKHGRLSQQELTWKMKVQDCAWLRASPG-ARCVPAAEHRQREAVLGRFLHWLMG-AYVVELLRSFFYVTETTFQKNRLFFFRKRIWSQLQRLGVRQHLDRVRLRELSEAEVRQH----QEARPALLT   611
lafTERT    GLWGSSHNKCRFLRNVKMFISL-GKHAKLSLQELMWKMKVQDCEWLRRSPG-DYAVPASEHRLREEILAKFLFWLMD-TFVVGLLRAFFYVTETMFQKNRLFFFRKSVWNKLQSIGIRQHFNKVRLRELSEAEVQRR----QQTKPSLLT   596
mmuTERT    SLWGTRHNERRFFKNLKKFISL-GKYGKLSLQELMWKMKVEDCHWLRSSPG-KDRVPAAEHRLRERILATFLFWLMD-TYVVQLLRSFFYITESTFQKNRLFFYRKSVWSKLQSIGVRQHLERVRLRELSQEEVRHH----QDTWLAMPI   608
rnoTERT    GLWGTRHNERRFLKNVKKFISL-GKYAKLSLQELMWRVKVEDCHWLRSSPE-KDTVPAAEHRLRERILAMFLFWLMD-TYVVQLLRSFFYITETTFQKNRLFFYRKSVWSKLQSIGIRQQLERVQLRELSQEEVKHH----QDTWLAMPI   608
mauTERT    GLWGSRHNQRRFFKNVKRFISL-GKYDKLSLQELTWKMKVQDCRWLRSSPG-NNCVPAAEHRTRERILAVFLFWLMD-AYVVELLRSFFYVTETTFQKNRLFFYRKSMWRRLQSIGVRHHLERVRLQELSQEEVRQR----QEAWPAMPI   612
mdoTERT    ELWGSTHNKCRFFKNVRKFLTL-GKHDKFSLRELMWKMRVKDCAWLALVRG-NHFIPASEHRLREEILAKFVYWLMN-TYVVELLRSFFYITETMFQKNRLFFYRKCVWSKLQNIGIRKHFEKVHLKLLSEEEIKQN----PEARFICLA   816
meuTERT    ELWGSTYNKCRFFKNVRKFLTL-GKHDKFSLRELMWKMRVKDCTWLALVKG-NHFVPASEHRLREEILAKFVYWLMD-TYVVELIRSFFYVTETMFQKNRLFFYRKCVWSKLQNIGIRKHFDKVQLRALSEEEIMQN----LETRFICLA   841
oanTERT    ELWGSSHNKCRFLRNVKAFLAL-GKFDKFSLRELRWKMRVSDCAWLRSGRG-DHSVPASEHRFREEILAKFLHWLMR-AYVVELLRAFFYVTETMFQKNLLFFYRKCVWNRLQAIGIRNHLAKVQLQAISEKEIKEN----LSKKYVPVV   776
ggaTERT    ELWGSSHNKCRFFKNVKAFISM-GKYAKLSLQQLMWKMRVNDCVWLRLAKG-NHSVPAYEHCYREEILAKFLYWLMD-SYVIELLKSFFYITETMFQKNMLFYYRKFIWGKLQNIGIRDHFAKVHLRALSSEEMEVI----RQKKYFPIA   827
cjaTERT    ELWGSSHNKCRFLKNVKAFISM-GKYDKLSLQQLMWKMRVNDCVWLRLAKG-NHSVPAYEHCYREEILAKFLYWLMD-SYVIELLKSFFYITETMFQKNTLFYYRKFIWGKLQNIGIRNHFAKVHLRALSSEEMDVI----RQKKYFPIA   825
cmoTERT    ELWGSNHNKRRFFKNVKAFISM-GKYAKLSLQVLMWKMRVNDCMWLRLAKG-NHFVPASEHRYREEILAKFLYWLMD-TYVVELLRSFFYITETMFQKNMLFYYRKCIWAKLQDIGIRKHFAKVQLRPLTAEEMEAI----HQKKYLPMA   785
tguTERT    ELWGSNHNKCRFLKNVKVFISR-GRFAKVSLQELMWRMRVNDCMWLRLGKG-DHFVPADEHCFREELLAKFLYWLMG-TYVVELLRSFFYITETMFQKNMLFYYRKFIWGKLQNIGIRNHFVKVQLRPLSSEEIETI----RQKKIVPVA   824
acaTERT    ELWGSSYNKCRFYKNVKKFISL-GKLATFSMQELMWKMRVNDCTWLRLSKGPGHFVPASEHHFRQDLMSKFFYWLMD-SYVTELLRSFFYITETMFQKNLLFFFRKTVWSKLETIGLRNHLAKVHLHALSEEKIKNL----QQEKYVPLA   820
xleTERT    IMWGSSHNKCRFFRNVKSFLFFSGKFGKISLSELMWSMRVEDCSWIRLQKS-DHFVPASEHLLREKILAKFVFWLMD-TYVIQLLKSFFYVTETMFQKHRLLFYRKSVWKKLQNIGLRKHLEKVKLRSLSSDELENM----QQWKNVPLV   673
cmiTERT    ELWGSNHNKYRFLKNVKKLISL-GKYDRFSLSELMWKMRVNDCTWLQLNNG-QCSVSPSEHRLREEILSKFLLWLMG-TYVVHLLKSFFYITETMFMKNMLFYYRKCVWNKIEKIGIRNHLAKAQLQPLSKEEWARK----QRQKTAIPF   966
dreTERT    ELWGCQENQLHFLSNVKNFLLL-GKFERLTLVQLMWRMKVQACHWLG-PKKRQ---CASEHRYREWMLGQCMGWMLS-GFVVGLVRAQFYITESMGHKHTLRFYRGDVWSRLQDQAFRAHLCKGQWRPLSPSQALKV----PNS---AVT   598
nfuTERT    QLWGSDQNRLHFFARVRDFLHS-GKFEKTSLAKVMWKVKVNDCDWLK-ISKTG-RVPPSELAYRTQILGQFLAWLLD-GYVVGLVRACFYATEGMRQKCCIRFYRREVWAKLQDLAFKDYLAQSQMKELCPAEVASF----PKD---TVI   557
olaTERT    ELWGSDHNRLQFFSRVRGFLKS-GKFERISVAELMWKIKVMDCDWLK-LRRTAGRFPPSELAYRTRILSQFLTWLLD-GFVVGLVRACFYATESVGQKNAIRFYRQEVWSKLQDLAFRRHIAKGEMEELSPAQVASL----PKG---TVI   582
omeTERT    ELWGSDHNRLQFFTKVRCFLKN-GKFERVSVAELMWKMKVMDCDWLK-LRRTG-RFPPSELTYRTQILSLFLTWLLD-GFVVGLVQACFYATDSVGQKSTIRFYRPEVWNKLRDLAFRNHVAKGQMEELTPDQVASL----PKD---AII   579
ecoTERT    ELWGSDHNRLHFFSRVRGFLRS-GKFERLSLAELMWKMKVNDCDWLK-ISKTG-RFPPSELSYRTQILGQFLAWLLD-GYVVSLVRACFYVTESVGQKNAVRFYRQEVWAKLQDLAFRGHLSKGQMEELTPAQVASL----PKT---TVI   589
truTERT    ELWGSEHNRLLYFARVRFFLRS-GKFERLSVAELMWKIKVNNCDWLK-ISKTG-RVPPSELSYRTQILGQFLAWLLD-GFVVGLVRACFYATESMGQKNAIRFYRQEVWAKLQDLAFRSHISKGQMVELTPDQVAAL----PKS---TII   565
spuTERT-L  ELWGSVHNRNVFLKGVKKFVQL-GRFERLSLRELTEEIRAEDCDWCQLEKFRGRDPPFTSVVKQRQLVTDFFHWLMV-GYAVPLIMMCFYVTETSSSRNQLIFYRKPVWLLLEKIGIQYYVAHGIMKPMKENDVKKL----VSAGLTLGF   904
spuTERT-S  ELWGSVHNRNVFLKGVKKFVQL-GRFERLSLRELTDDIRAEDCDWCQLEKFRGRNPPFTSLVKQRQLVTDFFHWLMI-GYTVPLIMMCFYVTETSSSRNQLVFYRKPAWLLLEKIGIQYYVAHGIMKPMKENDVRKL----VSAGLTLGF   842
hroTERT    ELLGSEHNKETFCKSVRYLLYS-GRFENLTLDQLMFKVKT-KCGWMSEIRN---------NSERLQLLARLHYWLMS-SYVMGLIRSYFFVTDSDIYKNKIFFYHKPVWKKICQKAISESQRRGLLKEISKELAESS----VSSGKSLGF   654
dpuTERT    ELLGGQTNQQIFLSNVRRFVEA-GRGTSFTLNDLIQSMQTSKCHWFSHFSS---------LPVQISLLARLITWLFV-GFVKPLIRQHFYATETSYGRNRIFFYSKDVWQRIHHRMLRSICSVSLRQPLPVLEKSTAPDPAAELQNLLLF   562
hmaTERT    NFWGALHNKKYFFKSLLKFLRM-GRYERCTVDYVMKNIKISLFNWTSSTNH------KQQCSKRFNVIRSMFLWLYN-DFLIPIVRAFFYVTETSSQKFCIFFYRKPVWKMITDIGMKS---SVVYKKLSVKDVTNK----LKTSECIGV   494
           .......910.......920.......930.......940.......950.......960.......970.......980.......990......1000......1010......1020......1030......1040......1050
```

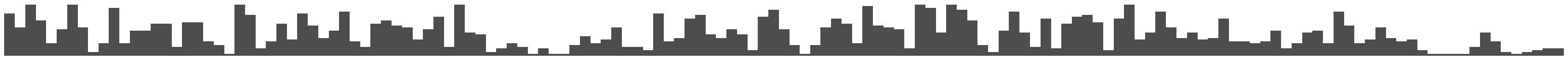

```
            :*: *     * :                                                                                *:  .: :: : ::  ::
hsaTERT    SRLRFIPKPDGLRPIVNMDYVVGARTFRREKR---AERLTSRVKALFSVLNYERARRPGLLGASVLGLDDIHRAWRTFVLRVRAQ-DPPPELYFVK-VDVTGAYDTIPQDRLTEVIASIIKPQ--NT-YCVRRYAVVQKAAHGHVRKAFK   760
ptrTERT    SRLRFIPKPDGLRPIVNMDYVVGARTFRREKR---AERLTSRVKALFSVLNYERARRPGLLGASVLGLDDIHRAWRTFVLRVRAQ-DPPPELYFVK-VDVTGAYDTIPQDRLTEVIASIIKPQ--NT-YXXXXXXXXXXXXXXXXXXXXX   760
mmlTERT    SRLRFLPKPDGLRPIVNMDYVVGARTFRREKR---AERLASRVKALFSVLNYERARRPGLLGASVLGLDDIHRAWRNFVLRVRAQ-DPPPKLYFVK-VDVMGAYDTIPQDRLTEVIASIIKPQ--NT-YCVRRYAVVRKAAHGHVRKTFK   760
cluTERT    SRLRFLPKPSGLRPIVNMDYIMGARTFHRDKK---VQHLTSQLKTLFSVLNYERARRPSLLGASMLGMDDIHRAWRTFVLRIRAQ-NPAPQLYFVK-VDVTGAYDALPQDRLVEVIANVIRPQE-ST-YCVRHYAVVQRTARGHVRKAFK   751
btaTERT    SRLRFVPKPGGLRPIVNVGCVEGAPAPPRDKK---VQHLSSRVKTLFAVLNYERARRPGLLGASVLGMDDIHRAWRAFVLPLRAR-GPAPPLYFVK-VDVVGAYDALPQDKLAEVIANVLQPQE-NT-YCVRHCAMVR-TARGRMRKSFK   753
lafTERT    SKLRFVPKAHGLRPIVNMDYVVGARTFRREKKR--LQHFNSQMKNLFSVLNYERAVRPGLLGASVLGIDDVYKAWRAFVQQVRAG-DPRPQLYFVK-VDVTGAYDAIPHNKLVEVIANVIKPKE-NI-YCIRRYAVVQKTAQGYIRKSFK   740
mmuTERT    CRLRFIPKPNGLRPIVNMSYSMGTRALGRRKQ---AQHFTQRLKTLFSMLNYERTKHPHLMGSSVLGMNDIYRTWRAFVLRVRAL-DQTPRMYFVK-ADVTGAYDAIPQGKLVEVVANMIRHSE-ST-YCIRQYAVVRRDSQGQVHKSFR   751
rnoTERT    CRLRFIPKLNGLRPIVNMSYGMDTRAFGKKKQ---TQCFTQSLKTLFSVLNYERTKHPNLMGASVLGTSDSYRIWRTFVLRVRAL-DQTPRMYFVK-ADVTGAYDAIPQDKLVEIVANIIRRSE-SM-YCIRQYAVVQKDSQGQVHKSFR   751
mauTERT    CRLRFIPKPSGLRPIVNMSY-MGTRAFDKGKQ---AQHFTQCLKTLFSVLNYELTKHTNLLGASVLGLNDIYRTWRTFVLRVRTL-DPAPRMYFVK-ADVTGAYDAIPQDKLVEVIANMIRHPD-NS-YCIHQYAVVQRDRQGQIHKSFR   754
mdoTERT    SRLRFVPKPTGLRPIVNVDSIIGARVSNKGTKDKKIRCFNGQLKNLFSVLNYERTLHPEVLGSSLFGIDGIYKEWRQFVLRVKPSKDKISNFYFVK-ADVTGAYDTIPHEKLIEVISSIIKPKEKKV-YCIRRYAVVQKNAHGNIQKSFK   964
meuTERT    SRLRFIPKPNGLRPIVNVDSIVGAKVSNKGTKNKKIRCFNGQLKNLFSVLNYERTLNPDILGSSLFGIDGIYKEWRQFVLRVRQSKDQVSNFYFVKVADLRGAYDTIPHDKLVEVISSIIKPEENKV-YCIRRYAVVQKNAHGNIQKAFK   990
oanTERT    SKLRFIPKSNGLRPIVNADTIAGAKLFPKESRDKKVQYFNTQLKNLFSVLNYERTLHPHLLGASMFGIDDIYKAWRQFVLRVLTSKDKERRFYFVK-ADVTGAYDTIPHDKLVEVISRILKPEDETV-YCIQRYAVIQKTAKGSVRKTFR   924
ggaTERT    SRLRFIPKMNGLRPVVRLSRVVEGQKLSKESREKKIQRYNTQLKNLFSVLNYERTVNTSIIGSSVFGRDDIYRKWKEFVTKVFESGGEMPHFYFVK-GDVSRAFDTIPHKKLVEVISQVLKPESQTV-YGIRWYAVIMITPTGKARKLYK   975
cjaTERT    SRLRFIPKINGLRPIVRLSRVVEGQKLSKESREKKIQRYNTQLKNLFSVLNYERTVNTSIIGSSVFGRDDIYRKWKEFVTKVFESGGEMPHYYFVK-GDVSRAFDTIPHKKLVEVISQVLKPESQTV-YGIRWYAVIMITPTGKARKLYK   973
cmoTERT    SKLRFIPKVSGLRPIVRMSGVVEAQTLSKESRAKKMNHYNMQLKNLFSVLNYERTINTSYIGSSVFGRDDIYKKWKTFVKKVLKSDGEIPHFYYVK-ADVSRAFDSIPHDKLVEVISQVLKPEKKTV-YCIRRYAVVMITGSGKTRKLYR   933
tguTERT    LKLRFIPKPNGLRPIVKVSGVVEPQALSKESREKKMNHYNTQLKNLFSVLNYERTINTSFIGSSVFGKDDIYKTWKQFVIKILESGGEIPHFYCVK-ADVSRAYDTIPHNKLVEVISRVLKPEKRTV-YCIRRYAVIMITPSGRAKRLYR   972
acaTERT    SKLRFIPKTNGLRPVVRLDSVVGAKTFCEKIRERKVQLFKTQLKNLFSVLNYERIKNPALLGSSVFGKDDIFAAWKQFVLKILELNEEMPKFYFVK-ADVMGAYDSIPHDKLEEVVLQALSPNKKTITYSIRRYAVIIRTRNGLLRKYYR   969
xleTERT    SRLRFIPKTNGLRPISKISSTLSSQQ-SKENQEKKIHHFSSQIRNLFSVLNYEWNRNCSLIGSSVFGMDDIYKKWKKFVLDFEKPQVEKLQFYFVK-TDVKGAYDTIPHSKLDEVISKVINPNANEV-YCIRRYATVSVDPTGRIIKSFK   820
cmiTERT    YALRFIPKRNGLRPIVKMRNITGSKKCVKGSNARKXXXXXXXXXXXXXXXXXXXXXXXXXXXXXXXXXXXXXXXXXXXXXXXXXXXXXXXXXXXXXXXX-ADVTGAYDTIPHAKLVDVISRVLDPKVQEN-YCIRRYASIWSNSAGQIRKSFK  1114
dreTERT    SRIRFIPKTSSMRPITRLSG-----------SRDTLQYFQSCVRVLQNVLSVCVREAPGPMGSTVWGWQDIHRRLQDFSPQQKSS---PRPLYFVK-VDVSGAYDSLPHLKLVEVLKEVLGPFAEQS-FFLRQYSSVWSDPTRGLRKRFC   732
nfuTERT    SRLRFIPKTDGMRPITRFIG-----------RDTKTKLYQAKARNFLDALQACVQSNPSLLGSTVWGLKDIHKVLSSLAPAQKDK---PQHLYFVK-VDVSSAYESLPHDKLMEVIGQVLSPVQEEL-FTVRCYSKIWMDPHEGLKKSFV   691
olaTERT    SQLRFIPKTDGMRPITRVIG-----------ADSNTRLHHKRIRDLMSMLQARVRSAPALLGSTVWGMTDIHKVLRSLAPAQKDK---PQPLYFVK-VDVSGAYDSLPHDKLKEVITEALSPVQEEV-FTVRHYAKIWADSHEGLKKAFA   716
omeTERT    SQLRFIPKADGMRPITRVIR-----------ADLHTRLHHWCIRDLMDLLQARVRSMPSLLGSTVWGLTDIHKVLSSLAPAQKAK---PQQLYFVK-VDVSGAYESLPHDKLKQVVSEALSPIQEQV-FAIRRYAKIWRDSHEGVKKTFT   713
ecoTERT    SRLRFIPKTDGMRPITRVVG-----------ADAKTRVYRGRVRDLLDMLRACVRSTPSLLGSTVWGMTDIHRVLRSQAPAQKDK---PQPLYFVK-VDVSGAYESLPHDKLIEVIGQALSPVQDEL-FTIRHYAKIWADSHEGLKKSFV   723
truTERT    SRLRFIPKTDGMRPITRVIG-----------ADAKTRLYQSHVRDLLDMLRACVCSTPSLLGSTVWGMTDIHKVLSSIAPAQKEK---PQPLYFVK-MDVSGAYESLPHNKLIEVINQVLTPVLNEV-FTIRRFAKIWADSHEGLKKAFI   699
spuTERT-L  SRLRFVPKTKSLRPITRMGKS--------VIEEKKGLSVRLLLQDLFDVLTYHKINQPSVMGSSLLGIDGIYHKVLKFIKDRKER-KDTRPLYFVK-IDIEKCYDSIKHSKLLQIISMLLQGHDKPDEYQLQRYVTVTRAASAPTGLQKR  1044
spuTERT-S  SRLRFVPKTKGLRPITRMGKS--------VIEEKKGLSVRLLLRDLFDVLTYHKLNQPSVMGSSLLGIDGIYHKVLKFMKDRKER-KDTRPLYFVK-IDIEKCYDSIKHSKLLQIISMLLQGHDKPDEYQLQRYVTVTRAASASTGLQKR   982
hroTERT    AKVRFTPKLSSVRMITNMKK----------KQNLNLRAINWQLDDVRHIILYERSLKPEIMGSSVLSINHTHNLWKKYFLN-NYTADGLTNFFFVK-TDIKSCFDSISHYKLYSILKRIFDEPK---IYQLIKYNQIKLGSHTPYKNTVR   789
dpuTERT    ARLRFVPKKNGSRPIMSTKFR-----------------NTSRKEVNTARLLLSTIAGLYAEGMDAKSTHSLHRKWVHFVENRDKE----QPVYFVN-ADVEDAFGSIDHQKMVALLKGYGRTLP----KELQIRTLCTVRPDGRRSKPFQ   686
hmaTERT    YDIRLIPGQEKVRPIANMNSS------WRGINLKEKFFINYELENIHLALRYECEQSPQLLGASVCESTKLFMKWKNFRSHIKMNYQENPNFYFAK-VDIGRCFDTLPQDHVLDLLSSVLKQKS----YLIRKYTRLKYSQNGISKKISK   633
           ......1060......1070......1080......1090......1100......1110......1120......1130......1140......1150......1160......1170......1180......1190......1200
```

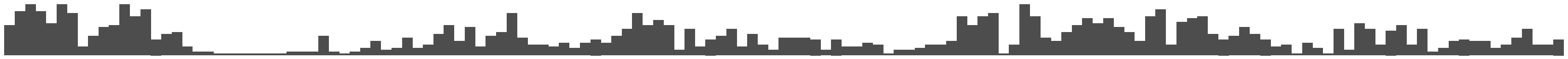

Figure S3

```
                                                                                            *:.*** ::. :  : :..:                                           :  ..  *.      *
hsaTERT     SH--VSTLTDLQPYMRQFVAHLQET--SPLRDAVVIEQSSSLNEASSGLFDVFLRFMCH-HAVRIRGKSYVQCQGIPQGSILSTLLCSLCYGDMENKLFAGIR--RDGLLLRLVDDFLLVTPHLTHAKTFLRTLVRGVPEYGCVVNLRKT   903
ptrTERT     XX--VSTLTDLQPYMRQFVAHLQET--SPLRDAVIIEQSSSLNEASSGLFDVFLRFVCR-HAVRIRGKSYVQCQGIPQGSILSTLLCSLCYGDMENKLFAGIR--RDGLLLRLVDDFLLVTPHLTHAKAFLRTLVRGVPEYGCVVNLRKT   903
mmlTERT     SH--VSTLTDLQPYMRQFVAHLQET--SPLRDAVVIEQSSSLNETSSGLFDVFLRFVCH-HAVRIGGKSYVQCQGIPQGSILSTLLCSLCYGDMENKLFAGMR--RDGLLLRLVDDFLLVTPHLTHAKAFLRTLVRGVPEYGCVVNLRKT   903
cluTERT     RH--VSTFADLQPYMRQFVERLQET--SLLRDAVVIEQSSSLNEAGSSLFHLFLRLVHN-HVVRIGGKSYIQCQGVPQGSILSTLLCSLCYGDMERRLFPGIE--QDGVLLRLVDDFLLVTPHLTQAQAFLRTLVKGVPEYGCRANLQKT   894
btaTERT     RH--VSTFSDFQPYLRQLVEHLQAM--GSLRDAVVIEQSCSLNEPGSSLFNLFLHLVRS-HVIRIGGRSYIQCQGIPQGSILSTLLCSFCYGDMENKLFPGVQ--QDGVLLRLVDDFLLVTPHLTRARDFLRTLVRGVPEYGCQVNLRKT   896
lafTERT     RHQ-VSTLADLLPYMKQFVEHLQET--SSLKNAVVIEQSSSLNEPASSLFDFFLHLVHN-NVIKIRGKYYVQCQGIPQGSILSTLLCSFCYGDMENKLFSGIQ--EDGLLLRLVDDFLLVTPHLTQAKAFLSTLVRGVPEYGCMINLRKT   884
mmuTERT     RQ--VTTLSDLQPYMGQFLKHLQDSDASALRNSVVIEQSISMNESSSSLFDFFLHFLRH-SVVKIGDRCYTQCQGIPQGSSLSTLLCSLCFGDMENKLFAEVQ--RDGLLLRFVDDFLLVTPHLDQAKTFLSTLVHGVPEYGCMINLQKT   896
rnoTERT     RQ--VSTLSDLQPYMGQFTKHLQDSDASALRNSVVIEQSISMNETGSSLLHFFLRFVRH-SVVKIDGRFYVQCQGIPQGSSLSTLLCSLCFGDMENKLFAEVQ--QDGLLLRFVDDFLLVTPHLAHAKAFLSTLVHGVPEYGCMINLQKT   896
mauTERT     RQ--VSTLSDLQPHMGQFLKHLQDSDTSALRNSVVIEQSLSLNEASSSLFDFFLRFVRN-SVVKIGGRCYVQCQGIPQGSSLSTLLCSLCFGDMENKLFAEVQ--QDGLLLRFVDDFLLVTPHLVQAEAFLRALVRGIPEYGCMINLQKT   899
mdoTERT     RH--VSTYKDALPYMSQFVSHLQET--TSLQNAIIVEQXXXXXXTSINLFAFFLHLIRN-NILKIKNKYYVQCQGIPQGSILSTLLCSLCYGNMENKLLSGIQ--QDGLLIRLIDDFLLVTPHLTQAKVFLRTLAKGIPEYGCLINPKKT  1107
meuTERT     RH--XXXXXXXXXXXXXXXXXXXXX--XXXXXXXXXXXSSSLNETSMNLFTFFLHLIHN-SILKIKNKLYVQCRGIPQGSVLSPLLCSLCYGNMENQLLPGIQ--QDGLLIRLIDDFLLVTPHLTQAKVFLRTLAKGIPEYGCLINPKKT  1133
oanTERT     RH--VSTLKDFPPSMKQFVSHLQET--TSLQNAVVVQQSSSLNETSTSLFAFFRHMIQH-NILKIRNRYYVQRRGIPQGSILSTLLCSLCYGDMENRLFPGIQ--QDGVLLRLIDDFLLVTPHLAQAKLFLRTLAKGIPEYGCFINPRKT  1067
ggaTERT     RH--VSTFEDFIPDMKQFVSKLQER--TSLRNAIVVEQCLTFNENSSTLFTFFLQMLHN-NILEIGHRYYIQCSGIPQGSILSTLLCSLCYGDMENKLLCGIQ--KDGVLIRLIDDFLLVTPHLMQARTFLRTIAAGIPEYGFLINAKKT  1118
cjaTERT     RH--VSTFEDFIPDMKQFVSKLQER--TSLRNAIVVEQCLTFNENSSTLFSFFLQMLHN-NILEIGHRYYMQCSGIPQGSILSTLLCSLCYGDMENKLLPGIQ--KDGVLIRLIDDFLLVTPHLMQARTFLRTIAAGIPEYGFVINAKKT  1116
cmoTERT     RH--VSTFKDFMPDMKQFVSRLHES--TSLRDAIIVEQSLTFNETSASLFNFFLQMLNN-NILEIERSYYLQCSGIPQGSLLSTLLCSLCYGDMENKLFSGVQ--KDGVLIRLIDDFLLVTPHLTHARTFLRTLAMGIPEYGFLINPKKT  1076
tguTERT     RH--VSTFKDFMPDMKQFVSQLQEN--ASLQNAIVVEQSLTFYETSSSLFNFFLQMIHN-IILEIRNRYYLQCCGIPQGSILSTLLCSLFYGDMENKWLPGIK--QDGVLIRLIDDFLLFTPHLMKARTFLRTLTTGIPEYGLLINPNKT  1115
acaTERT     RH--ASTYKEFKPEMNHFVSHLQES--TSLRNAVVVEQSISLKETSSHLSEFFSRLIRN-SILKIKDSYYVQNCGIPQGSILSTLLCNMCYGDMENKLLRGIQ--KDGILMRLTDDFLLVTPHLTQAKTFLRTLAMGIPEYGFVINASKT  1112
xleTERT     RH--VSELADVLPNMKQFVSNQQEK--NLLRNTILVEQNLLLNESSVKLLAVFQQIIRS-HILRIKDRYYMQCCGIPQGSMLSTILCSLCYGDMENAMLGGIQ--KNGVLMRLIDDFLLVTPHLDQAKTFLRTLAEGIPQYGCSISPQKT   963
cmiTERT     RQX-XXXXXXXXXXXXXXXXXXXXX---XXXXXXXXXXXXXXXXXXXXXXXXXXXXXXX-XXXXXXXXXYVQRRGIPQGSILSTLLCSLCYGDMENQXXXXXX--XXXXXXXXXXXXXXXXXXXXXXXXXXXMLTAGIPEYGCFIHPNKT  1257
dreTERT     TKAEMSEP----LNMKGFVVDEQVS--GRLHDAILVER-HSSEVRGGDVFQFFQKMLCS-YVIHYDQQMFRQVCGIPQGSSVSSLLCNLCYGHMEKALLKDIA--KGGCLMRLIDDFLLITPHLSKATEFLTTLLSGVPDYGCQINPQKV   872
nfuTERT     RQADFLDHDFRPTNMKGFLMSQQKS--GKIHSAVTVEQHFCSDYRGVETLQFFTQMVTS-SVVQYRKKFYRRCRGIPQGSIMSSLLCCLCYGHMERVLFKTMSA-TKGCLMRLVDDFLLITPDQRQAHTFLKTLLAGVPQYGLVVNPQKV   837
olaTERT     RQVDFSDGSMGSTSMKGFVMSLQKS--SKVHHAVLVEQAFGSNLRGKDALQFFTQMLTG-SVVQHGKKTYRQCRGIPQGSVVSSLLCCLCYGHMENVLFRDIK--NKGCLMRLVDDFLLITPDRNQAQSFLSILLAGVPQYGVVANPQKV   861
omeTERT     KQVDFSDGDLGSSTMKNFTMALQQI--SNAHHSVLVEQAFTSNIQGKDAVQVFMQMLTG-SIVQYENKTYRQCRGIPQGSVVSSLLCCLCYGHMERVLFRDLSK-NSGCLMRLVDDFLLITPDLNQAHTFLRIFLAGVPQYGVVANPQKV   859
ecoTERT     RQADFLEDNMGSTNMKGFVTSLQKR--GKVHHAILVEQHFSSDLHGREALQFFTQMLTG-SVVQFGKKTYRQCRGIPQGSVVSSLLCCLCYGHMENVLFKDITE-KKRCLMRLVDDFLLITPDLQEAQSFLRILLAGVPQYGLVVNPQKV   869
truTERT     RQADFLEANMGSINMKQFLTSLQKK--GKLHHSVLVEQIFSSDLEGKDALQFFTQILKG-SVIQFGKKTYRQCQGVPQGSAVSSVLCCLCYGHMENVLFKDIIN-KKSCLMRLVDDFLLITPNLHDAQTFLKILLAGVPQYGLVVNPQKV   845
spuTERT-L   SHR--HVTTELSSFHRQLIQMAQHG---KIKNAIIINQVHTVKVTPKDLLQRLKQHVMA-DVVKSGRKYYWRQDGISQGSILSSLLCSFFYAHLERCYLSDIV--QEGLMMRLIDDFLLITPHHDKAQRFLQLLLSGVKQYGCSANPSKT  1186
spuTERT-S   SHR--HVTTELSSFHRQLIQMAQHG---KIKNAIIINQVHTVKVTPKDLLQRLKQHVMA-DVVKSGRKYYWRQDGISQGSILSSLLCSFFYAHLERCYLSDID--QEGLMMRLIDDFLLITPHHDKAQRFLQLLLSGVKQYGCSANPSKT  1124
hroTERT     AA---VEKTVEPFNFKNYAQELVGK--KSCMNTIFIDTFNYWQISTEMVLQRLKAHLLG-NILQFNGSYYLQMQGISQGSCISSLLCNLYYADMEFKNFSVRS---DELLLRQVDDYIFVSKDVERAKIILEKMSAGFVEYSFQINKEKT   930
dpuTERT     VIP-----YFHKTNPEGWVQQMSPG------TVVFHSGTRTEKLDIVKILQFAIKSVREVHVVHHDKTHYRLKRGIPQGSQLSSALCHIYYGHLVREHLFEFLNHPEDLLIRVVDDFLYLTTNGERARAFHHRIHQGFTDYNAYVNINKS   825
hmaTERT     VATDGMECVTFIDFIRHGLKCGTIVG----NGCVFIDNVYTKLAYKTDLISLLKKHVKF-SIGKIGNTFLKQENGIPQGSVIAAIINDLHYGNIEQKHLNEFLASPDYLLMRWVDDYLFVATSRDTAISFIYKLHQSVAMYGASINMKKF   778
            ......1210......1220......1230......1240......1250......1260......1270......1280......1290......1300......1310......1320......1330......1340......1350
```

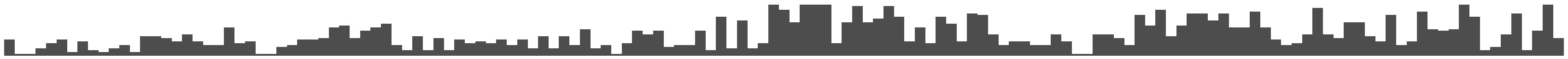

```
             *:                    : :**  :. :.*::  ::  :   :   :       .   :  :*      *   :  .*            .    :  .  :*
hsaTERT     VVNFPVEDEALGGTAFVQMPAHGLFPWCGLLLDTRTLEVQSDYSSYARTSIRASLTFNRG-FKAGRNMRRKLFGVLRLKCHSLFLDLQVNSLQTVCTNIYKILLLQAYRFHACVLQLPF----HQQVWKNPTFFLRVISDTASLCYSILK  1048
ptrTERT     VVNFPVEDEALGGTAFVQLPAHGLFPWCGLLLDTRTLEVQSDYSSYARTSIRASLTFNRG-FKAGRNMRRKLFGVLRLKCHSLFLDLQVNSLQTVCTNIYKILLLQAYRFHACVLQLPF----HQQVWKNPTFFLRIISDTASLCYSILK  1048
mmlTERT     VVNFPVEDEALGGAAFVQLPAHGLFPWCGLLLDTRTLEVQSDYSSYARTSIRASLTFNRG-FKAGRNMRRKLFGVLRLKCHSLFLDLQVNSLQTVCTNVYKILLLQAYRFHACVLQLPF----HQQVWKNPAFFLRVISDTASLCYSILK  1048
cluTERT     AVNFPVEDGALGSAAPLQLPAHCLFPWCGLLLDTRTLEVSCDYSSYAHTSIRASLTFSQG-AKPGRNMRRKLLAVLRLKCCALFLDLQVNGIHTVYMNVYKIFLLQAYRFHACVLQLPF----NQPVRKNPSFFLRVIADTASCCYSLLK  1039
btaTERT     VVNFPVEPGALGGAAPLQLPAHCLFPWCGLLLDTRTLEVHGDHSSYARTSIRASLTFTQG-FKPGRNMRRKLLAVLQLKCHGLFLDLQVNSLQTVFTNVYKIFLLQAYRFHACVLQLPF----SQPVRSSPAFFLQVIADTASRGYALLK  1041
lafTERT     VVNFPVDDGP-GQATILQLPAHCLFPWCGLLLDTRTLEVFCDYSSYARTSIKASLVFNHG-FKAGRNMRRKLFAVLRLKCHGLFLDLQVNSLQTVYINVYKIFLLQAYRFHACVLQLPF----NQQVGKNPSFFLRVISDTALRCYSILK  1028
mmuTERT     VVNFPVEPGTLGGAAPYQLPAHCLFPWCGLLLDTQTLEVFCDYSGYAQTSIKTSLTFQSV-FKAGKTMRNKLLSVLRLKCHGLFLDLQVNSLQTVCINIYKIFLLQAYRFHACVIQLPF----DQRVRKNLTFFLGIISSQASCCYAILK  1041
rnoTERT     VVNFPVETGALGGAAPHQLPAHCLFPWCGLLLDTRTLEVFCDYSGYGRTSIKMSLTFQGV-SRAGKTMRYKLLSVLRLKCHGLFLDLQVNSLQTVCINIYKIFLLQAYRFHACVIRLPF----GQHVRKNHAFFLGIISNLASCCYAILK  1041
mauTERT     VVNFPVDAGTLDGTAPHQLPAHCLFPWCGLLLDTQTLEVLCDYTGYARTSIKASLTFQRT-FKAGRNMRQKLLAVLRLKCHSLFLDLQMNSLQTVCINVYKIFLLQAYRFHACALQLPF----DQHVRKNPAFFLSIISNIASCCYSILK  1044
mdoTERT     VVNFPVEEDILSDSDFTQLPAHCLFPWCGLLLDTRTLNVFCDYSNYSRTSIRTSLSFDHS-IKAGRNMRNKLIAVLRMKCHGLFLDLQVNSLQTIYINVYKIFLLQAYRFHACALKLPF----NQQVRKNPGFFLSIISDIASCCYSILK  1252
meuTERT     VVNFPVEEDILSDSDFTQLPAHCLFPWCGLLLDTRTLNVFCDYSNYSRTSIRASLSFDHS-VKAGRNMRNKLIAVLRLKCHGLFLDLQXXXXXXXXXXXYKIFLLQAYRFHACVLKLPF----NQQVRKNPGFFLSVISDIASCCYSILK  1278
oanTERT     VVNFHVDEDTVGCSHFTQLPAGCLFPWCGLLLDTRTLEVYCDYSTYSRTSIRASLSLCQS-TSAGRSLRRKLLTVLKLKCHGLFLDVQVNSLRTVCINIYKIFLLQAYRFHACVLRLPF----NQRVRKNPEFFLTVIADTASCCYSMVR  1212
ggaTERT     VVNFPVDD-IPGCSKFKHLPDCRLISWCGLLLDVQTLEVYCDYSSYAFTSIRSSLSFNSS-RIAGKNMKCKLTAVLKLKCHPLLLDLKINSLQTVLINIYKIFLLQAYRFHACVLQLPF----NQKVRNNPDFFLRIISDTASCCYFILK  1262
cjaTERT     VVNFPVDD-IPGCSKFKQLPDCRLISWCGLLLDVQTLEVYCDYSSYAFTSIRSSLSFNSS-RIAGKNMKCKLTAVLKLKCHPLLLDLKINSLKTVLINIYKIFLLQAYRFHACVLQLPF----NQKVRNNPYFFLRIISDTASCCYYILK  1260
cmoTERT     VVNFSVDD-IPECSEFKQLPNCRLIPWCGLLLDTQTLEVYCDYSSYSCTSIRSSLSFNSN-RTAGKNMKHKLVAVLKLKCHGLFLDLQINSVKTVFINVYKIFLLQAYRFHACVIQLPF----NQKVRNNPDFFLRVIAENASCCYSMLK  1220
tguTERT     VVNFPVDD-IPGCSKFKQLPDCRLIPWCGLLLDIKTLEVYCDYSSYTCTSIRSSLSFNSS-VTAGKNMKYKLSAVLKLKCHSLFLDLQINSLRTVLINIYKIFLLQAYRFHACVLQLPF----NQQVRKNPHFFLRIISQTASCCYAILK  1259
acaTERT     VVNFSVDEDIPGLSGFKQLPSHCMFPWCGLLIDTQTLEVYCDYSSYSCTSIRSSLSFNSS-VKAGVSMRNKLLDVLKLKCHSLFVDLQINSLRTVCINVYKILLLQAYRFHACVLQLPF----DQKIKSNPSFFLGIISQTASCCFCILK  1257
xleTERT     VVNFPVDD-IPECSEVEQLPSHCLFRWCGLLLDTQTLDVYYDYSSYACTSIRSSMTFCHS-SAAGKYMKQKLIRVLRLKCHSLFLDLKVNSLRTVCINTYKIFLLQAYRFHACVVQLPF----GQRVMNNPPFFLTVISDMAPCFYTTFK  1107
cmiTERT     MVNFPLDNDLLGCINVKTLQEHCLFPWCGLLLNTQTLEVYCDYSSYANTSITSSLTFNCS-SKAGQNLRQKLLAVLKLKCHQIFLDLEVSTLRTVSINVYKIFLLQAYRFHACVMRFPF----GQRVRDNPSFFLSVISDMASCCYSILK  1402
dreTERT     AVNFPVCVS-WVNSGVSVLPSSCLFPWCGLMIHTHTLDVYKDYSRYDGLSLRYSLTLGSA-HSPSTVMK-KLLSVLSIKSTDIFLDLRLNSVEAVYRSLYKLILLQALRFHACVRSLPL----GQSVNRNPSFFLKMIWRMTRVTNKLLT  1015
nfuTERT     VVNFPIPERPWSGFDVHVLPSHCLFPWCGLLLDTQSLDVCKDYSRYSGLSLRYCMTLGSF-HSAGLQMRTKLMSILRLKSHTLFLDLKNNSIEVVYRNIYSLLLLQAYRFHACAQNLPF----GQTVAKNPVYFLQMIWDMAGFANRLIR   982
olaTERT     VVNFQGSEGGGAFPDIRVLPPHCLFPWCGLLLDTRSLDVCKDYSSYAGLSLRYSLTLGSA-HSAGQQMRRKLMSILRIKCHPLFLDLKTNSLESAYKNIHKLVLLQACRFHVCVQSLPF----AQTVAKNPTYFQQMIWDMAHYANALIR  1006
omeTERT     VVNFQPSEFGDAFTDVRVLPSHCLFPWCGLLLDTQSLDVYKDYSSYAGLSLRYSLTLGST-HLPGQQMRRKLMSILRLKCHPLFLDLKMNSHESVYKNIYKLVLLHACRFHVCAQSLPF----SQTVAKNPSYFLQMIWDMVRYANTLIR  1004
ecoTERT     AVNFQMLGSVGSCPGIRVLPPHCLFPWCGLLLDTHSLDVYKDYSSYAGLSLRYSLTLGSF-HSAGQQMRRKLMAILRLKCHALFLDLKTNSLEAVYKNIYKLVLLHARRFHVCVQSLPF----GQTVAKNPMFFLQMILDMAEYANQLIR  1014
truTERT     VVNFEDYGSTDSCPGLRVLPLRCLFPWCGLLLDTHTLDIYKDYSSYADLSLRYSLTLGSC-HSAGHQMKRKLMGILRLKCHALFLDLKTNSLEAIYKNIYKLLLLHALRFHVCAQSLPF----GQSVAKNPAYFLLMIWDMVEYTNYLIR   990
spuTERT-L   LANFDFMHDGQLVPRSKE-----LFPWCGIVFKTQTLNISNDYTKYNNVSIRYTLTICTDRDTSLHLMRIKLTWSLKAKNVSIFMDPLINSFLTIVINVYHLLLLLAHRFHSYYHCLAS----YIKQNTPPLQFYNIWASCVRIFHACTV  1327
spuTERT-S   LANFDFMYDGQLVPRSKE-----LFPWCGIVFKTQTLNISNDYTKYNNVSIRYTLTICTDRDTSLHLMRIKLTWSLKAKNVSIFMDPLINSFLTIVINVYHLPLLLAHRFHSYYHCLAS----YIKQNTPPLQFYNIWASCVRIFHACTV  1265
hroTERT     LTNFKYDAKNRGMCSGTFARSR--LPWCGLIIDMKTLEIYSDYSKFAGLSVKYTMSASFS-NHPGSAIEKKLLFSVRSKCNSIYFDTTYNSVNTIFLNVYKIFLLLAYKFVYLIRKLPM----QNSLEKNKVFFFTVIQNVCIQVTSLLR  1073
dpuTERT     ATNLDLTE-PRDETKDKPVRKPVWFSFCGLQFHTKTLEIRGDYTKYEGTDIIHCITPTSG--EPGKLLLKRLKGISTLKIETIYLDARVNRKPTITRSMFNACLMAAFRFHAIVKHVLMRSTRRHHWCDKALDARYLAHTVIKECCSKMV   972
hmaTERT     EINFNMQFEGLSIKSIDG----LFFPWCGYFIHQHNLELMYNYMQYIGEHIKDSITIEHN-FNPGLSMSRKLLQAVSVKATTLVLDTIFNSNDTIIILNIYQIFRFIGIKFYVHVKYGDG----NYLIDFTD---------------------   901
            ......1360......1370......1380......1390......1400......1410......1420......1430......1440......1450......1460......1470......1480......1490......1500
```

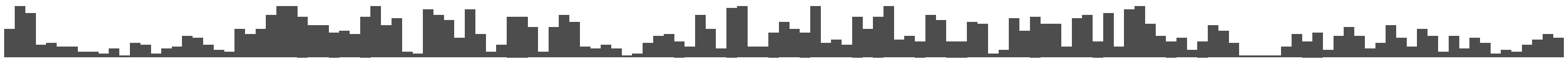

Figure S3

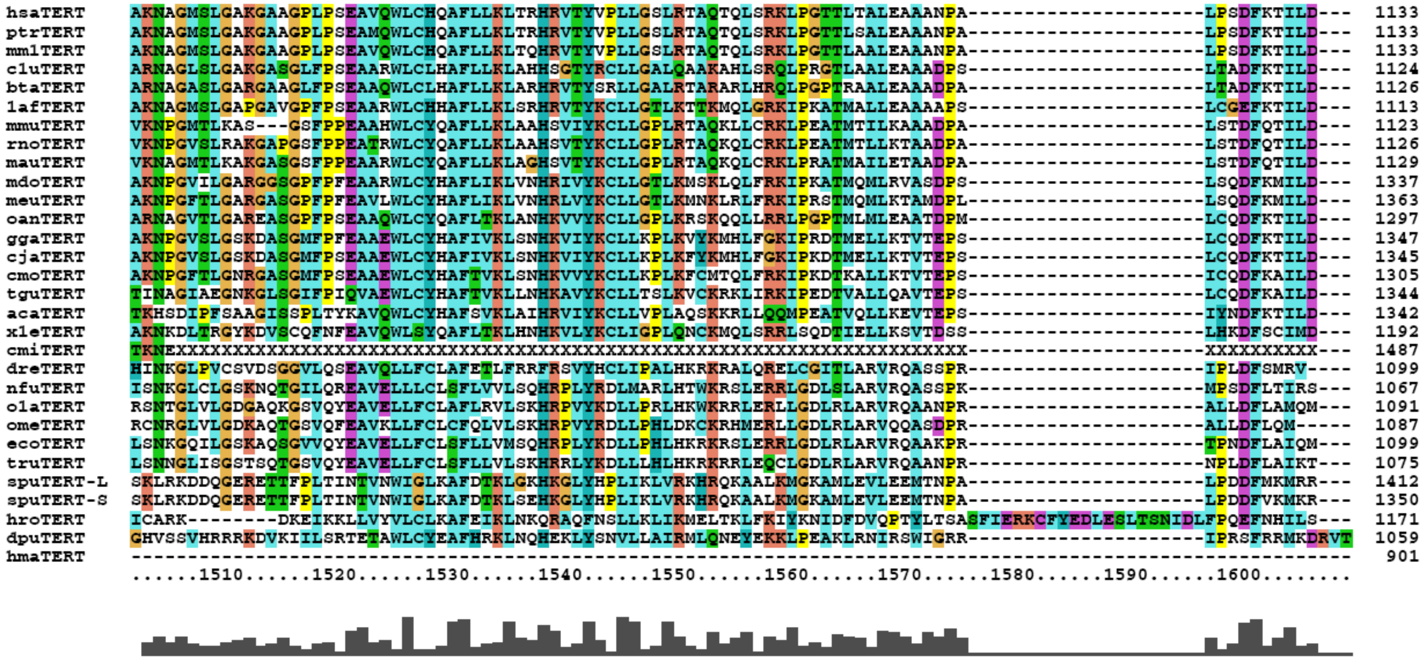

Figure S3 ClustalX sequence alignment of TERT proteins. The alignment was used for the construction of the the tree shown in the Figure 2. For the abbreviations of species names see the legend to the Figure 1.
