# Supplementary material for: Insights into the evolution of mammalian telomerase: Platypus TERT shares similarities with genes of birds and other reptiles and localizes on sex chromosomes
Source: BMC Genomics. 2012 Jun 1;13:216. doi: 10.1186/1471-2164-13-216 (PMC3546421; doi:10.1186/1471-2164-13-216)
Supplement: Additional file 6 — Table S2. Synteny analysis (PDF). Conserved synteny among human chromosome 5, platypus chromosomes X2 and X3 and chicken chromosome 2. [file 1471-2164-13-216-S6.pdf]

TABLE S2. Conserved synteny among human chromosome 5, platypus chromosomes X2 and X3, and chicken chromosome 2

Human gene [a]	5d [b]	Human location	Platypus chromosome [c]	Platypus contig	Chicken location
PLEKHG4B	✓	5:140373-190085	X2	Ultra474	No orthologs
LRRC14B	✓	5:191626-195468	X2	Ultra474	2:56936272-56939758
CCDC127	✓	5:204872-218330	X2	Ultra474	2:87982791-87987029
SDHA	✓	5:218356-256815	X2	Ultra474	2:87966100-87981950
PDCD6	✓	5:271736-353971	X2	Ultra474	2:91241065-91247539
AHRR	✓	5:304291-438406	X2	Ultra474	2:91291773-91357661
C5orf55	✓	5:441645-443258	GENE NOT FOUND		No orthologs
EXOC3	✓	5:443273-472052	X2	Ultra474	2:91222769-91240806
SLC9A3	✓	5:473425-524447	?	447	2:56883843-56919270
CEP72	✓	5:612387-667283	?	1382	No orthologs
TPPP	✓	5:660883-693510	?	1382	2:56762874-56792551
ZDHHC11	✓	5:710471-851101	?	1382	No orthologs
ZDHHC11B	✓	5:710475-767067	GENE NOT FOUND		No orthologs
BRD9	✓	5:850406-892939	?	3845	2:56688507-56710641
TRIP13	✓	5:892758-919472	X3	Ultra43	2:56673157-56688338
NKD2	✓	5:1008944-1039058	X3	Ultra43	No orthologs
SLC12A7	✓	5:1050499-1112172	X3	Ultra43	2:56586378-56660224
SLC6A19	✓	5:1201710-1225232	X3	Ultra43	2:87994869-88015659
SLC6A18	✓	5:1225470-1246304	X3	Ultra43	2:88017784-88041572
TERT	✓	5:1253262-1295184	X3	Ultra43	2:88046748-88076214
CLPTM1L	✓	5:1317859-1345214	X3	Ultra43	2:88089596-88119806
SLC6A3	✓	5:1392909-1445545	X3	Ultra43	No orthologs
LPCAT1	✓	5:1456595-1524092	X3	Ultra43	2:88131365-88185988
MRPL36	✓	5:1798500-1801480	GENE NOT FOUND		2:88288566-88290305
NDUFS6	✓	5:1801514-1816719	X3	X3	2:88290443-88295386
IRX4	✓	5:1877541-1887350	GENE NOT FOUND		2:89430034-89435456
IRX2	✓	5:2745959-2752969	X3	X3	2:88764965-88925629
C5orf38	✓	5:2752245-2755508	GENE NOT FOUND		No orthologs
IRX1	✓	5:3596168-3601517	X3	X3	No orthologs
ADAMTS16	✓	5:5140443-5320417	X3	X3	No orthologs
KIAA0947	✓	5:5420777-5490347	X3	X3	2:56483022-56486550
MED10	✓	5:6371994-6378707	X3	X3	2:82138749-82142478
UBE2QL1	✓	5:6448736-6495022	X3	X3	2:82108101-82123122
NSUN2		5:6599352-6633404	?	3965	2:82065202-82082124
SRD5A1		5:6633456-6669675	X1	X1	2:82049800-82064935
PAPD7		5:6714718-6757161	X3	22	2:81985384-82031023
ADCY2		5:7396321-7830194	X3	22	2:81488366-81707583
C5orf49		5:7830491-7851603	GENE NOT FOUND		No orthologs
MTRR		5:7851299-7906138	X3	22	2:81428975-81461323
FASTKD3		5:7859272-7869150	GENE NOT FOUND		2:81462094-81468670
SEMA5A		5:9035138-9546187	X3	22	2:80865563-81117201
TAS2R1		5:9629109-9630463	?	Ultra450	No orthologs
FAM173B		5:10226442-10250009	X3	22	2:80506041-80513637

CCT5		5:10250033-10266524	**X3**	22	2:80497716-80505714
CMBL		5:10275987-10308138	**X3**	22	2:80448704-80455278
MARCH6		5:10353815-10435491	**X3**	22	2:80359033-80400010
ROPN1L		5:10441636-10472141	**X3**	22	2:80347518-80353846
ANKRD33B		5:10564442-10650308	**X3**	22	2:80234551-80247583
DAP		5:10679342-10761384	**X3**	22	2:80172659-80218073
CTNND2		5:10971952-11904155	**X3**	22	2:79796404-80089401
DNAH5		5:13690440-13944652	**X3**	22	2:78506658-78616795
TRIO		5:14143811-14532235	**X3**	22	2:78186717-78368832
FAM105A		5:14581884-14615073	**X3**	22	2:78141456-78154083
FAM105B		5:14664773-14699820	**X3**	22	2:78108741-78120417
ANKH		5:14704910-14871887	**X3**	22	2:77989746-78093015
FBXL7	✓	5:15500305-15939900	**X3**	X3	2:77575673-77581526
MARCH11	✓	5:16067248-16180871	**X3**	X3	2:77494808-77525938
ZNF622	✓	5:16451628-16465901	**X3**	X3	2:77430792-77437335
FAM134B	✓	5:16473147-16617167	**X3**	X3	2:77364777-77426979
MYO10	✓	5:16665395-16936372	**X3**	X3	2:77249405-77349707
BASP1	✓	5:17217669-17276943	**X3**	X3	2:76998742-76999476
CDH18		5:19473060-20575982	**X3**	X2	2:75786318-75963608
CDH12		5:21750777-22853731	**X3**	X2	2:74675423-74831325
PRDM9		5:23507264-23528706	GENE NOT FOUND		No orthologs
C5orf17		5:23951457-24178372	GENE NOT FOUND		No orthologs
CDH10		5:24487209-24645087	**X3**	X2	2:73523999-73621549
CDH9		5:26880709-27121257	**X3**	X2	2:72557369-72626948
CDH6		5:31193857-31329253	**X3**	X2	2:70478950-70593731
DROSHA		5:31400604-31532303	**X3**	X2	2:70380481-70449443
C5orf22		5:31532373-31555165	**X3**	X2	2:70369275-70380493

[a] Protein-coding genes that have symbols assigned by HUGO Gene Nomenclature Committee (http://www.genenames.org/) and are found in 31.5 MB terminal region of the p-arm of chromosome 5 are listed. All chicken orthologs of these genes are located on chicken chromosome 2. The genes downstream of this region on the p-arm have their chicken orthologs localized on the chromosome Z. Genes which orthologs are not in current platypus genome assembly are shown in grey ink.

[b] Genes are shown in the Figure 5d.

[c] Chromosomal locations determined previously [1, 2] and in this work (genes indicated by yellow background) are listed. Question marks indicate platypus genes that have not yet been localized to chromosomes.

1. Rens W, O'Brien PCM, Grützner F, Clarke O, Graphodatskaya D, Tsend-Ayush E, Trifonov VA, Skelton H, Wallis MC, Johnston S, et al: **The multiple sex chromosomes of platypus and echidna are not completely identical and several share homology with the avian Z.** *Genome Biol* 2007, **8:**R243.
2. Veyrunes F, Waters PD, Miethke P, Rens W, McMillan D, Alsop AE, Grützner F, Deakin JE, Whittington CM, Schatzkamer K, et al: **Bird-like sex chromosomes of platypus imply recent origin of mammal sex chromosomes.** *Genome Res* 2008, **18:**965-973.
